# The Heterogeneous HLA Genetic Makeup of the Swiss Population

**DOI:** 10.1371/journal.pone.0041400

**Published:** 2012-07-25

**Authors:** Stéphane Buhler, José Manuel Nunes, Grazia Nicoloso, Jean-Marie Tiercy, Alicia Sanchez-Mazas

**Affiliations:** 1 Laboratory of Anthropology, Genetics and Peopling History, Department of Genetics and Evolution - Anthropology Unit, University of Geneva, Geneva, Switzerland; 2 Foundation Swiss Blood Stem Cells, Bern, Switzerland; 3 National Reference Laboratory for Histocompatibility, University Hospital Geneva, Geneva, Switzerland; Instituto de Higiene e Medicina Tropical, Portugal

## Abstract

This study aims at investigating the HLA molecular variation across Switzerland in order to determine possible regional differences, which would be highly relevant to several purposes: optimizing donor recruitment strategies in hematopoietic stem cell transplantation (HSCT), providing reliable reference data in HLA and disease association studies, and understanding the population genetic background(s) of this culturally heterogeneous country. HLA molecular data of more than 20,000 HSCT donors from 9–13 recruitment centers of the whole country were analyzed. Allele and haplotype frequencies were estimated by using new computer tools adapted to the heterogeneity and ambiguity of the data. Non-parametric and resampling statistical tests were performed to assess Hardy-Weinberg equilibrium, selective neutrality and linkage disequilibrium among different loci, both in each recruitment center and in the whole national registry. Genetic variation was explored through genetic distance and hierarchical analysis of variance taking into account both geographic and linguistic subdivisions in Switzerland. The results indicate a heterogeneous genetic makeup of the Swiss population: first, allele frequencies estimated on the whole national registry strongly deviate from Hardy-Weinberg equilibrium, by contrast with the results obtained for individual centers; second, a pronounced differentiation is observed for Ticino, Graubünden, and, to a lesser extent, Wallis, suggesting that the Alps represent(ed) a barrier to gene flow; finally, although cultural (linguistic) boundaries do not represent a main genetic differentiation factor in Switzerland, the genetic relatedness between population from south-eastern Switzerland and Italy agrees with historical and linguistic data. Overall, this study justifies the maintenance of a decentralized donor recruitment structure in Switzerland allowing increasing the genetic diversity of the national—and hence global—donor registry. It also indicates that HLA data of local donor recruitment centers can be used as reference data in both epidemiological and population genetic studies focusing on the genetic history of present European populations.

## Introduction

The genes of the major histocompatibility complex (MHC) in humans, or HLA genes, are the most polymorphic in the human genome [1]. They code for cell-surface molecules subdivided into two functionally distinct classes, HLA class I and class II, which play a central role in immunity by presenting antigen-derived peptides to T-cell receptors, the binding of which triggers the immune response to defend the organism. On the other hand, due to the very high level of molecular variation at multiple HLA genes making each individual genetically unique, HLA is responsible for graft rejection in organ and hematopoietic stem cell transplantation (HSCT).

In the last decades, the successful use of highly matched unrelated volunteer donors for HSCT, i.e. a 10/10 allelic match for HLA-A, -B, -C, -DRB1, -DQB1 loci [2], [3], [4], has stimulated the development of many national bone marrow (stem cells) registries. Currently, more than 18 million volunteer stem cell donors and cord blood units are registered in the Bone Marrow Donor Worldwide (BMDW) databank (http://www.bmdw.org). However, due to the huge numbers of HLA alleles and haplotypes showing variable frequencies in human populations worldwide, the recruitment of highly compatible donors still needs to be optimized. One obvious solution is to increase the donor pool size [5], [6], [7], [8], thus enhancing the HLA matching probability for a given patient. A complementary approach is to refine the recruitment by using information on the patterns of HLA diversity observed worldwide [9], [10], [11]. A close collaboration between transplantation laboratories and population geneticists investigating HLA genetic diversity in human populations has thus been developed, and has recently been strengthened by the support of several research foundations, among which are the SER and FNS in Switzerland and the ESF COST in Europe On one side (transplantation), the question addressed is to know whether distinct donor stem cell registries or distinct regional recruitment centers within a given registry show significant differences in HLA genetic variation, as this might help to favor a specific recruitment strategy. Indeed, if the amount of HLA variability observed in a few centralized recruitment centers appears to be significantly lower compared to that contributed by many distinct regional centers taken together, the choice of a centralized recruitment strategy would reduce the chances of finding compatible donors for new patients coming from outside the recruited regions. On the other side (population genetics), a key issue is to know whether the HLA genetic variation observed in a stem cell registry may be considered to describe the variation of a population at equilibrium. In this case, indeed, registry data would be useful for studies aiming at reconstructing human peopling history, given that the HLA polymorphism is a relevant indicator of past human migrations despite its crucial immunological function making it a target of natural selection [12], [13], [14], [15], [16], [17]. What makes a special interest of stem cell registries compared to the classical “anthropological data” analyzed in successive International Histocompatibility and Immunogenetics Workshop [18], [19], [20], [21] is their high sample size (several thousands to hundreds of thousands of individuals typed for at least the 3 highly polymorphic HLA-A, -B and -DRB1 loci): indeed, due to the ever increasing number of new HLA alleles identified [22], [23], a thorough characterization of the global HLA genetic variation through accurate frequency estimation and the application of powerful statistical tests [24], [25] needs the use of much higher sample sizes than those generally available in anthropological studies.

In this study, the Swiss Blood Stem Cells (SBSC) registry has been used to explore in detail the HLA genetic variation throughout Switzerland, for which few results have been published until now [26], [27], [28]. While encompassing a restricted geographic area, Switzerland is a region of great interest in Europe from a genetic point of view, as it is centrally located and embraces two very distinct environments, north/north-western lowlands (the Central Plateau) and south/south-eastern highlands (the Alps), in addition to another softly mountainous region in the north (the Jura). Therefore, this country may lie at the boundary of an abrupt genetic variation of HLA frequencies previously observed between north-western and south-eastern Europe, and tentatively explained by the presence of the Alps as a geographic barrier to gene flow [26], [29]. Moreover, Switzerland is characterized by an important cultural diversity, with three main languages spoken, French, German and Italian, and a fourth minor national language, Romansh. This represents another possible source of genetic variation related to its complex history. To sum up, Switzerland was created in the 13^th^ century (1291) thanks to the union of 3 German-speaking cantons, Uri, Schwytz and Unterwald in central Switzerland. The origin of German languages in Switzerland dates to the beginning of the 5^th^ century, when the region was invaded by the *Alemannis* (Germanic tribes originating from the Elbe and Main rivers regions) from the East, forcing the replacement of Latin spoken during the Roman Empire dominating the Helvetian territory from the 1^st^ century BC (Before Christ) to the 4^th^ century AD (Anno Domini). From the 6^th^ to the 7^th^ century, the *Alemanni* progressed westwards along the Swiss Plateau, a migration which finally drew (with slight modifications in the 15^th^ century) the present northern and western linguistic boundaries with Romance languages (formerly Franco-Provençal, a Romance language emerging from a Gallo-Roman variety of Latin, and which was completely replaced by French in the 16^th^ century). The southern French-German boundary was later created by *Alemannis'* invasion (i.e. Walsers) of Wallis (Valais) in the 11^th^ century, with a further Germanic extension in the 15^th^ century. The Rhaeto-Romance Romansh languages evolved through the contact of Latin with the language (derived from the Etruscan) spoken by the Raeti, an Alpine population established in the Graubünden area since at least 500 BC. The origin of Italian speakers in Switzerland dates from the 15th century when the Swiss cantons Uri and Obwald invaded the Italian Leventine region south to the Alps, followed by the other regions of current Ticino.

In the present work, HLA data available for more than 20,000 Swiss bone marrow donors were analyzed statistically by using several algorithms accommodating typing ambiguities (either computer programs already available [21], [30], [31], [32] or developed specifically). Allelic and haplotypic frequencies were estimated and Hardy-Weinberg equilibrium was tested both for the whole set of individuals included in the national registry and for each of 11 to 13 geographic regions (single or groups of neighboring “Swiss cantons”). The HLA genetic profiles observed in the different regions were then compared by taking into account both the peculiar geographic landscape and the cultural (linguistic) diversity of this country. As explained above, two main questions were addressed: a) do we detect significant HLA genetic variation in Switzerland, which would be relevant for donor recruitment strategies in stem cell transplantation? and b) does the observed HLA genetic landscape of Switzerland reflect traces of the history of the country? The present study provides affirmative answers in both cases.

## Results

### Gene diversity and Hardy-Weinberg equilibrium (HWE) tests

As expected for highly polymorphic HLA genes, gene diversity (here measured by the estimated heterozygosity) is very high and often close to its maximum value, with small differences among recruitment centers: 0.841 to 0.894 for HLA-A, 0.944 to 0.968 for HLA-B, 0.912 to 0.925 for HLA-C, 0.912 to 0.926 for HLA-DRB1 and 0.871 to 0.897 for HLA-DQB1 (Supporting Information File S1). Considering recruitment centers individually, the null hypothesis of Hardy-Weinberg equilibrium is never rejected (5% level) except for BE (p = 0.046), BS (p = 0.0023) and ZH (p = 0.0013) at HLA-B, and AA (p = 0.021), GE (p = 0.036), LG (p = 0.039) and ZH (p = 0.0041) at HLA-DRB1, with only 3 centers (BS and ZH at HLA-B and ZH at HLA-DRB1, respectively) remaining significant after Bonferroni's correction for multiple tests on the 5 HLA loci (Supporting Information File S1). Noteworthy is the fact that BS and ZH are (with GE) the most populated cities of Switzerland and count the highest proportion of foreign immigrants (more than one third of the population). This may explain HWE deviations in the corresponding centers.

Considering the Swiss national registry as a whole, a very distinct pattern is observed. The null hypothesis of HWE is rejected at 4 of 5 loci (HLA-A with p = 3.6E-08, HLA-B with p = 1.4E-10, HLA-DRB1 with p = 2.7E-10 and HLA-DQB1 with p = 0.023, the p-values for HLA-A, -B and -DRB1 remaining highly significant after correction for multiple tests on the 5 loci). HLA-C is the exception (p = 0.9). This strongly suggests the existence of a population structure in Switzerland with significant heterogeneity among recruitment centers, as described below on the basis of allele and haplotype frequencies. Therefore, HLA frequencies estimated at the Swiss national registry level (which are here given for informative purposes) are not in equilibrium and should not be relied on as characteristic of a “Swiss population”. Their analysis actually demonstrates that Switzerland is genetically heterogeneous at the HLA genomic region.

### Allele and haplotype frequencies

HLA-A, -B, -C, -DRB1 and -DQB1 allele frequencies estimated both in each recruitment center taken individually and in the whole Swiss national registry (all regional data pooled together) are given in Supplementary Information File S1. The practice in the manuscript is to provide the full allele name, including the HLA prefix, for the first allele occurrence in a sentence and to omit the prefix for the other alleles cited in the same sentence, for sake of brevity. HLA-A*02 is the most frequent HLA-A allele in Switzerland, with frequencies ranging from 24.1% to 34.7%. Depending on the recruitment center, HLA-A*01:01/04N/22N, A*03 and A*24 are the second, third or fourth most frequent alleles, respectively, with values ranging from 7.2% to 14.4%. Concerning HLA-B, the most frequent alleles are HLA-B*07:02/44/49N/58/59/61 and B*51:01/11N/30/32/48/51, with frequencies ranging from 4.6% to 13.8%. Other common alleles (i.e. alleles reaching a frequency of 6% in at least one recruitment center) are HLA-B*08:01/19N, B*15:01/102/104/140/146, B*18:01/17N, B*35:01/40N/42/57/94, B*44:02/19N/27, B*44:03 and B*57:01. HLA-C*07:01/06/18/52 is the most frequent HLA-C allele, with frequencies ranging from 12.3% to 16.7%. The second most frequent HLA-C allele is HLA-C*04:01/09N/28/30, with frequencies ranging from 10.5% to 15.6%. Depending on the recruitment center, HLA-C*06:02 and C*07:02/50 are the third or fourth most common alleles, with values ranging from 7.2% to 14.2%. Concerning HLA class II loci, HLA-DRB1*07 (12% to 15.2%), DRB1*04 (8.8% to 14%) and DRB1*15:01 (7.2% to 13.2%) are the most frequent HLA-DRB1 alleles, and HLA-DQB1*03:01/09/19/21 (17.5% to 27.2%), DQB1*06:02 (6.3% to 15.7%), DQB1*03:02 (3.3% to 15.6%), DQB1*02:01 (6.1% to 13.4%) and DQB1*05:01 (7.9% to 13.1%) the most frequent HLA-DQB1 alleles, respectively.

According to the results presented above, HLA frequencies appear to vary significantly across Switzerland; for example, HLA-A*02 exhibits up to 10% frequency differences among recruitment centers. To better depict the variation observed across Switzerland, box-and-whisker diagrams are presented in Supporting Information File S2 for all alleles observed at a frequency equal or above 5% in at least one recruitment center. Considering the 5 HLA loci together, File S2 shows that one to several recruitment centers behave as « outliers », i.e. they exhibit diverging allele frequencies, in about half of the diagrams. Among them, Lugano (LG) is the most frequent outlier, followed by Lausanne (LS), Graubünden (GR) and Luzern (LU). For some alleles, the frequency estimated for the whole national registry is close or almost equal to the median value of the centers taken separately, but in some other cases these values are quite distinct (in particular at the HLA-B locus). Supporting Information File S3 provides a complementary view of the HLA variation across Switzerland: it depicts allele frequencies (equal or above 3%) within each recruitment center, all centers being classified into 3 main geographic areas, the Western central plateau and Jura, the Eastern central plateau and Jura, and the Alps. Interestingly, some alleles exhibit marked differences between the central plateau and Jura, on one side, and the Alps, on the other side, notably HLA-B*51:01/11N/30/32/48/51, C*03:04, C*14:02, DRB1*11:01, DBQ1*03:01/09/19/21 and DQB1:03:02.

**Figure 1:**
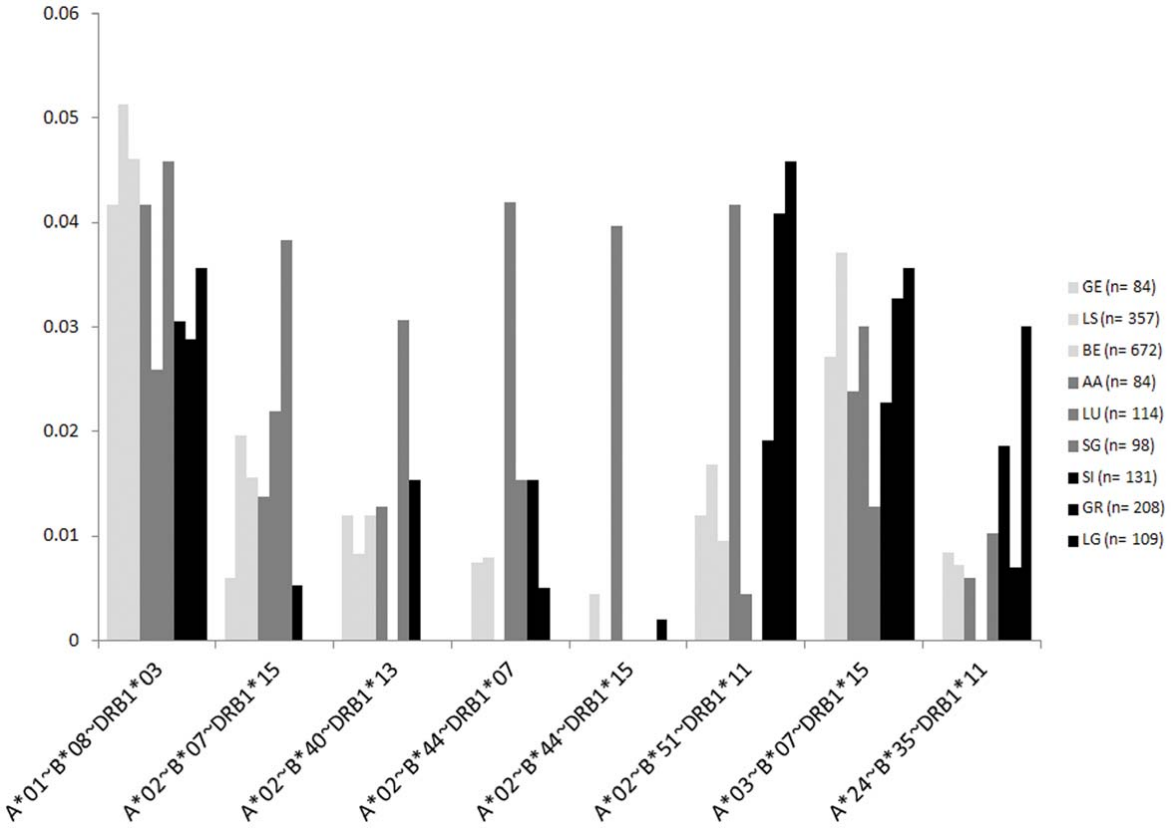
A-B-DRB1 haplotype frequencies within each recruitment region. Only haplotypes with a frequency ≥3% in at least one recruitment region are listed. Geographic regions are indicated by different colors on the graphic: Western central plateau and Jura (light grey), Eastern central plateau and Jura (middle grey), Alps (black). AA: Aargau-Solothurn, BE: Bern, BS: Basel, GE: Genève, GR: Graubünden, LG: Lugano (Svizzera Italiana), LS: Lausanne (Vaud), LU: Luzern (Zentralschweiz), SG: St. Gallen (Nordost-Schweiz), SI: Sion (Valais) and ZH: Zürich.

Haplotype frequencies at 2, 3 and 4 loci are listed in Supporting Information File S4, with frequency thresholds of 3% (A-B-C-DRB1) and 1% (3-loci and 2-loci data) chosen to optimize accuracy. Indeed, sample sizes are much lower than 1,000 individuals in the regional centers taken individually. Therefore, in view of the complexity of the HLA polymorphism, frequencies below these thresholds may not describe real frequencies in the population. Moreover, very low-frequency haplotypes are often artifacts of the EM algorithm and may not be really present in the population (see Discussion). The 5 A-B-C-DRB1 haplotypes exhibiting the highest frequency in at least one of the recruitment centers are HLA-A*01∼B*08∼C*07:01/06/18/52∼DRB1*03 (frequency varying from 1.95% to 6%), A*02∼B*07∼C*0702/50∼DRB1*15 (0% to 4.67%), A*03∼B*07∼C*07:02/50∼DRB1*15 (0% to 3.65%), A*02∼B*44∼C*05:01/03∼DRB1*15 (0% to 3.33%) and A*11∼B*35∼C*04:01/09N/28/30∼DRB1*01 (0% to 3.25%). Concerning 3-locus (A-B-C and A-B-DRB1) and 2-locus (A-B, A-C, A-DRB1, B-C, B-DRB1, C-DRB1, and DRB1-DQB1) haplotypes (File S4), the information is partially redundant with the 4-locus data and thus not commented here, except for A-B-DRB1 haplotypes reported in Figure 1. In this Figure, haplotype frequencies are again ordered according to a geographic partition of Switzerland into 3 main areas, the Western central plateau and Jura, the Eastern central plateau and Jura, and the Alps. Two striking features are the general heterogeneity of A-B-DRB1 frequencies across different recruitment centers, and the peculiarity of Lugano (LG) for several haplotypes. For example, HLA-A*02-B*51-DRB1*11 and A*24-B*35-DRB*11 frequencies are at least 2 times higher in LG than in other regions, excepted Aargau-Solothurn (AA) and Graubünden (GR) for the former and Sion (SI) for the latter.

Box-and-whisker diagrams are depicted in Supplementary File S2 for haplotypes with frequencies above or equal to 3% in at least one center. Here again, one to several regional centers behave as outliers for almost half of the haplotypes, with Lugano (LG) as the most frequent outlier, followed by Geneva (GE), Luzern (LU), Aargau-Solothurn (AA) and St. Gallen (SG). As seen for the alleles, the frequency estimated for the whole national registry is sometimes quite distinct from the median value for the regions taken individually (this is especially true for some A-B-C-DRB1, A-B-C, A-B, A-C, A-DRB1 and B-C haplotypes), including four occurrences for A-B-C-DRB1 and one for A-B-C where the frequency for the whole registry is a significant outlier.

### Ewens-Watterson (EW) neutrality tests

According to Ewens-Watterson's test, we do not observe any significant deviation from neutrality at the HLA-A locus, while there is at least one significant rejection (after correction for multiple tests) toward an excess of heterozygotes at the other loci (in 4 Swiss regions at HLA-B (AA, GE, LS, SG), 5 at HLA-C (AA, GE, SG, SI, ZH), 6 at HLA-DRB1 (AA, CF, GE, LS, SG, SI) and 3 at HLA-DQB1 (AA, BS, SG), respectively (Supplementary Information S5).

### Tests of global linkage disequilibrium (LD) and gametic association between alleles

The null hypothesis of no global linkage disequilibrium between different pairs of HLA loci has been tested in 11 recruitment centers (Table 1). No significant LD was detected between HLA-A and loci B, C and DRB1, while pairs B-C, B-DRB1, C-DRB1 and DRB1-DQB1 showed significant LD (the latter in all centers even after Bonferroni's correction for multiple tests on 10 centers, ZH being not included in the analysis due to HWE rejection). Standardized residuals computed for the most common two-locus haplotypes (with an estimated frequency above or equal to 3% in at least one Swiss center) corroborate the global tests, with only a few significant haplotypes for pairs A-B, A-C and A-DRB1 in some recruitment centers, and many more cases for pairs B-C and DRB1-DQB1, pairs B-DRB1 and C-DRB1 showing an intermediate profile (Supporting information File S6). Interestingly, significant haplotypes involving the A locus (e.g. HLA-A*01-B*08, A*03-B*07, A*01-DRB1*03 and A*03-DRB1*1501) are among the most frequently observed in European populations [33].

**Table 1:** List of registry centers exhibiting significant gametic association (linkage disequilibrium LD) for different pairs of HLA loci.

HLA loci pairs	A-B	A-C	A-DRB1	B-C	B-DRB1	C-DRB1	DRB1-DQB1
	(BE)			AA	(AA)	(AA)	AA
				BE	BE	BE	BE
				GE	GE	BS	BS
				GR	GR	(GE)	GE
Centers with significant LD				(LG)	LS	(GR)	GR
				LS	LU	(LS)	LG
				LU	(SG)	LU	LS
				SG	(SI)	SG	LU
				SI			SG
							SI
Total number of significant rejections (total number after Bonferroni's correction within brackets)	1 (0)	0 (0)	0 (0)	9 (8)	8 (5)	8 (4)	10 (10)

Centers which are not significant after Bonferroni's correction (for the number of centers tested) are indicated within brackets. BS and ZH are not considered when B locus is involved (because of HWE rejection), as well as ZH at HLA-DRB1 for the same reasons.

An indirect way of looking at the non-random association of alleles between two loci is to compute the cumulative frequency shared by the most common haplotypes in the data (here haplotypes with an estimated frequency above or equal to 3%). To summarize this information for each pair of loci, across recruitment regions (i.e. after removing centers rejecting significantly HWE at HLA-B and -DRB1), medians were computed. The median of the cumulative frequencies for the most common haplotypes is 31.9% for A-B, 49.5% for A-C, 40.8% for A-DRB1, 60.1% for B-C, 29% for B-DRB1, 31.9% for C-DRB1 and 89.4% for DRB1-DQB1. These values fit well with the results described above, and suggest that DRB1-DQB1 haplotypes behave as “LD blocks” transmitted with a low proportion of (if any) recombinations across generations (most DRB1-DQB1 haplotypes detected are both frequent and with alleles in strong gametic association). At the other extreme, the low (<50%) cumulative frequencies for the pairs involving HLA-A fit better with a pattern of non-random association of alleles, eventually coupled with selective advantage of a few particular haplotypes.

Gametic association was not tested for more than 2 loci (for theoretical reasons), but the median values of the cumulative frequencies for 3 and 4-locus haplotypes are, as expected, much lower than for 2 loci, except for A-B-C: 35.7% for A-B-C, 13.5% for A-B-DRB1 and 15.1% for A-B-C-DRB1.

### Multidimensional scaling analysis (MDS)

**Figure 2:**
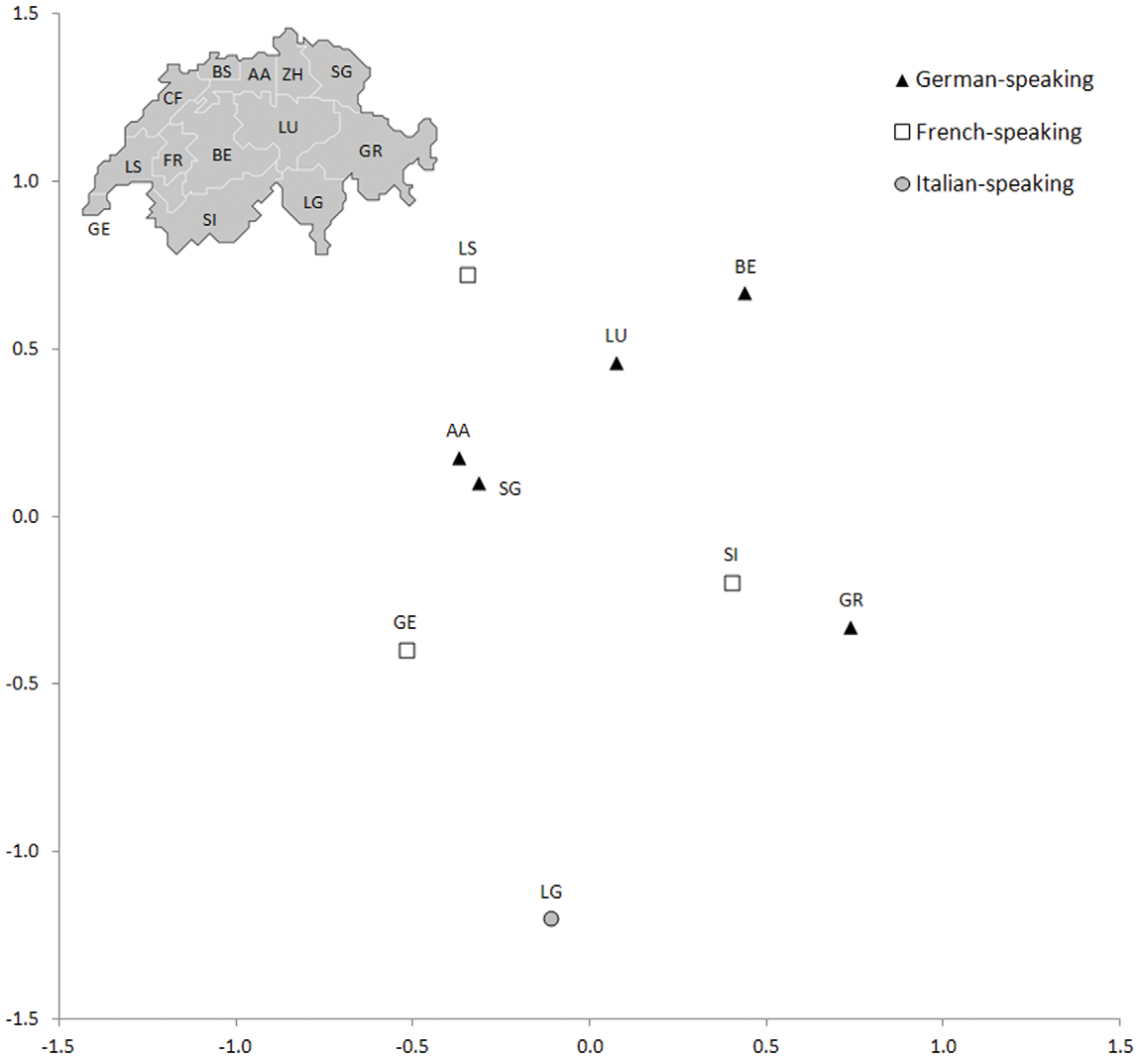
Multidimensional scaling (MDS) analysis of 9 Swiss regions based on mean Reynold's genetic distances computed for 5 HLA loci (HLA-A, -B, -C, -DRB1 and -DQB1). Symbols refer to the language spoken in each region. The stress value is 0.142 (indicating a rather good fit of the graphical representation to the original distance matrix). AA: Aargau-Solothurn, BE: Bern, BS: Basel, GE: Genève, GR: Graubünden, LG: Lugano (Svizzera Italiana), LS: Lausanne (Vaud), LU: Luzern (Zentralschweiz), SG: St. Gallen (Nordost-Schweiz), SI: Sion (Valais) and ZH: Zürich.

Mean Reynolds' genetic distances on the 5 HLA-A, -B, -C, -DRB1 and -DQB1 loci were computed among 9 recruitment regions and plotted using multidimensional scaling analysis (MDS, Figure 2). The stress value of 0.14 indicates a rather good fit of the representation compared to the original distance matrix and falls within the range of values usually obtained for HLA data. A combination of two main patterns emerges from the projection, as both the geographic and the linguistic structures of Switzerland exhibit some correlations with the groupings of populations. Indeed, recruitment centers located in the south-eastern part of Switzerland (GR and especially LG) segregate in an extreme position (right-bottom part) of the projection compared to the other centers (note that the Jura is not represented here due to the low number of donors for CF). GE and SI, two cantons located in southern Switzerland (western and central parts, respectively), segregate in an intermediate position between GR and LG, on one side, and the other centers (AA, BE, LS, LU, SG), on the other side. By considering linguistic criteria, we also note that German-speaking centers (except GR) are projected in the central upper part of the MDS, and the Italian-speaking center (LG) at the other extreme. French-speaking centers (GE and SI, but not LS projected in the upper part of the MDS) are intermediate. A tridimensional (3D) plot and a Principal Component Analysis (PCA) (not shown) confirm the patterns observed on Figure 2, yet with a better stress value of 0.065 and additional information on the third axis of the 3D plot: GE and LS are projected very close to each other followed by GR, while the other centers are located at the other extremity of the axis. Globally, LG appears as the most divergent center, followed by GR. This is confirmed by a higher number of significant pairwise F_ST_'s for these two centers compared to the others (Supplementary Information File S7). Except for some local/minor differences, MDS plots for individual loci and for HLA-DRB1 data at high-resolution are concordant with these results. Differences are listed in the below section with ANOVA results. It is just worth noting here that for HLA-DRB1 at high-resolution SI is more divergent than LG compared to the other centers (Supplementary File S8).

### Correlation between geography, linguistics and genetics, and genetic structure of Switzerland

**Figure 3:**
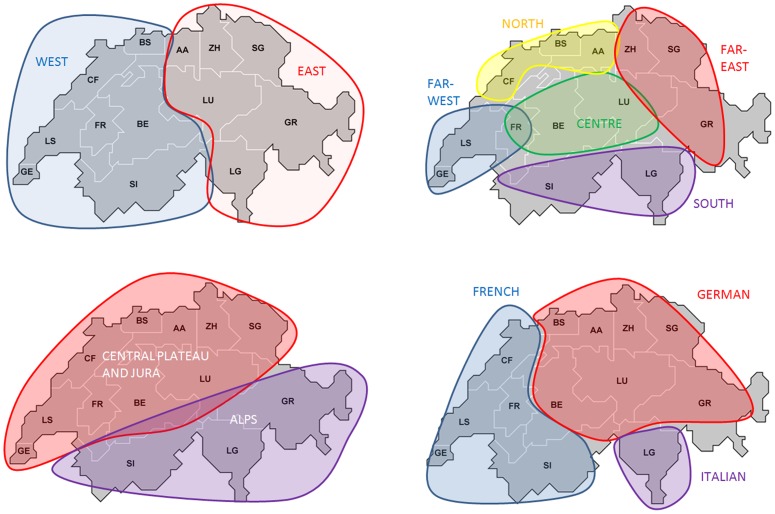
Map of the 13 regional blood transfusion services of the SBSC registry and the different geographic or linguistic groups tested by Analysis of Variance (ANOVA) on the basis of HLA allelic frequencies (see Supplementary Information S9 for more details). AA: Aargau-Solothurn, BE: Bern, BS: Basel, GE: Genève, GR: Graubünden, LG: Lugano (Svizzera Italiana), LS: Lausanne (Vaud), LU: Luzern (Zentralschweiz), SG: St. Gallen (Nordost-Schweiz), SI: Sion (Valais) and ZH: Zürich.

By testing the correlation between genetic and geographic distances among the 9 Swiss centers in HWE (Table 2), we obtained a significant r value of 0.373 (P = 0.0147) when the 5 loci were considered together. This result fits well with that of the MDS projection and is indicative of isolation by distance. A significant correlation with geography is also observed for HLA-DQB1 taken individually (Table 2). The correlation between linguistic and genetic distances is also significant (r = 0.297, P = 0.029) when the 5 loci are considered together, but not anymore when the effect of geography is accounted for in a partial 3-way Mantel test (r = 0.097, P = 0.2773). We also performed analyses of variance (ANOVA) to assess whether the observed HLA genetic diversity was significantly structured in relation to a given geographic or linguistic partition (shown in Figure 3 and in Supplementary Information File S9) of Switzerland. The combined results of ANOVA for the 5 loci are shown in Table 3 and detailed results per locus in Supplementary Information File S10. A geographic partition into 2 predefined groups - west *versus* east - is not significant (i.e. the null hypothesis H_0_: F_CT_ = 0 is not rejected). Despite a significant F_CT_ of 0.054% (P = 0.012), a geographic partition into 4 groups - far-west *versus* centre *versus* south *versus* north *versus* far-east - is not a real group structure because the proportion of genetic variation observed among the groups (F_CT_ of 0.054%) is lesser than the proportion of genetic variation observed within those groups (F_SC_ of 0.094%). The unique real significant group structure found is that of the 2 groups « Alps *versus* Plateau and Jura », with a highly significant F_CT_ index of 0.138% (P = 0.0001), almost 1.5 times the value of its F_SC_ counterpart (0.1%). For a linguistic partition into 3 groups - French *versus* German *versus* Italian -, the F_CT_ index is highly significant (P = 0.000015) but its value is only slightly greater than the F_SC_ value (0.098% versus 0.095%, respectively). Individual analyses per locus corroborate these results (the « Alps *versus* Plateau and Jura » and the linguistic partitions) for HLA-C, -DRB1 and -DQB1, but not for HLA-A and HLA-B. However, by looking at the MDS (Supplementary Information File S8), we note that the discordant result obtained for HLA-A is mostly due to a peculiar position of LG and for HLA-B to peculiar positions of GE and BE. These locus-specific peculiarities are not detected anymore when all loci are considered together.

**Table 2:** Correlation coefficient (r) between matrices of genetic and geographic distances, and genetic diversity (F_st_) among recruitment centers for each HLA locus under study.

	CTS	n	r	p-value	N	F_ST_ (%)	p-value
5 loci*	9 to 12	NA	0.373	0.0147	1	0.14	8.06E-17
HLA-A	11#	2488	0.156	0.165	1	0.14	0.0006
HLA-B	9# %	2328	0.194	0.146	1	0.26	<0.0001
HLA-C	11#	3512	0.273	0.062	1	0.08	0.0005
HLA-DRB1	12&	16732	0.255	0.087	1	0.1	<0.0001
HLA-DQB1	11#	1808	0.265	0.044	1	0.12	0.017

Abbreviations used in the table,

* : for 5 loci, we used a mean matrix of genetic distances and weighted average FST index with combined probability for HLA-A, -B, -C, -DRB1 and –DQB1 computed by Fisher's meta-analysis method.

CTS: regional services for blood transfusion.

# : CF and FR not tested because of small sample sizes.

% :: BS and ZH not included at HLA-B because of HWE rejection.

& : ZH not included at HLA-DRB1 because of HWE rejection.

n: number of donors.

N: number of groups tested.

NA: The number of donors available at each of the 5 loci is used for the computations.

**Table 3:** Weighted average genetic diversity indexes at 5 loci (HLA-A, -B, -C, -DRB1 and -DQB1), within geographic/linguistic groups (F_SC_) and among geographic/linguistic groups (F_CT_).

Regions compared	N	F_CT_ (%)	p-value	F_SC_ (%)	p-value
West vs East	2	0.0013	0.441	0.141	1.5E-12
West *vs* Centre *vs* South *vs* North *vs* East	5	0.054	0.012	0.094	3.0E-10
Alps *vs* Plateau & Jura	2	0.138	0.0001	0.1	1.9E-10
French *vs* German *vs* Italian	3	0.098	1.5E-5	0.095	8.6E-11

The meta-analysis method of Fisher has been used to compute the 5-locus combined probability for each index. Abbreviations used in the table, vs: versus, N: number of groups tested.

A complementary approach to ANOVA is to search for significant genetic boundaries by means of SAMOVA analyses (with no groups defined *a priori*). The general pattern (results not shown) is that the recruitment centers segregate successively one by one rather than within larger groups, with no congruence among the loci. Therefore, there is no statistical evidence of a main genetic boundary in Switzerland. Interestingly, however, LG is the first center to segregate at the 4 loci HLA-B, -C, -DRB1 and -DQB1, together with GR at the latter locus, in agreement with the peculiar HLA genetic profile observed in this (these) region(s).

## Discussion

This study is the first in-depth analysis of the HLA genetic landscape of Switzerland, using molecular data of more than 20,000 bone marrow donors recruited by the Swiss (SBSC) registry. The complex nature of the HLA polymorphism as well as the content of the registry itself (e.g. data gathered during a time frame of several years and characterized by mixed levels of typing resolution, see Supplementary Information S11) required the improvement of specifically adapted statistical methods and software [21], [30], [31], [32]. Indeed, a previous study on the National Marrow Donor Program (NMDP) registry demonstrated that the EM algorithm results are not skewed [34], even if the available level of resolution may depend on the HLA type (i.e. donors with more common HLA types are preferentially selected for intermediate to high resolution typing), a common situation in any registry. The main challenge was then to conserve as much allelic resolution as possible within the data prior to statistical analyses and genetic comparisons, while keeping possible biases at a minimum during frequency estimation. In theory, the EM algorithm implemented in the Gene[RATE] program package is able to deal with any amount of complex HLA data. However, one major limitation when dealing with ambiguities is when non-ambiguous typings are scarce compared to ambiguous and generic cases, as, in such situations, the output frequencies may contain many artifacts [12], [21]. Thus, to allow one to make an optimized decision on how to deal with ambiguities and mixed levels of resolution within the data prior to allelic or haplotypic frequency estimation, a program called Split-test was developed. By comparing the respective proportions of unambiguous, ambiguous, and generic cases in a set of data, it provides an automatic decision on whether it is better to take ambiguities into account in the analyses or to collapse them to a lower level of resolution. Basically, the program allows one to determine if unambiguous typings can be used for sorting out the ambiguous and generic cases (both expanded to lists of possible genotypes) during the frequency estimation process, or not. Split-test is thus a new and very useful pre-analytical tool to analyze HLA registry data, in order to get accurate allele and/or haplotype frequency estimates while keeping as much relevant information as possible. It has also the advantage of including all individuals in the process, thus avoiding possible recruitment biases (e.g. by selecting only a subset of the data according to a given criterion). Its application to the Swiss registry led to frequency outputs ranging from allelic resolution for HLA-B, -C and -DQB1 to mixed allelic/generic resolution for HLA-A, -DRB1 and two-locus haplotypes (see File S1 and S4), enabling a very detailed survey of these data.

A relatively low HLA genetic diversity is observed in Switzerland, as the F_ST_ indexes found among the recruitment regions range from 0.08% for HLA-C to 0.26% for HLA-B, which is very close to the values obtained for the Bone Marrow Donor registry of Quebec (from 0.16% to 0.32%, ongoing study) and, as expected, lower than when wider geographical (e.g. continental) regions are considered. For instance, a previous study on HLA-C measured F_ST_s ranging from 0.75% to 1.98% in two sets of European populations (i.e. 6 populations characterized at high resolution and 16 populations characterized at low resolution, respectively) and from 2.02% to 3.45% (15 populations characterized at low resolution and 10 populations characterized at high resolution, respectively) in India/Pakistan [26]. Also, the F_SC_ and F_CT_ indexes obtained through the hierarchical analyses of variance (see Table 3 and Supplementary Information File S10) are 10 to 100 times below what is usually observed for HLA genes at continental and worldwide scales. For example, in two studies with various numbers of populations tested among continents, the F_CT_ indexes vary from 3.2% at HLA-C to 9.7% at HLA-DPB1, while the Φ_CT_ indexes vary from 2.6% at HLA-B to 10.6% at HLA-DQA1 [12], [35]. This is even less compared to some non-HLA neutral genetic polymorphisms [36], [37], [38], [39].

Nevertheless, our results reveal regional heterogeneity across Switzerland, both at intra- and at inter-regional levels. First, the assumption of Hardy-Weinberg equilibrium is strongly rejected when considering the registry as a whole, with about half of the most frequent alleles and haplotypes exhibiting a significant difference in at least one of the recruitment regions compared to the rest of Switzerland (see File S2). Comparable diversity levels in HLA-A, -B and -DRB1 allele and haplotype frequencies have been reported in the French [40], German [10] and Italian [41] registries, and it is to be expected that national donor registries represent more heterogeneous collections of individuals than population samples collected during field studies in anthropology (which are generally constituted on the basis of criteria related to ethnicity). Actually, local recruitment centers may also be represented by heterogeneous samples, like, in this study, those of BS and ZH (not in Hardy-Weinberg equilibrium) which represent two main cities (besides GE) of Switzerland with high proportions of non-Swiss immigrants. In such cases, the observed HLA frequencies may not be considered as representative for the local populations and they should not be included in other analyses. The other regional centers are all in HWE despite, in some cases, rejection of selective neutrality towards an excess of heterozygotes (as shown by EW tests). This means that the excess of genetic diversity sometimes observed for different HLA loci is not great enough to deviate significantly the proportions of Hardy-Weinberg equilibrium. This is in agreement with studies suggesting that the intensity of balancing selection acting on the HLA loci is low [12], [13]. Also, our results support the hypothesis that HLA-A, for which selective neutrality is never rejected in our study, evolves very much like a neutral polymorphism [35].

Second, inter-regional genetic comparisons reveal both a significant correlation between HLA genetic variation and geography in Switzerland (see Results and below) and a high number of significant pairwise F_ST_'s among centers (Supplementary Information File S7). Noteworthy, recruitment regions located south of the Alps, i.e. Ticino (LG) in particular but also Graubünden (GR), exhibit a pronounced genetic differentiation compared to the other centers. Also, a significant group structure is found through ANOVA for the « Alps *versus* Plateau and Jura » partition of Switzerland, with a significant F_CT_ index higher than the F_SC_ counterpart (0.138% versus 0.1%). The heterogeneity of the HLA genetic makeup of the Swiss population may thus be explained by a general isolation-by-distance pattern (explaining the significant correlation with geography), with a sharper genetic boundary in the Alps area (explaining the « Alps *versus* Plateau and Jura » group structure). Of course, the ANOVA results have to be taken with caution as an irregular sampling scheme (i.e. a non uniform coverage of the different regions of Switzerland, as is the case here) may also result in a significant group structure suggesting population boundaries where none occurs [42]. However, being one of the greatest mountain ranges of Europe, the Alps are expected to be a potential barrier to gene flow in Switzerland, and our results also fit with previous conclusions based on HLA-C and -DRB1 diversity studies at the European level [26], [29]. Besides geography, a linguistic boundary could also be incriminated to explain the divergence of the Italian-speaking canton, based on the significant correlation found between genetic and linguistic variation when all Swiss cantons are compared (see Results). However, non-significant partial correlation coefficients are found between genetic and linguistic distance matrices when geography is held constant (see Results), which is explained by the fact that the French, German and Italian-speaking recruitment regions are geographically clustered (see Figure 3 and Supplementary Information File S9). Although our approach has to be taken with caution due to a very rough definition of linguistic distances (see Material and methods), the results indicate that geographic differentiations may be driving the linguistic signal and that cultural subdivisions may not be the main genetic differentiation factor in Switzerland.

It is worth comparing the observed genetic variation in Switzerland to what is found at a broader geographic level. Detailed analyses by the AHPD component of the 15^th^ International Histocompatibility/Immunogenetics Workshop detected a sharp increase, sometimes more than twice, of HLA-B*51 and DRB1*11 frequencies in southeastern European populations compared to northern, southwestern and central Europe [21]. Interestingly, LG, SI and GR exhibit the highest frequencies for the HLA-B*51 and DRB1*11 alleles in the registry (10–16.3% for HLA-B*51 and 16–19.1% for DRB1*11, respectively, see Files S1 and S3). Also, the HLA-A*02-B*51-DRB1*11 haplotype, which is the most frequent in Ticino, ranks second in Piedmont [41] after A*01-B*08-DRB1*03. The previously observed HLA genetic differentiation of southeastern Europeans, including populations from both Italy and the Balkans, thus extends to the 3 southeastern regions of Switzerland encompassing the Alps. They are also more pronounced for LG (Italian-speaking canton) and GR (German, Italian and Romansh-speaking) than for SI (French and German-speaking). As described in the Introduction, LG and GR are linked to Northern Italy from a historical and cultural point of view. Indeed, Lombard dialects and Italian, both spoken in LG and GR, and Romansh, spoken in GR, belong to the Italic>Romance>Italo-Western branch of the Indo-European linguistic family and are thus closely related to each other [43]. Lombard dialects are mainly spoken in the central part of Northern Italy (Lombardy), while Ladin, the closest language to Romansh, and Friulian, also part of the Gallo-Rhaetian language family, are spoken in Northeastern Italy (Dolomites mountains of Trentino, South Tyrol and the province of Belluno for Ladin; and the Friuli region for Friulian, respectively). Apart from Romansh, Italian and Lombard, the GR canton is mainly inhabited by German native speakers, some of them speaking Walser dialects in the highland valleys. The Walsers are named after the Wallis (Valais, SI), where they lived 1,000 years ago and from where they spread to other areas of the Alps in the 12^th^ and 13^th^ centuries [44]. Today, Walsers are also located in neighboring countries like Northwestern Italy (Piedmont), Western Austria (Tyrol) and Liechtenstein. Therefore, despite failing to show a significant correlation between genetic and linguistic variation at the level of the whole country, this study reveals that the complex history of Switzerland explains in great part its current HLA genetic landscape.

The HLA-A-B-DRB1 haplotype frequencies found in this study are very close (although with regional variations) to previous estimations done in 2008 by the ZKRD (the German donors registry) [7] and in 2010 by the BMDW on the whole Swiss data pooled together [45], and this is also true for haplotypes including the HLA-C locus. However, our approach has the advantage of taking ambiguities into account to estimate frequencies and is thus much more precise than in other studies. In any case, it is worth stressing that multi-locus haplotype frequencies should be considered with caution when such highly polymorphic genes are analyzed. As explained in a recent study [46], HLA haplotype frequencies may be poorly estimated when data include ambiguous typings, distant genes (which may not be in LD with the other loci, like HLA-A) and/or more than two loci. Here, we find that the cumulative frequencies of the most common haplotypes are very low for 3 or more loci (less than 20% of the total frequency) and the number of haplotypes in the estimation output exceeds by far the number of individuals tested, due to the generation of huge numbers of haplotypes with very low or artifact frequencies. This is a well-known and expected consequence of using incomplete information (i.e. unknown gametic phase) with the EM algorithm and the situation is also complicated by the presence of very large numbers of alleles at the loci studied [47]. Therefore the presence/absence or the frequency of multi-locus haplotypes should not be over-interpreted, in particular as regards donor search in transplantation. Alternative strategies based on phenotype rather than haplotype frequencies might help refining the recruitment of donors and provide better predictions as, for example, in the acceptable mismatch program of Eurotransplant [48].

To conclude, this study has first shown that different donor recruitment centers of Switzerland may be considered as representative of local populations at equilibrium while the whole national registry may not. It thus indicates that HLA bone marrow donor registries may be used under certain condition (i.e. Hardy-Weinberg equilibrium) as reference data in both epidemiological and population genetics studies following an anthropological perspective (like the reconstruction of human peopling history). Such data have the advantage of providing huge sample collections otherwise not conceivable. On the other hand, one should be aware that different registries may follow special recruitment policies resulting in more heterogeneous collections of data. For instance, the Swiss Cord Blood Bank (CBB, not included in this survey) puts a special emphasis on the recruitment of ethnic minorities living in Switzerland in order to improve the probability of donor-recipient matching, and this registry differs from the Volunteer Unrelated Donors (VUD) dataset [45]. Also, ethnic minorities are extensively represented in the London Cord Blood Bank while they make up only 2% of the VUD in the British Bone Marrow registry [49]. In such situations where the data are very heterogeneous on purpose, analyses focusing on scientific questions related to anthropology and human peopling history might not be recommended.

Another crucial implication of this study concerns the optimization of future recruitment strategies of HSCT donors. While this work does not deal with the specific search of matched HLA donors for a given patient in need of a transplant, it provides long-term recommendations about the best recruitment strategies to implement, as the aim is to enhance the HLA diversity available in registries. Here, the maintenance of the currently existing « decentralized recruitment » of such donors among 13 centers covering the main geographic areas of Switzerland is supported by our results, as opposed to donor recruitment centralization into one or a few blood transfusion centers of the country. Indeed, this decentralization allows increasing both the overall genetic variability of the Swiss registry and the available pool of donors with distinct HLA profiles for patients in need of a transplant. As donor search is usually processed through the BMDW database at a worldwide scale, our conclusion suggests that the same kind of recruitment strategy could be recommended in other countries to improve the BMDW genetic diversity, would such countries show a significant genetic structure. Ongoing analyses done on several European registries will provide further material to discuss this question.

Finally, this study has demonstrated the feasibility of incorporating highly complex and ambiguous data, e.g. registry data, in population genetics analyses thanks to the improvement of useful computer tools. Our future perspective is to extend this study to other countries in order to construct a detailed HLA genetic map of European populations which is one of the main goals of *HLA-NET* (the European network of the HLA diversity for histocompatibility, clinical transplantation, epidemiology and population genetics funded by COST Action BM0803).

## Materials and Methods

### Ethics statement

No submission to a local ethics committee has been necessary because volunteer donors from the national registry sign an informed consent form stating that their HLA data can be used for research in the genetic variability of human populations. The genetic material is conserved under an anonymous code.

### The Swiss registry

This study has benefited from a close collaboration between the University of Geneva (AGP lab), the Geneva University Hospital (National Reference Laboratory for Histocompatibility), and the Foundation Swiss Blood Stem Cells (SBSC) in Bern. As of August 2011, the SBSC registry included HLA genetic data for more than 32,000 donors recruited in 13 regional services for blood transfusion: Aargau-Solothurn (AA), Bern (BE), Basel (BS), La Chaux-de-Fonds (CF), Fribourg (FR), Genève (GE), Graubünden (GR), Lugano (LG), Lausanne (LS), Luzern (LU), St. Gallen (SG), Sion (SI) and Zürich (ZH) (see Figure 3 and Supporting Information File S9). These services provide a comprehensive geographic coverage of the country, although the available information corresponds to the place of residence of the donors, which may differ from their place of origin.

For the present study, the HLA data of 21,607 donors (file transmitted by the registry on the 5^th^ of May 2009) have been used: besides serological typings available for all donors at the 3 loci HLA-A, -B and -DRB1 (not used in this study), molecular typings have been performed for varying numbers of individuals at the 5 loci HLA-A (N = 2,574, with an average of 198 donors per region), HLA-B (N = 3,023 with an average of 232 donors per region), HLA-C (N = 3,615 with an average of 278 donors per region), HLA-DRB1 (N = 21,607 with an average of 1,662 donors per region) and HLA-DQB1 (N = 1,857 with an average of 143 donors per region). Although an important proportion of genotypes were reported at the allelic resolution (i.e. a DNA-based typing result consistent with a single allele as defined in the WHO HLA Nomenclature [22], [50]), part of the data contained either ambiguous allele groups (i.e. a DNA-based typing result that includes a subset of alleles sharing the first field of their allele name and that excludes some alleles sharing this field, provided as NMDP codes) or generic typings (i.e. a DNA-based typing result at the level of the first field in the HLA nomenclature, e.g. “B*44” or “B*44:XX”), see [51], [52], [53], [54] and Supplementary Information S11 for a detailed explanation of HLA typing resolution and ambiguities. In addition, from July 2008 all donors were typed at the 2nd-field resolution for HLA-DRB1 (using PCR-SSO reverse on microbead arrays, luminex technology, or PCR-SBT), which provided a subset of 1,366 donors (with an average of 105 donors per region) with high-resolution typing for HLA-DRB1.

Two regional services were characterized by insufficient number of donors (CF and FR, File S9) at HLA-A, -B, -C, -DRB1 (for the subset of donors typed after July 2008) and -DQB1. Thus, among the 13 Swiss regions, only 11 were considered for the analyses, except for HLA-DRB1 where all regions were taken into account. Several GNU/Linux scripts were used to export the original data of the SBSC registry to formats usable for population genetics analysis software (see below).

### Computer analyses

#### Treatment of typing ambiguities

Due to the incorporation of donors to the registry in a time span of several years and because of the constant improvement of typing kits to cope with the discovery of new HLA alleles, the SBSC registry contains mixed levels of typing resolution (see above). Up to now, these typing ambiguities have severely impaired the comparison of HLA molecular data among different donor registries or among different populations typed for anthropological purposes. To circumvent this major limitation, two main approaches were considered in the past [55], but none of them was totally satisfying: indeed, to recode ambiguous allele groups at a lower level of resolution may lead to an important loss of information. As shown in a previous study on HLA-C diversity in India/Pakistan [26] and reported in the Discussion, the F_ST_ is divided by two for low resolution data (2.02%) compared to high resolution (3.45%) because several informative allele frequencies are regrouped for the analysis. The alternative approach which consists in replacing ambiguous allele groups with the most common allele observed in the registry or population under study introduces a bias which may result in both under- and overestimation of many allele frequencies (as shown by an exhaustive comparative analysis done on a subset of the 13^th^ International Histocompatibility and Immunogenetics Workshop data including 30 population samples [56]).

We thus developed a computer program called Split-test to handle mixed levels of resolution at one or several loci [57]. In fact, Split-test is a generalization of the two main approaches described above, extended to avoid arbitrary withdrawal of ambiguous information (i.e. the less common alleles). As input, the program requires allelic frequencies preliminary estimated on the raw HLA genotypic data (i.e. without any prior recoding). It then compares the proportions of non-ambiguous alleles, ambiguous allelic groups and generic typings involving each generic specificity (e.g. A*02) in order to decide whether it is best to take allele ambiguities into account during the process of frequency estimation or to recode the data by using a lower level of resolution (see detailed explanations in Supplementary information S11). The Split-test approach has several advantages: (1) to keep as much information as possible prior to data analysis; (2) to avoid over- or underestimation of allele frequencies due to ambiguous data recoding; (3) to provide comparable data among the different Swiss regions.

#### Statistical analyses

Following the treatment of ambiguities described in the previous section and File S11, allele and haplotype frequencies were estimated both for the whole Swiss registry and for each regional service individually by using an Expectation-Maximization (EM) algorithm implemented in the Gene[RATE] program package accommodating ambiguous data (http://geneva.unige.ch/generate/) [30], [31], [58]. Box-and-whisker diagrams were drawn from the estimated frequencies with a GNU/Linux script and the R statistical software (http://www.r-project.org/) to depict potential outliers (i.e. regions with significantly lower or higher frequencies for a given allele or haplotype) among the different regional services. A significant departure from Hardy-Weinberg equilibrium (HWE) expectations was tested using a nested likelihood model, where the HWE model is seen as a particular case of a model that includes a parameter, i.e. an inbreeding coefficient, accounting for HWE deviations [21]. This approach does not require making assumptions about the kind of data (blank alleles, ambiguities, etc) and is therefore not restricted to HLA data. An adapted version of the classical Ewens-Watterson (EW) test for ambiguous data [21], [32] was used to assess selective neutrality at the HLA loci under study (also see Supplementary information File S5). Global linkage disequilibrium (LD) is a measure of non-random association of several pairs of alleles between two loci. For multi-allelic loci this is not the same as non-random association between individual pairs of alleles of the two loci (often named linkage disequilibrium as well, but also gametic association between two alleles). Of course, gametic associations between individual pairs of alleles of two loci may create significant global LD between these loci (for a formal discussion see [59] and references therein). In this study, global LD was tested by using a resampling procedure rather than by considering all possible gametic associations between pairs of alleles of these two loci and correcting for multiple testing (i.e. an alternative way of testing global LD). As for haplotype frequency estimations, only the pairs of loci most commonly described in the literature for registry data were analysed (i.e. Class I pairs, Class I with HLA-DRB1, and HLA-DRB1-DQB1). This approach consists in generating, from the observed ambiguous data, 1,000 random samples in which no individual has ambiguous genotypes. The observed statistic (i.e. the sum of squared differences between the observed two-locus haplotype frequencies and the two-locus haplotype frequencies expected under the null hypothesis of no LD) was compared to the empirical distribution resulting from the resampling procedure, and was considered as significant if falling above the 95% percentile. Gametic association between alleles was assessed using standardized (Pearson) residuals [60], where a value of plus or minus 2 indicates a deviation too large under the assumption of random association, i.e. a significant association. Standardized residuals are computed as the difference between the observed and the expected frequency divided by the square root of the expected frequency (i.e. this is equivalent to the square root of a chi-square contribution) and are used to determine which haplotypes are major contributors to the rejection (or not) of the null hypothesis of no gametic association.

Different recruitment centers were compared by computing Reynolds' genetic distances based on allelic frequencies [61], for each locus taken independently. Pairwise F_ST_'s between regions were tested for significance by using a non-parametric resampling procedure [62]. To summarize the results, mean pairwise genetic distances were computed for the 5 loci taken together and plotted using a multidimensional scaling (MDS) analysis [63], [64]. Comparisons between geographic, linguistic and genetic distances were done by 2-way and partial 3-way Mantel tests [65], [66]. Geographic distances were computed as the logarithms of arc-distances. Linguistic distances were approximated by choosing arbitrary values of 0 among recruitment regions speaking the same language (either French, Italian and German), of 1 between French and Italian (both belonging to the Italic branch of the Indo-European phylum [43]) and of 2 between either French or Italian and German (German being part of the Germanic branch of the Indo-European phylum). Analysis of Variance (ANOVA) was performed with Arlequin software to test the significance of the variance components associated to three levels of genetic structure: among recruitment centers (F_ST_), among recruitment centers within predefined geographic or linguistic groups (F_SC_), and among such predefined groups (F_CT_), respectively. To summarize the results found for the 5 loci taken together, weighted averages were computed for F_SC_ and F_CT_ [67], and combined probabilities were computed according to Fisher's meta-analysis method [68], [69]. SAMOVA analyses were performed to identify possible genetic boundaries among Swiss regions [70].

## Supporting Information

Supporting Information S1 Allelic frequencies, genetic diversity and tests of Hardy-Weinberg equilibrium (HWE).
(DOC)

Supporting Information S2 Box-and-whisker diagrams.
(DOC)

Supporting Information S3 Allele frequencies histograms in three main geographic areas of Switzerland.
(DOC)

Supporting Information S4 Haplotype frequencies.
(DOC)

Supporting Information S5 Ewens-Watterson's tests.
(DOC)

Supporting Information S6 Standardized residuals for two locus haplotypes.
(DOC)

Supporting Information S7 Pairwise FST's among Swiss regions.
(DOC)

Supporting Information S8 Multidimensional scaling analyses (MDS) for HLA-A, -B, -C, -DRB1 and -DQB1.
(DOC)

Supporting Information S9 Map of the 13 regional blood transfusion services of the SBSC registry and the geographic or linguistic structures tested by ANOVA.
(DOC)

Supporting Information S10 Single locus ANOVA's.
(DOC)

Supporting Information S11 HLA nomenclature, typing resolution, ambiguities and SPLIT-TEST.
(DOC)
