# Supplementary material for: The Heterogeneous HLA Genetic Makeup of the Swiss Population
Source: PLoS One. 2012 Jul 25;7(7):e41400. doi: 10.1371/journal.pone.0041400 (PMC3405111; doi:10.1371/journal.pone.0041400)
Supplement: Supporting Information S1 — Allelic frequencies, genetic diversity and tests of Hardy-Weinberg equilibrium (HWE). (DOC) [file pone.0041400.s001.doc]

**Supporting Information S1 – Allelic frequencies, genetic diversity and tests of Hardy-Weinberg equilibrium (HWE)**

Frequencies are listed in italic when the HWE null hypothesis is rejected (after Bonferroni’s correction) and, therefore, should not be considered as representative of the national or local population (here given for informative purposes).

List of abbreviations used in the below tables:

AA: Aargau-Solothurn, BE: Bern, BS: Basel, GE: Genève, GR: Graubünden, LG: Lugano (Svizzera Italiana), LS: Lausanne (Vaud), LU: Luzern (Zentralschweiz), SG: St. Gallen (Nordost-Schweiz), SI: Sion (Valais), ZH: Zürich and All: all recruitment centers pooled together.

**HLA-A**

| ALLELES | AA (n=103) | BE (n=720) | BS (n=126) | GE (n=105) | GR (n=231) | LG (n=125) | LS (n=366) | LU (n=153) | SG (n=111) | SI (n=155) | ZH (n=293) | All (n=2488) |
| --- | --- | --- | --- | --- | --- | --- | --- | --- | --- | --- | --- | --- |
| A*01:01/04N/22N | 0.1165 | 0.1278 | 0.1204 | 0.1048 | 0.0721 | 0.0744 | 0.1325 | 0.0907 | 0.1243 | 0.1146 | 0.1002 | *0.1144* |
| A*01:01N | 0 | 0 | 0 | 0 | 0.004 | 0 | 0 | 0 | 0 | 0 | 0 | *0.0007* |
| A*01:02 | 0 | 0 | 0 | 0 | 0 | 0.0093 | 0 | 0 | 0 | 0 | 0 | *0.0004* |
| A*01:09 | 0 | 0 | 0 | 0 | 0.004 | 0 | 0 | 0 | 0 | 0 | 0 | *0* |
| A*01:11N | 0 | 0 | 0 | 0 | 0.004 | 0 | 0 | 0 | 0 | 0 | 0 | *0* |
| A*01:16N | 0 | 0 | 0 | 0 | 0.004 | 0 | 0 | 0 | 0 | 0 | 0 | *0* |
| A*01:18N | 0 | 0 | 0 | 0 | 0.004 | 0 | 0 | 0 | 0 | 0 | 0 | *0* |
| A*01:25 | 0 | 0 | 0 | 0 | 0.004 | 0 | 0 | 0 | 0 | 0 | 0 | *0* |
| A*02 | 0.3472 | 0.275 | 0.2972 | 0.2476 | 0.2921 | 0.299 | 0.2594 | 0.2848 | 0.33 | 0.2411 | 0.2618 | *0.2794* |
| A*03 | 0.1004 | 0.1438 | 0.1133 | 0.1048 | 0.1224 | 0.1191 | 0.1229 | 0.1295 | 0.0893 | 0.1139 | 0.1291 | *0.126* |
| A*11 | 0.0708 | 0.0618 | 0.0476 | 0.0476 | 0.058 | 0.052 | 0.0542 | 0.049 | 0.0516 | 0.0601 | 0.051 | *0.0566* |
| A*23:01/07N/17/18 | 0.026 | 0.0292 | 0.006 | 0.0143 | 0.0032 | 0.016 | 0.0451 | 0.0294 | 0.0135 | 0.0452 | 0.0324 | *0.0291* |
| A*23:03 | 0 | 0 | 0 | 0 | 0.0032 | 0 | 0 | 0 | 0 | 0 | 0 | *0* |
| A*23:05 | 0 | 0 | 0 | 0 | 0.0032 | 0 | 0 | 0 | 0 | 0 | 0 | *0* |
| A*23:06 | 0 | 0 | 0 | 0 | 0.0032 | 0 | 0 | 0 | 0 | 0 | 0 | *0* |
| A*23:10 | 0 | 0 | 0.006 | 0 | 0 | 0 | 0 | 0 | 0 | 0 | 0 | *0* |
| A*23:11N | 0 | 0 | 0 | 0 | 0.0032 | 0 | 0 | 0 | 0 | 0 | 0 | *0* |
| A*23:16 | 0 | 0 | 0 | 0 | 0.0032 | 0 | 0 | 0 | 0 | 0 | 0 | *0* |
| A*24 | 0.0758 | 0.0931 | 0.1062 | 0.1095 | 0.112 | 0.1196 | 0.0833 | 0.0766 | 0.1081 | 0.1244 | 0.1089 | *0.0992* |
| A*25:01 | 0.0049 | 0.0222 | 0.0198 | 0.019 | 0.0043 | 0.008 | 0.014 | 0.0327 | 0 | 0.0129 | 0.02 | *0.0169* |
| A*25:05 | 0 | 0 | 0 | 0 | 0.0043 | 0 | 0 | 0 | 0 | 0 | 0 | *0* |
| A*26:01/24/26 | 0.0275 | 0.0368 | 0.027 | 0.0429 | 0.0478 | 0.0475 | 0.0497 | 0.0458 | 0.0541 | 0.0387 | 0.0243 | *0.0398* |
| A*26:03 | 0 | 0 | 0 | 0 | 0 | 0 | 0 | 0 | 0 | 0 | 0.0011 | *0.0001* |
| A*26:08 | 0.0082 | 0.0041 | 0.0067 | 0.0143 | 0.0037 | 0 | 0.0045 | 0 | 0 | 0 | 0.0024 | *0.0038* |
| A*26:21 | 0 | 0 | 0 | 0 | 0 | 0 | 0 | 0 | 0 | 0 | 0.0011 | *0.0001* |
| A*29:01 | 0 | 0.0027 | 0 | 0 | 0 | 0.012 | 0.0355 | 0 | 0 | 0.0041 | 0.0118 | *0.0041* |
| A*29:02 | 0.0243 | 0.023 | 0.0159 | 0.0524 | 0.0317 | 0.024 | 0 | 0.0378 | 0.018 | 0.0041 | 0.0237 | *0.0264* |
| ALLELES | AA (n=103) | BE (n=720) | BS (n=126) | GE (n=105) | GR (n=231) | LG (n=125) | LS (n=366) | LU (n=153) | SG (n=111) | SI (n=155) | ZH (n=293) | All (n=2488) |
| A*29:04 | 0 | 0 | 0 | 0 | 0 | 0 | 0 | 0 | 0 | 0.0041 | 0 | *0* |
| A*29:06 | 0 | 0 | 0 | 0 | 0 | 0 | 0 | 0 | 0 | 0.0041 | 0 | *0* |
| A*29:08N | 0 | 0 | 0 | 0 | 0 | 0 | 0 | 0 | 0 | 0.0041 | 0 | *0* |
| A*29:10 | 0 | 0 | 0 | 0 | 0 | 0 | 0 | 0 | 0 | 0.0041 | 0 | *0* |
| A*29:12 | 0 | 0 | 0 | 0 | 0 | 0 | 0 | 0 | 0 | 0.0041 | 0 | *0* |
| A*30:01/24 | 0.0243 | 0.0181 | 0.0238 | 0.0286 | 0.007 | 0.0091 | 0.0102 | 0.0438 | 0.018 | 0.0129 | 0.0126 | *0.0207* |
| A*30:02 | 0 | 0.0101 | 0 | 0.0429 | 0.0008 | 0.0137 | 0.0117 | 0.0049 | 0 | 0.0029 | 0.0209 | *0.0113* |
| A*30:04 | 0 | 0.0079 | 0 | 0.0024 | 0.0012 | 0 | 0 | 0.0037 | 0 | 0.0022 | 0 | *0.0033* |
| A*30:06 | 0 | 0 | 0 | 0.0024 | 0.0012 | 0 | 0 | 0 | 0 | 0.0022 | 0 | *0* |
| A*30:10 | 0 | 0 | 0 | 0 | 0.0008 | 0 | 0 | 0.0049 | 0 | 0.0029 | 0 | *0* |
| A*30:12 | 0 | 0 | 0 | 0 | 0.0008 | 0 | 0 | 0.0049 | 0 | 0.0029 | 0 | *0* |
| A*30:15 | 0 | 0 | 0 | 0 | 0.007 | 0.0091 | 0 | 0 | 0 | 0 | 0.0126 | *0* |
| A*30:18 | 0 | 0 | 0 | 0 | 0.007 | 0 | 0 | 0 | 0 | 0 | 0 | *0* |
| A*30:19 | 0 | 0 | 0 | 0 | 0.007 | 0 | 0 | 0 | 0 | 0 | 0 | *0* |
| A*31:01/14N | 0.0194 | 0.0382 | 0.0675 | 0.0429 | 0.039 | 0.032 | 0.0403 | 0.0176 | 0.0436 | 0.0251 | 0.0427 | *0.0396* |
| A*31:09 | 0 | 0 | 0 | 0 | 0 | 0 | 0 | 0 | 0 | 0.0251 | 0 | *0* |
| A*32:01 | 0.034 | 0.0472 | 0.0296 | 0.0429 | 0.0623 | 0.044 | 0.0493 | 0.0478 | 0.045 | 0.047 | 0.0341 | *0.0459* |
| A*33 | 0.0049 | 0.0125 | 0.013 | 0.0238 | 0.013 | 0.02 | 0.015 | 0.0065 | 0.0135 | 0.0032 | 0.0239 | *0.014* |
| A*34 | 0 | 0.0007 | 0 | 0.0048 | 0 | 0 | 0.0014 | 0 | 0 | 0 | 0.0017 | *0.0008* |
| A*36 | 0 | 0 | 0 | 0 | 0.0022 | 0 | 0 | 0 | 0 | 0.0032 | 0 | *0.0004* |
| A*66:01/04 | 0.0209 | 0.0042 | 0.0079 | 0.0143 | 0.0004 | 0.008 | 0.0041 | 0.0098 | 0.0135 | 0.0032 | 0.0085 | *0.0067* |
| A*66:02 | 0 | 0 | 0 | 0 | 0.0004 | 0 | 0 | 0 | 0 | 0 | 0 | *0* |
| A*66:05 | 0 | 0 | 0 | 0 | 0.0004 | 0 | 0 | 0 | 0 | 0 | 0 | *0* |
| A*66:07 | 0 | 0 | 0 | 0 | 0.0004 | 0 | 0 | 0 | 0 | 0 | 0 | *0* |
| A*66unspecified | 0 | 0 | 0 | 0 | 0.0004 | 0 | 0 | 0 | 0 | 0 | 0 | *0* |
| A*68:01/11N/33 | 0.0439 | 0.035 | 0.0508 | 0.02 | 0.0258 | 0.0466 | 0.0323 | 0.0354 | 0.0416 | 0.0544 | 0.0495 | *0.0383* |
| A*68:02 | 0.022 | 0.0046 | 0.0127 | 0.0133 | 0.0105 | 0.0291 | 0.0072 | 0.0202 | 0.026 | 0.0155 | 0.0165 | *0.0134* |
| A*68:18N | 0 | 0 | 0 | 0 | 0.0105 | 0 | 0 | 0 | 0 | 0 | 0 | *0* |
| A*69:01 | 0.0049 | 0.0014 | 0 | 0.0048 | 0.0022 | 0 | 0.0027 | 0.0098 | 0 | 0 | 0.0017 | *0.0022* |
| ALLELES | AA (n=103) | BE (n=720) | BS (n=126) | GE (n=105) | GR (n=231) | LG (n=125) | LS (n=366) | LU (n=153) | SG (n=111) | SI (n=155) | ZH (n=293) | All (n=2488) |
| A*74 | 0 | 0 | 0 | 0 | 0 | 0 | 0.0027 | 0 | 0 | 0 | 0 | *0.0004* |
| A*80:01 | 0 | 0.0007 | 0.0119 | 0 | 0 | 0.004 | 0 | 0 | 0 | 0 | 0.0034 | *0.0014* |
| blank | 0.0244 | 0 | 0.0167 | 0 | 0.008 | 0.0035 | 0.0221 | 0.0145 | 0.0099 | 0.0174 | 0.004 | *0.0043* |
| Expected heterozygozity | 0.841 | 0.867 | 0.863 | 0.894 | 0.870 | 0.867 | 0.879 | 0.876 | 0.847 | 0.889 | 0.882 | 0.871 |
| HWE p-value | 0.394 | 0.095 | 0.079 | 0.152 | 0.487 | 0.362 | 0.051 | 0.466 | 0.445 | 0.073 | 0.548 | 3.58E-08 |

**HLA-B**

| ALLELES | AA (n=124) | BE (n=777) | BS (n=178) | GE (n=150) | GR (n=246) | LG (n=167) | LS (n=385) | LU (n=199) | SG (n=127) | SI (n=153) | ZH (n=418) | All (n=2924) |
| --- | --- | --- | --- | --- | --- | --- | --- | --- | --- | --- | --- | --- |
| B*07:02/44/49N/58/59/61 | 0.086 | 0.1265 | *0.0766* | 0.0595 | 0.099 | 0.0609 | 0.1027 | 0.1143 | 0.1031 | 0.0797 | *0.0897* | *0.1011* |
| B*07:05/B*07:06 | 0 | 0.0024 | *0.0038* | 0.0132 | 0.0124 | 0.0068 | 0.0164 | 0.0185 | 0.0055 | 0.0053 | *0.0087* | *0.0065* |
| B*07:07 | 0 | 0 | *0* | 0 | 0 | 0.0068 | 0 | 0 | 0 | 0 | *0* | *0.0003* |
| B*07:10 | 0 | 0.0008 | *0* | 0 | 0 | 0 | 0 | 0 | 0 | 0 | *0* | *0.0003* |
| B*07:31 | 0 | 0.0008 | *0* | 0 | 0 | 0 | 0 | 0 | 0 | 0 | *0* | *0.0003* |
| B*07:37 | 0.0072 | 0.0008 | *0* | 0 | 0 | 0 | 0 | 0 | 0 | 0 | *0.0021* | *0.0009* |
| B*08:01/19N | 0.0506 | 0.0811 | *0.0564* | 0.0754 | 0.0706 | 0.0628 | 0.0905 | 0.0571 | 0.0604 | 0.0752 | *0.0525* | *0.0704* |
| B*13:01 | 0 | 0 | *0* | 0 | 0 | 0 | 0 | 0.0043 | 0 | 0 | *0* | *0.0002* |
| B*13:02 | 0.0202 | 0.0302 | *0.0291* | 0.0053 | 0.0346 | 0.0269 | 0.0221 | 0.0258 | 0.0276 | 0.0261 | *0.0227* | *0.0263* |
| B*13:08Q | 0 | 0 | *0* | 0.0053 | 0 | 0 | 0 | 0 | 0 | 0 | *0* | *0* |
| B*14:01 | 0.004 | 0.0071 | *0.0089* | 0.0073 | 0.0101 | 0.003 | 0.0129 | 0.005 | 0.0049 | 0.0108 | *0.0107* | *0.0083* |
| B*14:02 | 0.0081 | 0.0122 | *0.0281* | 0.0293 | 0.0177 | 0.0299 | 0.0157 | 0.0075 | 0.0148 | 0.0252 | *0.0149* | *0.0168* |
| B*14:06 | 0 | 0 | *0* | 0 | 0 | 0 | 0 | 0 | 0 | 0 | *0.0019* | *0.0002* |
| B*15:01/102/104/140/146 | 0.0678 | 0.0603 | *0.0543* | 0.0428 | 0.0473 | 0.0383 | 0.0213 | 0.0437 | 0.0529 | 0.053 | *0.063* | *0.0519* |
| B*15:03/B*15:103 | 0.0064 | 0.0034 | *0* | 0.0202 | 0 | 0.0115 | 0.003 | 0.0049 | 0.0096 | 0.0193 | *0.0057* | *0.0061* |
| B*15:08 | 0 | 0 | *0* | 0.0025 | 0 | 0 | 0 | 0.0097 | 0.0048 | 0 | *0.0019* | *0.0013* |
| B*15:09 | 0 | 0 | *0* | 0 | 0.0118 | 0 | 0 | 0 | 0 | 0 | *0* | *0.0006* |
| B*15:10 | 0 | 0 | *0* | 0 | 0.003 | 0 | 0 | 0 | 0 | 0 | *0* | *0.0003* |
| B*15:11 | 0 | 0 | *0* | 0 | 0 | 0 | 0 | 0 | 0 | 0 | *0.0019* | *0.0003* |
| B*15:15 | 0 | 0 | *0* | 0.0025 | 0 | 0 | 0 | 0 | 0 | 0 | *0* | *0* |
| B*15:16 | 0 | 0.0004 | *0* | 0 | 0 | 0 | 0.0017 | 0.0049 | 0 | 0 | *0* | *0.0008* |
| B*15:17 | 0.0064 | 0.007 | *0.0319* | 0.0101 | 0 | 0.0077 | 0.024 | 0 | 0.0096 | 0.0144 | *0.0019* | *0.0091* |
| B*15:18 | 0 | 0.0043 | *0.0046* | 0.0025 | 0 | 0.0038 | 0.0015 | 0.0032 | 0.0048 | 0.0016 | *0.0057* | *0.0048* |
| B*15:20 | 0 | 0 | *0.0025* | 0 | 0 | 0 | 0 | 0 | 0 | 0 | *0* | *0.0003* |
| B*15:24 | 0 | 0.0008 | *0* | 0.0051 | 0 | 0 | 0.0061 | 0 | 0.0048 | 0 | *0* | *0.0013* |
| B*15:25 | 0 | 0 | *0.0025* | 0 | 0 | 0 | 0 | 0 | 0 | 0 | *0* | *0* |
| B*15:29 | 0 | 0 | *0* | 0 | 0 | 0 | 0 | 0 | 0 | 0 | *0.0019* | *0.0003* |
| ALLELES | AA (n=124) | BE (n=777) | BS (n=178) | GE (n=150) | GR (n=246) | LG (n=167) | LS (n=385) | LU (n=199) | SG (n=127) | SI (n=153) | ZH (n=418) | All (n=2924) |
| B*15:39 | 0 | 0 | *0* | 0 | 0 | 0.0038 | 0 | 0 | 0 | 0 | *0* | *0.0003* |
| B*15:67 | 0 | 0.0004 | *0* | 0 | 0 | 0 | 0.0017 | 0 | 0 | 0 | *0* | *0* |
| B*15:72 | 0 | 0 | *0.0046* | 0.0025 | 0 | 0.0038 | 0.0015 | 0.0032 | 0 | 0.0016 | *0* | *0* |
| B*15:80 | 0 | 0 | *0* | 0 | 0 | 0 | 0 | 0.0032 | 0 | 0.0016 | *0* | *0* |
| B*15:90 | 0 | 0 | *0* | 0 | 0.003 | 0 | 0 | 0 | 0 | 0 | *0* | *0* |
| B*18:01/17N | 0.0363 | 0.056 | *0.0406* | 0.0381 | 0.0407 | 0.0619 | 0.0442 | 0.0402 | 0.0236 | 0.0359 | *0.0311* | *0.0437* |
| B*18:04 | 0 | 0 | *0* | 0 | 0 | 0.0039 | 0 | 0 | 0 | 0 | *0* | *0.0002* |
| B*27:02 | 0 | 0.0064 | *0.0034* | 0.0017 | 0.0041 | 0.0072 | 0.0054 | 0.0037 | 0.0063 | 0.0071 | *0.0033* | *0.005* |
| B*27:03 | 0 | 0 | *0* | 0.0033 | 0 | 0 | 0 | 0 | 0 | 0 | *0* | *0* |
| B*27:05/13 | 0.0161 | 0.0334 | *0.0189* | 0.0033 | 0.034 | 0.0072 | 0.0356 | 0.0258 | 0.0418 | 0.0321 | *0.0169* | *0.0267* |
| B*27:07 | 0 | 0 | *0.0032* | 0 | 0 | 0.0036 | 0 | 0 | 0 | 0 | *0.0018* | *0.0006* |
| B*27:12 | 0 | 0 | *0* | 0.0033 | 0 | 0 | 0 | 0 | 0 | 0 | *0* | *0.0002* |
| B*27:14 | 0 | 0.0007 | *0* | 0 | 0 | 0 | 0 | 0 | 0 | 0 | *0* | *0.0002* |
| B*27:17 | 0 | 0 | *0* | 0.0033 | 0 | 0 | 0 | 0 | 0 | 0 | *0* | *0* |
| B*27:30 | 0 | 0 | *0* | 0.0017 | 0 | 0 | 0 | 0 | 0 | 0 | *0* | *0* |
| B*35:01/40N/42/57/94 | 0.0364 | 0.0725 | *0.0677* | 0.038 | 0.0255 | 0.0663 | 0.0423 | 0.0685 | 0.0224 | 0.0454 | *0.0532* | *0.0564* |
| B*35:02 | 0 | 0.0074 | *0.0182* | 0.0345 | 0.0085 | 0.0048 | 0.0102 | 0.0194 | 0.0168 | 0.0114 | *0.0162* | *0.0115* |
| B*35:03/70 | 0.0552 | 0.0222 | *0.0292* | 0.0253 | 0.034 | 0.036 | 0.0348 | 0.0145 | 0.0448 | 0.0454 | *0.0452* | *0.0327* |
| B*35:04 | 0 | 0 | *0.0036* | 0 | 0 | 0 | 0 | 0 | 0 | 0 | *0* | *0.0005* |
| B*35:06 | 0 | 0 | *0* | 0 | 0 | 0.008 | 0 | 0 | 0 | 0 | *0* | *0* |
| B*35:08 | 0.0052 | 0.0015 | *0.0036* | 0 | 0.0128 | 0.0103 | 0.0075 | 0.0105 | 0.0056 | 0.0057 | *0.0045* | *0.0047* |
| B*35:36 | 0 | 0 | *0* | 0 | 0 | 0 | 0 | 0.0145 | 0 | 0 | *0* | *0* |
| B*37:01 | 0.0088 | 0.0103 | *0.0112* | 0.0067 | 0.0129 | 0.006 | 0.0104 | 0.0176 | 0.0157 | 0.0065 | *0.0108* | *0.0121* |
| B*37:02 | 0 | 0 | *0* | 0 | 0.002 | 0 | 0 | 0 | 0.0039 | 0 | *0* | *0.0003* |
| B*37:03N | 0 | 0 | *0* | 0 | 0.0129 | 0 | 0 | 0 | 0 | 0.0065 | *0* | *0* |
| B*37:04 | 0 | 0 | *0* | 0.0033 | 0 | 0 | 0 | 0 | 0 | 0 | *0* | *0.0002* |
| B*38:01 | 0.0242 | 0.0142 | *0.0361* | 0.0267 | 0.0163 | 0.0299 | 0.0244 | 0.0088 | 0.0276 | 0.0131 | *0.0191* | *0.0212* |
| B*38:09 | 0 | 0 | *0* | 0 | 0 | 0 | 0 | 0.0088 | 0 | 0.0131 | *0* | *0* |
| B*39:01 | 0.0101 | 0.0086 | *0.018* | 0.0012 | 0.002 | 0.0144 | 0.017 | 0.0057 | 0.0177 | 0.0013 | *0.0116* | *0.0117* |
| ALLELES | AA (n=124) | BE (n=777) | BS (n=178) | GE (n=150) | GR (n=246) | LG (n=167) | LS (n=385) | LU (n=199) | SG (n=127) | SI (n=153) | ZH (n=418) | All (n=2924) |
| B*39:01L | 0 | 0 | *0* | 0.0012 | 0.002 | 0 | 0 | 0 | 0 | 0.0013 | *0* | *0* |
| B*39:04 | 0 | 0 | *0* | 0.0012 | 0 | 0 | 0 | 0 | 0 | 0 | *0* | *0* |
| B*39:06 | 0.0101 | 0.0055 | *0.0045* | 0.0163 | 0.0054 | 0.0012 | 0.0038 | 0.0075 | 0.0118 | 0.0065 | *0.0077* | *0.0066* |
| B*39:09 | 0 | 0.0007 | *0* | 0 | 0 | 0 | 0 | 0 | 0 | 0 | *0* | *0.0002* |
| B*39:10 | 0 | 0 | *0* | 0 | 0.002 | 0 | 0 | 0 | 0 | 0 | *0* | *0.0002* |
| B*39:12 | 0 | 0 | *0* | 0.0012 | 0 | 0 | 0 | 0 | 0 | 0 | *0* | *0* |
| B*39:24 | 0 | 0 | *0* | 0 | 0.0014 | 0.0012 | 0 | 0.0019 | 0.003 | 0 | *0.0019* | *0.0006* |
| B*39:25N | 0 | 0 | *0* | 0.0012 | 0.002 | 0 | 0 | 0 | 0 | 0.0013 | *0* | *0* |
| B*39:26 | 0 | 0 | *0* | 0.0012 | 0 | 0 | 0 | 0 | 0 | 0.0013 | *0* | *0* |
| B*39:27 | 0 | 0 | *0* | 0.0012 | 0.002 | 0 | 0 | 0.0057 | 0 | 0.0013 | *0* | *0* |
| B*39:28 | 0 | 0 | *0* | 0 | 0.0014 | 0.0012 | 0 | 0.0019 | 0.003 | 0 | *0.0019* | *0.0006* |
| B*40:01/55 | 0.0352 | 0.049 | *0.0531* | 0.0278 | 0.0423 | 0.0135 | 0.0417 | 0.0226 | 0.0336 | 0.055 | *0.0524* | *0.0425* |
| B*40:02/56 | 0.0132 | 0.0151 | *0.0088* | 0.0087 | 0.0203 | 0.0067 | 0.0179 | 0.0075 | 0.0118 | 0.0127 | *0.0159* | *0.0143* |
| B*40:04 | 0 | 0 | *0* | 0.0022 | 0 | 0 | 0 | 0 | 0 | 0 | *0* | *0.0002* |
| B*40:06 | 0 | 0.0022 | *0* | 0 | 0 | 0 | 0.0007 | 0.0025 | 0 | 0.0021 | *0.0064* | *0.0022* |
| B*40:11 | 0 | 0 | *0* | 0.0022 | 0 | 0 | 0 | 0 | 0 | 0 | *0* | *0* |
| B*40:35 | 0 | 0 | *0* | 0 | 0 | 0.0067 | 0 | 0 | 0 | 0 | *0* | *0* |
| B*40:53 | 0 | 0 | *0* | 0 | 0 | 0 | 0.0007 | 0.0025 | 0 | 0 | *0* | *0* |
| B*40:70 | 0 | 0 | *0* | 0 | 0 | 0 | 0 | 0 | 0 | 0.0021 | *0* | *0* |
| B*41:01 | 0.0188 | 0.0026 | *0.0099* | 0.0067 | 0.0007 | 0.003 | 0.0026 | 0.003 | 0 | 0 | *0.0108* | *0.005* |
| B*41:02 | 0.0094 | 0.0045 | *0.0041* | 0.0133 | 0.002 | 0 | 0 | 0.009 | 0.0118 | 0.0033 | *0.0036* | *0.0047* |
| B*41:06 | 0 | 0 | *0* | 0 | 0.0007 | 0 | 0 | 0 | 0 | 0 | *0* | *0* |
| B*41:07 | 0 | 0 | *0* | 0 | 0.0007 | 0.003 | 0 | 0.003 | 0 | 0 | *0* | *0* |
| B*42 | 0 | 0 | *0* | 0.0067 | 0 | 0 | 0 | 0 | 0 | 0 | *0* | *0.0003* |
| B*44:02/19N/27 | 0.0575 | 0.0744 | *0.053* | 0.0515 | 0.0803 | 0.0432 | 0.0594 | 0.0403 | 0.0725 | 0.0669 | *0.0604* | *0.0638* |
| B*44:03 | 0.0611 | 0.0476 | *0.0368* | 0.0562 | 0.0463 | 0.0346 | 0.0674 | 0.0686 | 0.0278 | 0.0531 | *0.0504* | *0.0502* |
| B*44:04 | 0 | 0.0008 | *0* | 0 | 0 | 0 | 0 | 0 | 0 | 0.0035 | *0* | *0.0004* |
| B*44:05 | 0.0121 | 0.0052 | *0.0038* | 0.0047 | 0 | 0 | 0 | 0.0017 | 0.0049 | 0.002 | *0.0034* | *0.004* |
| B*44:06 | 0 | 0.0007 | *0* | 0 | 0 | 0 | 0 | 0 | 0 | 0 | *0* | *0.0002* |
| ALLELES | AA (n=124) | BE (n=777) | BS (n=178) | GE (n=150) | GR (n=246) | LG (n=167) | LS (n=385) | LU (n=199) | SG (n=127) | SI (n=153) | ZH (n=418) | All (n=2924) |
| B*44:14 | 0 | 0 | *0* | 0 | 0.0047 | 0 | 0 | 0.0017 | 0 | 0.002 | *0* | *0* |
| B*44:17 | 0 | 0 | *0* | 0 | 0.0023 | 0 | 0 | 0 | 0 | 0 | *0* | *0.0002* |
| B*45:01/07 | 0 | 0.0019 | *0.0028* | 0.0033 | 0.002 | 0.006 | 0.0045 | 0 | 0 | 0.0065 | *0.0012* | *0.0039* |
| B*45:03 | 0 | 0 | *0.0028* | 0.0033 | 0.002 | 0 | 0.0045 | 0 | 0 | 0.0065 | *0* | *0* |
| B*46:01 | 0.004 | 0.0006 | *0* | 0 | 0.001 | 0 | 0.0006 | 0 | 0 | 0 | *0* | *0.0007* |
| B*46:02 | 0 | 0 | *0* | 0 | 0.001 | 0 | 0.0006 | 0 | 0 | 0 | *0* | *0* |
| B*47:01 | 0.004 | 0.0045 | *0.0028* | 0.0033 | 0.0061 | 0 | 0.0039 | 0 | 0.0039 | 0.0131 | *0.0132* | *0.0055* |
| B*48:01/09 | 0 | 0.0003 | *0.0028* | 0 | 0 | 0 | 0 | 0.0013 | 0 | 0.0016 | *0* | *0.0007* |
| B*48:11 | 0 | 0.0003 | *0* | 0 | 0 | 0 | 0 | 0.0013 | 0 | 0.0016 | *0* | *0* |
| B*49:01 | 0.0161 | 0.0212 | *0.0084* | 0.03 | 0.0122 | 0.021 | 0.0234 | 0.0327 | 0.0118 | 0.0065 | *0.0144* | *0.0188* |
| B*50:01 | 0.0202 | 0.009 | *0.0105* | 0.0133 | 0.0163 | 0.024 | 0.0182 | 0.0209 | 0.0118 | 0.0025 | *0.0144* | *0.0139* |
| B*50:02 | 0 | 0.0026 | *0.0035* | 0 | 0.0041 | 0 | 0 | 0.0042 | 0 | 0.0049 | *0* | *0.0018* |
| B*50:04 | 0 | 0 | *0* | 0 | 0 | 0 | 0 | 0 | 0 | 0.0025 | *0* | *0* |
| B*51:01/11N/30/32/48/51 | 0.09 | 0.0779 | *0.0461* | 0.0477 | 0.1009 | 0.1383 | 0.0661 | 0.0676 | 0.0688 | 0.0967 | *0.0709* | *0.0773* |
| B*51:02 | 0 | 0 | *0* | 0 | 0 | 0 | 0 | 0 | 0 | 0 | *0.0023* | *0.0003* |
| B*51:05 | 0 | 0.0026 | *0* | 0 | 0 | 0.0189 | 0 | 0 | 0 | 0.0048 | *0.002* | *0.0024* |
| B*51:07 | 0 | 0 | *0.0031* | 0 | 0 | 0 | 0.0033 | 0 | 0 | 0.0145 | *0.0021* | *0.0018* |
| B*51:08 | 0.0129 | 0.0044 | *0.0031* | 0.004 | 0 | 0.0031 | 0.0099 | 0 | 0.0071 | 0 | *0.004* | *0.0048* |
| B*51:20 | 0 | 0 | *0* | 0 | 0 | 0.0031 | 0 | 0 | 0 | 0 | *0* | *0* |
| B*52:01/07 | 0.0302 | 0.0122 | *0.018* | 0.027 | 0.0303 | 0.0247 | 0.0165 | 0.0056 | 0.0284 | 0.0024 | *0.0181* | *0.0178* |
| B*52:04 | 0 | 0 | *0* | 0 | 0 | 0 | 0 | 0.0056 | 0 | 0 | *0* | *0* |
| B*52:05 | 0 | 0 | *0* | 0 | 0 | 0 | 0 | 0 | 0 | 0.0024 | *0* | *0* |
| B*53:01 | 0.0242 | 0.0142 | *0.0197* | 0.0433 | 0.0081 | 0.018 | 0.0052 | 0.0063 | 0.0236 | 0.0082 | *0.0257* | *0.0176* |
| B*53:10 | 0 | 0 | *0* | 0 | 0.0081 | 0 | 0 | 0.0063 | 0 | 0.0082 | *0* | *0* |
| B*55:01 | 0.0161 | 0.0141 | *0.0281* | 0.0133 | 0.0183 | 0.015 | 0.0104 | 0.0276 | 0.0315 | 0.0076 | *0.02* | *0.0206* |
| B*55:02 | 0 | 0.0004 | *0* | 0 | 0 | 0 | 0 | 0 | 0 | 0 | *0* | *0.0001* |
| B*55:03 | 0 | 0 | *0* | 0.0133 | 0 | 0 | 0.0104 | 0 | 0 | 0.0076 | *0* | *0* |
| B*55:12 | 0 | 0.0004 | *0* | 0 | 0 | 0 | 0 | 0 | 0 | 0 | *0* | *0.0001* |
| B*55:15 | 0 | 0 | *0* | 0.0133 | 0 | 0 | 0 | 0 | 0 | 0.0076 | *0* | *0* |
| ALLELES | AA (n=124) | BE (n=777) | BS (n=178) | GE (n=150) | GR (n=246) | LG (n=167) | LS (n=385) | LU (n=199) | SG (n=127) | SI (n=153) | ZH (n=418) | All (n=2924) |
| B*56:01 | 0.0161 | 0.0077 | *0.0056* | 0.0003 | 0.002 | 0.009 | 0.0099 | 0.0075 | 0.0079 | 0.0065 | *0.0077* | *0.0077* |
| B*56:02 | 0 | 0 | *0* | 0.0003 | 0 | 0 | 0 | 0 | 0 | 0 | *0* | *0* |
| B*56:04 | 0 | 0 | *0* | 0.0003 | 0 | 0 | 0 | 0 | 0 | 0 | *0* | *0* |
| B*56:05 | 0 | 0 | *0* | 0.0003 | 0 | 0 | 0 | 0 | 0 | 0 | *0* | *0* |
| B*56:15 | 0 | 0 | *0* | 0.0003 | 0 | 0 | 0 | 0 | 0 | 0 | *0* | *0* |
| B*56:16 | 0 | 0 | *0* | 0.0003 | 0 | 0 | 0 | 0 | 0 | 0 | *0* | *0* |
| B*56:17 | 0 | 0 | *0* | 0.0003 | 0 | 0 | 0 | 0 | 0 | 0 | *0* | *0* |
| B*56:19N | 0 | 0 | *0* | 0.0003 | 0 | 0 | 0 | 0 | 0 | 0 | *0* | *0* |
| B*56:20 | 0 | 0 | *0* | 0.0003 | 0.002 | 0 | 0 | 0 | 0 | 0 | *0* | *0* |
| B*56unspecified | 0 | 0 | *0* | 0.0003 | 0 | 0 | 0 | 0 | 0 | 0 | *0* | *0* |
| B*57:01 | 0.0628 | 0.0323 | *0.0449* | 0.0433 | 0.0291 | 0.0467 | 0.0434 | 0.0546 | 0.0657 | 0.0359 | *0.0459* | *0.0418* |
| B*57:02 | 0 | 0.0007 | *0* | 0 | 0.0069 | 0 | 0 | 0 | 0 | 0 | *0.0013* | *0.001* |
| B*57:03 | 0.0051 | 0.0023 | *0* | 0 | 0 | 0.0072 | 0.0045 | 0 | 0 | 0 | *0.0043* | *0.0026* |
| B*58:01/11 | 0.0081 | 0.0103 | *0.0421* | 0.0326 | 0.0122 | 0.024 | 0.0143 | 0.0226 | 0.0157 | 0 | *0.0207* | *0.0168* |
| B*58:02 | 0 | 0 | *0* | 0.0041 | 0 | 0 | 0 | 0 | 0 | 0 | *0.0013* | *0.0004* |
| B*73:01 | 0.004 | 0 | *0* | 0 | 0 | 0 | 0 | 0.0025 | 0 | 0 | *0.0012* | *0.0005* |
| blank | 0.0199 | 0 | *0.0249* | 0.024 | 0.0057 | 0.0013 | 0.0032 | 0.0104 | 0.0144 | 0 | *0.0157* | *0.0079* |
| Expected heterozygozity | 0.957 | 0.944 | 0.963 | 0.968 | 0.954 | 0.953 | 0.955 | 0.957 | 0.959 | 0.957 | 0.959 | 0.955 |
| HWE p-value | 0.056 | 0.046 | 0.0023 | 0.353 | 0.955 | 0.135 | 0.673 | 0.147 | 0.818 | 0.254 | 0.0013 | 1.36E-10 |

**HLA-C**

| ALLELES | AA (n=291) | BE (n=751) | BS (n=341) | GE (n=200) | GR (n=209) | LG (n=218) | LS (n=154) | LU (n=292) | SG (n=316) | SI (n=101) | ZH (n=639) | All (n=3512) |
| --- | --- | --- | --- | --- | --- | --- | --- | --- | --- | --- | --- | --- |
| C*01:02 | 0.0447 | 0.0366 | 0.0191 | 0.045 | 0.0478 | 0.0367 | 0.039 | 0.0342 | 0.0427 | 0.0396 | 0.036 | 0.0372 |
| C*02:02 | 0.0395 | 0.0443 | 0.0557 | 0.045 | 0.0598 | 0.0436 | 0.0506 | 0.0445 | 0.0491 | 0.0594 | 0.0407 | 0.0462 |
| C*02:10 | 0 | 0.0009 | 0 | 0 | 0 | 0 | 0 | 0 | 0 | 0 | 0 | 0.0005 |
| C*03:02 | 0.0054 | 0.0066 | 0.0072 | 0 | 0 | 0 | 0 | 0.0094 | 0.0091 | 0 | 0.0081 | 0.0066 |
| C*03:03/20N | 0.047 | 0.062 | 0.0617 | 0.0507 | 0.0469 | 0.034 | 0.051 | 0.0272 | 0.0562 | 0.0627 | 0.0482 | 0.0514 |
| C*03:04 | 0.073 | 0.0539 | 0.0631 | 0.0498 | 0.0077 | 0.0297 | 0.0417 | 0.0472 | 0.0708 | 0.0314 | 0.0681 | 0.0566 |
| C*03:05 | 0 | 0 | 0 | 0.0045 | 0 | 0.0074 | 0.0015 | 0 | 0 | 0 | 0 | 0 |
| C*03:06 | 0 | 0 | 0 | 0 | 0.0077 | 0 | 0 | 0 | 0 | 0 | 0 | 0 |
| C*03:09 | 0 | 0 | 0 | 0 | 0.0077 | 0 | 0 | 0 | 0 | 0 | 0 | 0 |
| C*03:19 | 0 | 0 | 0 | 0 | 0.0077 | 0 | 0 | 0 | 0 | 0 | 0 | 0 |
| C*03:23 | 0 | 0 | 0 | 0 | 0.0077 | 0 | 0 | 0 | 0 | 0 | 0 | 0 |
| C*03:24 | 0 | 0 | 0 | 0 | 0.0077 | 0 | 0 | 0 | 0 | 0 | 0 | 0 |
| C*03:25 | 0 | 0 | 0 | 0 | 0 | 0 | 0.0015 | 0 | 0 | 0 | 0 | 0 |
| C*03:26 | 0 | 0 | 0 | 0 | 0.0077 | 0 | 0 | 0 | 0 | 0 | 0 | 0 |
| C*03:27 | 0 | 0 | 0 | 0 | 0 | 0 | 0.0015 | 0 | 0 | 0 | 0 | 0 |
| C*04:01/09N/28/30 | 0.1564 | 0.1345 | 0.1389 | 0.105 | 0.122 | 0.1376 | 0.129 | 0.1467 | 0.1076 | 0.1485 | 0.1377 | 0.1339 |
| C*04:03 | 0 | 0 | 0.0019 | 0 | 0 | 0 | 0 | 0.004 | 0 | 0 | 0 | 0.0005 |
| C*05:01/03 | 0.0756 | 0.0888 | 0.0733 | 0.1025 | 0.067 | 0.0734 | 0.0798 | 0.0942 | 0.0934 | 0.0693 | 0.0869 | 0.0843 |
| C*05:14 | 0 | 0.0017 | 0 | 0 | 0 | 0 | 0 | 0 | 0 | 0 | 0 | 0.0004 |
| C*06:02 | 0.0893 | 0.0779 | 0.0806 | 0.075 | 0.1172 | 0.0872 | 0.084 | 0.0959 | 0.106 | 0.1188 | 0.0822 | 0.0881 |
| C*07:01/06/18/52 | 0.1312 | 0.1438 | 0.1431 | 0.1225 | 0.1308 | 0.1524 | 0.1674 | 0.1383 | 0.123 | 0.1434 | 0.1293 | 0.1373 |
| C*07:02/50 | 0.1232 | 0.135 | 0.1073 | 0.1162 | 0.142 | 0.0715 | 0.1151 | 0.1107 | 0.1152 | 0.0978 | 0.1245 | 0.1194 |
| C*07:04/11 | 0.0222 | 0.014 | 0.0179 | 0.0157 | 0.0139 | 0.0268 | 0.0077 | 0.018 | 0.0149 | 0.0261 | 0.0187 | 0.0171 |
| C*07:07 | 0 | 0 | 0 | 0 | 0.0028 | 0 | 0 | 0 | 0 | 0 | 0 | 0.0004 |
| C*07:09 | 0 | 0 | 0 | 0 | 0 | 0 | 0 | 0 | 0 | 0 | 0.0013 | 0 |
| C*07:16 | 0 | 0.0008 | 0 | 0 | 0 | 0 | 0 | 0 | 0 | 0 | 0 | 0.0002 |
| C*07:22 | 0 | 0 | 0 | 0.0031 | 0 | 0 | 0 | 0 | 0 | 0 | 0 | 0.0002 |
| ALLELES | AA (n=291) | BE (n=751) | BS (n=341) | GE (n=200) | GR (n=209) | LG (n=218) | LS (n=154) | LU (n=292) | SG (n=316) | SI (n=101) | ZH (n=639) | All (n=3512) |
| C*07:24 | 0 | 0 | 0 | 0 | 0 | 0.0085 | 0 | 0 | 0 | 0 | 0 | 0.0005 |
| C*07:29 | 0 | 0 | 0 | 0 | 0 | 0 | 0 | 0.0036 | 0 | 0 | 0 | 0.0003 |
| C*08:01 | 0 | 0.0004 | 0 | 0 | 0 | 0 | 0 | 0 | 0 | 0.005 | 0 | 0.0003 |
| C*08:02 | 0.0241 | 0.0206 | 0.023 | 0.05 | 0.0311 | 0.0367 | 0.0162 | 0.0254 | 0.0237 | 0.0347 | 0.0219 | 0.0255 |
| C*08:03 | 0 | 0 | 0.0019 | 0 | 0 | 0 | 0 | 0.002 | 0 | 0 | 0 | 0.0003 |
| C*08:08 | 0 | 0.0004 | 0 | 0 | 0 | 0 | 0 | 0 | 0 | 0 | 0 | 0 |
| C*12:02 | 0.0155 | 0.0074 | 0.0148 | 0.017 | 0.0073 | 0.0037 | 0.0123 | 0.009 | 0.0152 | 0 | 0.0178 | 0.0119 |
| C*12:03 | 0.0412 | 0.0479 | 0.0745 | 0.078 | 0.0477 | 0.0951 | 0.0554 | 0.0493 | 0.0481 | 0.0446 | 0.0482 | 0.0551 |
| C*12:04 | 0 | 0 | 0.0046 | 0 | 0 | 0 | 0 | 0 | 0 | 0 | 0 | 0.0004 |
| C*12:07 | 0 | 0 | 0 | 0 | 0 | 0.0044 | 0 | 0 | 0 | 0 | 0 | 0.0003 |
| C*12:13 | 0 | 0 | 0 | 0 | 0 | 0 | 0 | 0 | 0 | 0 | 0.0028 | 0.0004 |
| C*14:02 | 0.0223 | 0.0192 | 0.0213 | 0.0067 | 0.0287 | 0.0459 | 0.0182 | 0.024 | 0.0206 | 0.0033 | 0.0209 | 0.0218 |
| C*14:03 | 0 | 0.0008 | 0.0021 | 0.0033 | 0 | 0 | 0.0045 | 0 | 0 | 0 | 0.001 | 0.0009 |
| C*14:07N | 0 | 0 | 0 | 0 | 0 | 0 | 0 | 0 | 0 | 0.0033 | 0 | 0 |
| C*14:08 | 0 | 0 | 0 | 0 | 0 | 0 | 0 | 0 | 0 | 0.0033 | 0 | 0 |
| C*15:02/13 | 0.0464 | 0.0492 | 0.037 | 0.0391 | 0.0122 | 0.0447 | 0.0406 | 0.0414 | 0.0549 | 0.0535 | 0.0497 | 0.0452 |
| C*15:04 | 0 | 0 | 0 | 0 | 0 | 0.0061 | 0 | 0 | 0 | 0 | 0 | 0.0003 |
| C*15:05 | 0 | 0.0028 | 0.0028 | 0.0084 | 0 | 0 | 0.0081 | 0.0028 | 0.0017 | 0.002 | 0.0075 | 0.0038 |
| C*15:06 | 0 | 0.0019 | 0.0028 | 0 | 0.0091 | 0 | 0 | 0.0028 | 0.0017 | 0.002 | 0 | 0.0019 |
| C*15:07 | 0 | 0 | 0 | 0 | 0 | 0.0065 | 0 | 0 | 0 | 0 | 0 | 0.0004 |
| C*15:09 | 0 | 0 | 0 | 0 | 0 | 0 | 0 | 0.0028 | 0.0017 | 0.002 | 0 | 0 |
| C*15:10 | 0 | 0 | 0 | 0 | 0.0122 | 0 | 0 | 0 | 0 | 0 | 0 | 0 |
| C*16:01 | 0.0298 | 0.0349 | 0.0328 | 0.0344 | 0.0331 | 0.0296 | 0.0536 | 0.0539 | 0.0302 | 0.0371 | 0.0303 | 0.0349 |
| C*16:02 | 0.0023 | 0.0056 | 0.0039 | 0.0156 | 0.0099 | 0.0066 | 0.0146 | 0 | 0.0076 | 0.0074 | 0.0082 | 0.0068 |
| C*16:04 | 0.004 | 0.0021 | 0.0044 | 0 | 0 | 0.0052 | 0 | 0.0043 | 0.0018 | 0 | 0.0061 | 0.0032 |
| C*17 | 0.0069 | 0.006 | 0.0044 | 0.0125 | 0.0024 | 0.0069 | 0 | 0.0086 | 0.0047 | 0.005 | 0.0039 | 0.0056 |
| C*18 | 0 | 0 | 0 | 0 | 0.0024 | 0 | 0 | 0 | 0 | 0 | 0 | 0.0001 |
| blank | 0 | 0 | 0 | 0 | 0 | 0 | 0.0066 | 0 | 0 | 0 | 0 | 0 |
| Expected heterozygozity | 0.913 | 0.912 | 0.917 | 0.925 | 0.917 | 0.920 | 0.914 | 0.916 | 0.921 | 0.915 | 0.918 | 0.917 |
| ALLELES | AA (n=291) | BE (n=751) | BS (n=341) | GE (n=200) | GR (n=209) | LG (n=218) | LS (n=154) | LU (n=292) | SG (n=316) | SI (n=101) | ZH (n=639) | All (n=3512) |
| HWE p-value | 1 | 1 | 0.604 | 0.793 | 1 | 1 | 0.558 | 1 | 1 | 1 | 0.709 | 0.909 |

**HLA-DRB1**

| ALLELES | AA (n=1838) | BE (n=3545) | BS (n=1888) | CF (n=408) | FR (n=367) | GE (n=1267) | GR (n=759) | LG (n=1169) | LS (n=993) | LU (n=1553) | SG (n=2113) | SI (n=832) | ZH (n=4875) | All (n=21607) |
| --- | --- | --- | --- | --- | --- | --- | --- | --- | --- | --- | --- | --- | --- | --- |
| DRB1*01:01 | 0.0777 | 0.0852 | 0.0896 | 0.0815 | 0.0704 | 0.075 | 0.0773 | 0.0655 | 0.0661 | 0.0854 | 0.0851 | 0.0754 | *0.0819* | *0.0808* |
| DRB1*01:02 | 0.018 | 0.0119 | 0.015 | 0.0089 | 0.0125 | 0.0232 | 0.0076 | 0.0245 | 0.0176 | 0.0096 | 0.0147 | 0.0154 | *0.0134* | *0.0146* |
| DRB1*01:03 | 0.0055 | 0.0057 | 0.0052 | 0 | 0.0096 | 0.0067 | 0.0016 | 0.0034 | 0.0059 | 0.01 | 0.0068 | 0.0096 | *0.0064* | *0.0061* |
| DRB1*03 | 0.0915 | 0.0965 | 0.089 | 0.099 | 0.0885 | 0.1029 | 0.1012 | 0.0956 | 0.1074 | 0.1069 | 0.0903 | 0.1112 | *0.0896* | *0.0955* |
| DRB1*04 | 0.127 | 0.129 | 0.135 | 0.1295 | 0.1403 | 0.1178 | 0.1373 | 0.0879 | 0.1375 | 0.1362 | 0.1239 | 0.1206 | *0.1296* | *0.1272* |
| DRB1*07 | 0.1357 | 0.1317 | 0.1348 | 0.1516 | 0.1376 | 0.1277 | 0.1241 | 0.1312 | 0.1339 | 0.1323 | 0.1416 | 0.1203 | *0.1369* | *0.1341* |
| DRB1*08 | 0.0386 | 0.0351 | 0.0346 | 0.039 | 0.0272 | 0.0392 | 0.0421 | 0.041 | 0.0335 | 0.0435 | 0.036 | 0.0392 | *0.0381* | *0.0376* |
| DRB1*09:01 | 0.0073 | 0.0085 | 0.0069 | 0.011 | 0.0041 | 0.0059 | 0.0059 | 0.0051 | 0.0121 | 0.008 | 0.0073 | 0.0156 | *0.0068* | *0.0077* |
| DRB1*10:01 | 0.0082 | 0.0072 | 0.0082 | 0.0074 | 0.0095 | 0.0107 | 0.0066 | 0.0184 | 0.0106 | 0.0077 | 0.0059 | 0.0138 | *0.0083* | *0.0088* |
| DRB1*11:01 | 0.0692 | 0.0773 | 0.0621 | 0.049 | 0.0785 | 0.0866 | 0.088 | 0.1059 | 0.0786 | 0.0627 | 0.0805 | 0.1021 | *0.072* | *0.0764* |
| DRB1*11:02 | 0.0066 | 0.0012 | 0.0083 | 0 | 0.0095 | 0.008 | 0.0044 | 0.0112 | 0.0071 | 0.0046 | 0.0055 | 0.002 | *0.0054* | *0.0055* |
| DRB1*11:03 | 0.0114 | 0.0108 | 0.0062 | 0.0151 | 0.0024 | 0.0139 | 0.0155 | 0.0133 | 0.0082 | 0.012 | 0.0095 | 0.0138 | *0.0122* | *0.011* |
| DRB1*11:04 | 0.0497 | 0.0416 | 0.0614 | 0.0815 | 0.0343 | 0.0469 | 0.0518 | 0.0599 | 0.0403 | 0.0432 | 0.0354 | 0.0463 | *0.0448* | *0.0465* |
| DRB1*11:05 | 0 | 0 | 0 | 0 | 0 | 0 | 0 | 0 | 0 | 0 | 0.0004 | 0 | *0.0002* | *0.0001* |
| DRB1*11:07 | 0 | 0 | 0 | 0.002 | 0 | 0 | 0 | 0 | 0 | 0 | 0 | 0 | *0* | *0* |
| DRB1*11:09 | 0 | 0 | 0 | 0 | 0 | 0 | 0 | 0 | 0 | 0.0005 | 0 | 0 | *0.0005* | *0.0001* |
| DRB1*11:11 | 0 | 0 | 0.0004 | 0 | 0 | 0 | 0 | 0 | 0 | 0 | 0 | 0 | *0* | *0* |
| DRB1*11:13 | 0 | 0 | 0 | 0 | 0.002 | 0 | 0 | 0 | 0 | 0 | 0 | 0 | *0* | *0* |
| DRB1*11:14 | 0 | 0 | 0 | 0 | 0 | 0 | 0 | 0.0006 | 0 | 0 | 0 | 0 | *0* | *0* |
| DRB1*11:15 | 0.0005 | 0 | 0 | 0 | 0 | 0.0005 | 0 | 0 | 0 | 0 | 0 | 0 | *0.0002* | *0.0001* |
| DRB1*11:22 | 0 | 0.0002 | 0 | 0 | 0 | 0 | 0 | 0 | 0 | 0 | 0 | 0 | *0* | *0* |
| DRB1*12 | 0.018 | 0.0159 | 0.0188 | 0.0147 | 0.0177 | 0.0146 | 0.0156 | 0.0145 | 0.0136 | 0.0138 | 0.0156 | 0.018 | *0.0174* | *0.0163* |
| DRB1*13:01 | 0.0677 | 0.0723 | 0.0624 | 0.0737 | 0.0813 | 0.0648 | 0.0615 | 0.0715 | 0.07 | 0.0492 | 0.0657 | 0.0674 | *0.0673* | *0.0666* |
| DRB1*13:02 | 0.0587 | 0.0543 | 0.0494 | 0.041 | 0.0567 | 0.0532 | 0.0423 | 0.0491 | 0.0503 | 0.059 | 0.0634 | 0.0475 | *0.0568* | *0.0546* |
| DRB1*13:03 | 0.0153 | 0.0115 | 0.0148 | 0.0207 | 0.0183 | 0.0122 | 0.0099 | 0.0091 | 0.0206 | 0.0106 | 0.015 | 0.0138 | *0.0151* | *0.0138* |
| DRB1*13:04 | 0 | 0 | 0 | 0 | 0 | 0.0009 | 0 | 0 | 0 | 0 | 0 | 0 | *0* | *0.0001* |
| DRB1*13:05 | 0.0011 | 0.0011 | 0.0013 | 0 | 0.0017 | 0.0019 | 0 | 0.0032 | 0 | 0.0009 | 0.0012 | 0.0007 | *0.0011* | *0.0011* |
| DRB1*13:11 | 0 | 0 | 0 | 0 | 0 | 0 | 0 | 0 | 0 | 0 | 0.0003 | 0 | *0* | *0* |
| DRB1*13:14 | 0.0004 | 0 | 0 | 0 | 0 | 0 | 0 | 0 | 0 | 0 | 0 | 0 | *0* | *0* |
| DRB1*13:15 | 0 | 0.0002 | 0 | 0 | 0 | 0 | 0 | 0 | 0 | 0 | 0 | 0 | *0* | *0* |
| DRB1*13:19 | 0 | 0 | 0 | 0 | 0 | 0 | 0 | 0 | 0 | 0.0004 | 0 | 0 | *0* | *0* |
| DRB1*13:21 | 0 | 0.0002 | 0 | 0 | 0 | 0 | 0 | 0 | 0 | 0 | 0 | 0 | *0* | *0* |
| ALLELES | AA (n=1838) | BE (n=3545) | BS (n=1888) | CF (n=408) | FR (n=367) | GE (n=1267) | GR (n=759) | LG (n=1169) | LS (n=993) | LU (n=1553) | SG (n=2113) | SI (n=832) | ZH (n=4875) | All (n=21607) |
| DRB1*13:24 | 0 | 0 | 0 | 0 | 0 | 0.0005 | 0 | 0 | 0 | 0 | 0 | 0 | *0* | *0* |
| DRB1*14:01/54 | 0.0386 | 0.0385 | 0.0385 | 0.0378 | 0.0381 | 0.0436 | 0.0369 | 0.0528 | 0.0428 | 0.0355 | 0.0412 | 0.0339 | *0.0376* | *0.0393* |
| DRB1*14:02 | 0 | 0 | 0.0004 | 0.0007 | 0.0018 | 0.0005 | 0 | 0.0005 | 0.0014 | 0 | 0 | 0 | *0.0002* | *0.0003* |
| DRB1*14:04 | 0 | 0.0006 | 0.002 | 0 | 0 | 0 | 0.0012 | 0.0007 | 0.0019 | 0.0013 | 0 | 0 | *0.0008* | *0.0007* |
| DRB1*14:05 | 0 | 0.0002 | 0 | 0 | 0 | 0 | 0 | 0 | 0 | 0 | 0 | 0 | *0* | *0* |
| DRB1*14:06 | 0 | 0 | 0 | 0 | 0 | 0 | 0 | 0.0005 | 0 | 0 | 0 | 0 | *0* | *0* |
| DRB1*14:07 | 0 | 0 | 0 | 0 | 0 | 0 | 0 | 0 | 0 | 0.0012 | 0 | 0 | *0.0008* | *0.0003* |
| DRB1*14:10 | 0 | 0.0002 | 0 | 0 | 0 | 0 | 0 | 0 | 0 | 0 | 0 | 0 | *0* | *0* |
| DRB1*14:16 | 0.0004 | 0.0002 | 0 | 0 | 0 | 0 | 0 | 0 | 0 | 0 | 0 | 0 | *0.0003* | *0.0001* |
| DRB1*14:17 | 0 | 0.0002 | 0.0004 | 0 | 0.0036 | 0 | 0 | 0 | 0 | 0 | 0.0004 | 0 | *0* | *0.0002* |
| DRB1*14:47 | 0 | 0 | 0 | 0.0007 | 0 | 0 | 0 | 0.0005 | 0 | 0 | 0 | 0 | *0* | *0* |
| DRB1*15:01 | 0.1068 | 0.1318 | 0.1123 | 0.0964 | 0.1228 | 0.0893 | 0.1309 | 0.0717 | 0.1052 | 0.1265 | 0.1039 | 0.093 | *0.1171* | *0.1122* |
| DRB1*15:02 | 0.0052 | 0.0043 | 0.0066 | 0.0119 | 0.0082 | 0.0104 | 0.0032 | 0.0082 | 0.0085 | 0.0069 | 0.0117 | 0.0018 | *0.0057* | *0.0066* |
| DRB1*15:03 | 0 | 0 | 0 | 0 | 0 | 0.0005 | 0.0009 | 0.0006 | 0 | 0.0005 | 0.0004 | 0 | *0.0002* | *0.0002* |
| DRB1*16 | 0.0287 | 0.0249 | 0.03 | 0.0237 | 0.0229 | 0.0325 | 0.0305 | 0.0469 | 0.0217 | 0.0316 | 0.03 | 0.0312 | *0.0257* | *0.0287* |
| blank | 0.0122 | 0.0017 | 0.0061 | 0.0033 | 0.0001 | 0.0103 | 0.0036 | 0.0064 | 0.0054 | 0 | 0.0081 | 0.0074 | *0.0078* | *0.0063* |
| Expected heterozygozity | 0.919 | 0.912 | 0.917 | 0.915 | 0.914 | 0.923 | 0.912 | 0.926 | 0.917 | 0.913 | 0.918 | 0.920 | 0.916 | 0.917 |
| HWE p-value | 0.021 | 0.106 | 0.063 | 0.515 | 0.865 | 0.036 | 0.406 | 0.039 | 0.176 | 0.572 | 0.059 | 0.242 | 0.0041 | 2.71E-10 |

**HLA-DQB1**

| ALLELES | AA (n=108) | BE (n=661) | BS (n=148) | GE (n=97) | GR (n=40) | LG (n=99) | LS (n=74) | LU (n=92) | SG (n=135) | SI (n=62) | ZH (n=292) | All (n=1808) |
| --- | --- | --- | --- | --- | --- | --- | --- | --- | --- | --- | --- | --- |
| DQB1*02:01 | 0.0605 | 0.0806 | 0.0796 | 0.1299 | 0.1156 | 0.1256 | 0.1338 | 0.0978 | 0.0846 | 0.1012 | 0.0911 | 0.0913 |
| DQB1*02:02 | 0.0969 | 0.1093 | 0.0852 | 0.0866 | 0.1156 | 0.0692 | 0.0824 | 0.1141 | 0.0952 | 0.08 | 0.0938 | 0.0982 |
| DQB1*03:01/09/19/21 | 0.2123 | 0.2211 | 0.1745 | 0.2457 | 0.2711 | 0.2501 | 0.2453 | 0.233 | 0.2272 | 0.2721 | 0.2292 | 0.2259 |
| DQB1*03:02 | 0.0869 | 0.094 | 0.0927 | 0.0868 | 0.0333 | 0.0395 | 0.1561 | 0.108 | 0.0988 | 0.0432 | 0.1116 | 0.0938 |
| DQB1*03:03 | 0.0434 | 0.0396 | 0.0499 | 0.0231 | 0.0166 | 0.0395 | 0.0446 | 0.0284 | 0.0546 | 0.0432 | 0.0339 | 0.0392 |
| DQB1*03:04 | 0 | 0 | 0 | 0 | 0 | 0 | 0 | 0.0057 | 0 | 0 | 0.0018 | 0.0006 |
| DQB1*03:05 | 0 | 0.0024 | 0.0073 | 0 | 0 | 0.0067 | 0 | 0 | 0 | 0 | 0.004 | 0.0025 |
| DQB1*04:02 | 0.0509 | 0.0363 | 0.0403 | 0.0258 | 0.0125 | 0.0505 | 0.0338 | 0.0489 | 0.0464 | 0.0323 | 0.024 | 0.0364 |
| DQB1*05:01 | 0.108 | 0.0961 | 0.1304 | 0.1306 | 0.0588 | 0.1047 | 0.0785 | 0.087 | 0.097 | 0.0828 | 0.1002 | 0.1008 |
| DQB1*05:02 | 0.0257 | 0.0238 | 0.0275 | 0.0237 | 0.0411 | 0.0481 | 0.0071 | 0.0109 | 0.0159 | 0.0256 | 0.0193 | 0.0233 |
| DQB1*05:03 | 0.0514 | 0.0369 | 0.0344 | 0.0356 | 0.0274 | 0.0767 | 0.0357 | 0.0272 | 0.0438 | 0.0426 | 0.0366 | 0.0395 |
| DQB1*05:04 | 0 | 0.0015 | 0 | 0.0059 | 0.0137 | 0 | 0.0071 | 0 | 0.0043 | 0 | 0.0058 | 0.0027 |
| DQB1*06:01 | 0.0049 | 0.0085 | 0 | 0.0115 | 0.0147 | 0.0057 | 0 | 0.0171 | 0.0044 | 0 | 0.0097 | 0.0075 |
| DQB1*06:02 | 0.1173 | 0.142 | 0.1383 | 0.0859 | 0.063 | 0.0687 | 0.0799 | 0.1025 | 0.0732 | 0.157 | 0.1296 | 0.1209 |
| DQB1*06:03 | 0.088 | 0.0652 | 0.0983 | 0.063 | 0.0999 | 0.0744 | 0.0799 | 0.074 | 0.0895 | 0.0683 | 0.055 | 0.0716 |
| DQB1*06:04/34 | 0.0538 | 0.0357 | 0.0328 | 0.0401 | 0.0293 | 0.0114 | 0.016 | 0.0399 | 0.0396 | 0.0356 | 0.0524 | 0.0375 |
| DQB1*06:08 | 0 | 0.0008 | 0 | 0 | 0 | 0 | 0 | 0 | 0 | 0 | 0 | 0.0003 |
| DQB1*06:09 | 0 | 0.0062 | 0.0073 | 0.0057 | 0.0147 | 0.0114 | 0 | 0.0057 | 0.0044 | 0 | 0.0019 | 0.0051 |
| blank | 0 | 0 | 0.0017 | 0 | 0.0726 | 0.0176 | 0 | 0 | 0.0212 | 0.0162 | 0 | 0.003 |
| Expected heterozygozity | 0.895 | 0.885 | 0.897 | 0.879 | 0.883 | 0.886 | 0.873 | 0.887 | 0.894 | 0.871 | 0.883 | 0.886 |
| HWE p-value | 1 | 0.175 | 1 | 1 | 0.481 | 0.540 | 1 | 1 | 0.609 | 0.854 | 1 | 0.023 |
