# Supplementary material for: The Heterogeneous HLA Genetic Makeup of the Swiss Population
Source: PLoS One. 2012 Jul 25;7(7):e41400. doi: 10.1371/journal.pone.0041400 (PMC3405111; doi:10.1371/journal.pone.0041400)
Supplement: Supporting Information S2 — Box-and-whisker diagrams. (DOC) [file pone.0041400.s002.doc]

**Supporting Information S2 – Box-and-whisker diagrams**

Box-and-whisker diagrams for allele (HLA-A, -B, -C, -DRB1 and -DQB1) and haplotype (HLA-A-B-C-DRB1, HLA-A-B-C, HLA-A-B-DRB1, HLA-A-B, HLA-A-C, HLA-A-DRB1, HLA-B-C, HLA-B-DRB1, HLA-C-DRB1, HLA-DRB1-DQB1) frequencies estimated for the data of the Swiss registry. Note that the scale used on the vertical axis for frequency is variable between diagrams. The bottom and top of each box are the 25th and 75th percentiles, respectively, while the band near the middle of the box is the 50th percentile (median). The whiskers represent the lowest/highest datum still within 1.5 IQR (i.e. the interquartile range). Pooled frequencies estimated for the whole Swiss registry are depicted with filled red circles (here given for informative purposes as Hardy Weinberg equilibrium hypothesis is rejected at HLA-A, -B, -DRB1 and -DQB1), while outlier regions (labeled) are indicated with open circles.

Recruitment centers where Hardy-Weinberg equilibrium hypothesis is rejected (after Bonferroni’s correction) are not taken into account on the diagrams (BS and ZH when HLA-B is involved and ZH when HLA-DRB1 is involved, respectively).

All: pooled registry, AA: Aargau-Solothurn, BE: Bern, BS: Basel, CF: La Chaux-de-Fonds (Neuchâtel and Jura), FR: Fribourg, GE: Genève, GR: Graubünden, LG: Lugano (Svizzera Italiana), LS: Lausanne (Vaud), LU: Luzern (Zentralschweiz), SG: St. Gallen (Nordost-Schweiz), SI: Sion (Valais) and ZH: Zürich.

**HLA-A alleles with a frequency ≥ 5% in at least one regional service**

Legend

1: A*01:01/04N/22N

2: A*02

3: A*03

4: A*11

5: A*24

6: A*26:01/24/26

7: A*29:02

8: A*31:01/14N

9: A*32:01

10: A*68:01/11N/33

**HLA-B alleles with a frequency ≥ 5% in at least one regional service**

Legend

1: B*07:02/44/49N/58/59/61

2: B*08:01/19N

3: B*15:01/102/104/140/146

4: B*18:01/17N

5: B*35:01/40N/42/57/94

6: B*35:03/70

7: B*40:01/55

8: B*44:02/19N/27

9: B*44:03

10: B*51:01/11N/30/32/48/51

11: B*57:01

**HLA-C alleles with a frequency ≥ 5% in at least one regional service**

Legend

1: C*02:02

2: C*03:03/20N

3: C*03:04

4: C*0401/09N/28/30

5: C*05:01/03

6: C*06:02

7: C*07:01/06/18/52

8: C*07:02/50

9: C*08:02

10: C*12:03

11: C*15:02/13

12: C*16:01

**HLA-DRB1 alleles with a frequency ≥ 5% in at least one regional service**

Legend

1:DRB1*01:01

2:DRB1*03

3:DRB1*04

4:DRB1*07

5:DRB1*11:01

6:DRB1*11:04

7:DRB1*13:01

8:DRB1*13:02

9:DRB1*14:01/54

10:DRB1*15:01

**HLA-DQB1 alleles with a frequency ≥ 5% in at least one regional service**

Legend

1:DQB1*02:01

2:DQB1*02:02

3:DQB1*03:01/09/19/21

4:DQB1*03:02

5:DQB1*03:03

6:DQB1*04:02

7:DQB1*05:01

8:DQB1*05:03

9:DQB1*06:02

10:DQB1*06:03

11:DQB1*06:04/34

12: blank

**HLA-A-B-C-DRB1 haplotypes with a frequency ≥ 3% in at least one regional service**

Legend

1: A*01~B*08~C*07:01/ 06/18/52~DRB1*03

2: A*02~B*07~C*07:02/50~DRB1*07

3: A*02~B*07~C*07:02/50~DRB1*15

4: A*02~B*44~C*05:01/03~DRB1*01

5: A*02~B*44~C*05:01/03~DRB1*11

6: A*02~B*44~C*05:01/03~DRB1*15

7: A*02~B*50~C*06:02~DRB1*07

8: A*03~B*07~C*07:02/50~DRB1*13

9: A*03~B*07~C*07:02/50~DRB1*15

10: A*03~B*35~C*04:01/09N/28/30~DRB1*11

11: A*11~B*35~C*04:01/09N/28/30~DRB1*01

12: A*24~B*07~C*07:02/50~DRB1*15

13: A*29~B*44~C*16:01~DRB1*07

14: A*33~B*14~C*08:02~DRB1*01

**HLA-A-B-C haplotypes with a frequency ≥ 3% in at least one regional service**

Legend

1: A*01~B*08~C*07:01/ 06/18/52

2: A*01~B*35~C*04:01/09N/28/30

3: A*01~B*57~C*06:02

4: A*02~B*07~C*07:02/50

5: A*02~B*08~C*07:01/ 06/18/52

6: A*02~B*15~C*03:03/20N

7: A*02~B*35~C*04:01/09N/28/30

8: A*02~B*40~C*03:04

9: A*02~B*44~C*05:01/03

10: A*02~B*50~C*06:02

11: A*02~B*51~C*14:02

12: A*03~B*07~C*07:02/50

13: A*03~B*35~C*04:01/09N/28/30

14: A*11~B*35~C*04:01/09N/28/30

15: A*24~B*07~C*07:02/50

16: A*24~B*35~C*04:01/09N/28/30

17: A*29~B*44~C*16:01

18: A*30~B*18~C*05:01/03

19: A*33~B*14~C*08:02

**HLA-A-B-DRB1 haplotypes with a frequency ≥ 3% in at least one regional service**

Legend

1: A*01~B*08~DRB1*03

2: A*02~B*07~DRB1*15

3: A*02~B*40~DRB1*13

4: A*02~B*44~DRB1*07

5: A*02~B*44~DRB1*15

6: A*02~B*51~DRB1*11

7: A*03~B*07~DRB1*15

8: A*24~B*35~DRB1*11

**HLA-A-B haplotypes with a frequency ≥ 3% in at least one regional service**

Legend

1: A*01~B*08

2: A*01~B*57:01

3: A*02~B*07

4: A*02~B*08

5: A*02~B*15

6: A*02~B*40:01/55

7: A*02~B*44:02/19N/27

8: A*02~B*51

9: A*03~B*07

10: A*29~B*44:03

**HLA-A-C haplotypes with a frequency ≥ 3% in at least one regional service**

Legend

1: A*01~C*06:02

2: A*01~C*07:01/ 06/18/52

3: A*02~C*03:03/20N

4: A*02~C*03:04

5: A*02~C*04:01/09N/28/30

6: A*02~C*05:01/03

7: A*02~C*06:02

8: A*02~C*07:01/ 06/18/52

9: A*02~C*07:02/50

10: A*02~C*12:03

11: A*02~C*14:02

12: A*03~C*04:01/09N/28/30

13: A*03~C*07:02/50

14: A*11~C*04:01/09N/28/30

15: A*23~C*04:01/09N/28/30

16: A*24~C*04:01/09N/28/30

17: A*24~C*06:02

18: A*24~C*07:01/ 06/18/52

19: A*24~C*07:02/50

20: A*29~C*16:01

21: A*30:01/24~C*06:02

22: A*30:02~C*05:01/03

23: A*33~C*08:02

**HLA-A-DRB1 haplotypes with a frequency ≥ 3% in at least one regional service**

Legend

1: A*01~DRB1*03

2: A*01~DRB1*11

3: A*02~DRB1*01:01

4: A*02~DRB1*03

5: A*02~DRB1*04

6: A*02~DRB1*07

7: A*02~DRB1*08

8: A*02~DRB1*11

9: A*02~DRB1*13:01

10: A*02~DRB1*13:02

11: A*02~DRB1*14

12: A*02~DRB1*15:01

13: A*03~DRB1*01:01

14: A*03~DRB1*11

15: A*03~DRB1*15:01

16: A*24~DRB1*11

17: A*29~DRB1*07

18: A*30:02~DRB1*03

19: A*68~DRB1*11

**HLA-B-C haplotypes with a frequency ≥ 3% in at least one regional service**

Legend

1: B*07~C*07:02/50

2: B*08~C*07:01/ 06/18/52

3: B*13:02~C*06:02

4: B*14:02~C*08:02

5: B*15~C*03:03/20N

6: B*18~C*05:01/03

7: B*18~C*07:01/ 06/18/52

8: B*35:01/40N/42/57/94~C*04:01/09N/28/30

9: B*35:03/70~C*04:01/09N/28/30

10: B*38:01~C*12:03

11: B*40:01/55~C*03:04

12: B*44:02/19N/27~C*05:01/03

13: B*44:03~C*04:01/09N/28/30

14: B*44:03~C*16:01

15: B*49:01~C*07:01/ 06/18/52

16: B*51~C*01:02

17: B*51~C*14:02

18: B*51~C*15:02/13

19: B*57:01~C*06:02

**HLA-B-DRB1 haplotypes with a frequency ≥ 3% in at least one regional service**

Legend

1: B*07~DRB1*15:01

2: B*08~DRB1*03

3: B*15~DRB1*04

4: B*15~DRB1*11

5: B*35:01/40N/42/57/94~DRB1*01:01

6: B*44:02/19N/27~DRB1*04

7: B*44:03~DRB1*07

8: B*51~DRB1*11

9: B*57:01~DRB1*07

**HLA-C-DRB1 haplotypes with a frequency ≥ 3% in at least one regional service**

Legend

1: C*04:01/09N/28/30~DRB1*01:01

2: C*04:01/09N/28/30~DRB1*11

3: C*04:01/09N/28/30~DRB1*15:01

4: C*05:01/03~DRB1*04

5: C*05:01/03~DRB1*11

6: C*06:02~DRB1*07

7: C*07:01/ 06/18/52~DRB1*03

8: C*07:01/ 06/18/52~DRB1*11

9: C*07:02/50~DRB1*15:01

10: C*16:01~DRB1*07

**HLA-DRB1-DQB1 haplotypes with a frequency ≥ 3% in at least one regional service**

Legend

1:DRB1*01:01~DQB1*05:01

2:DRB1*03~DQB1*02:01

3:DRB1*04~DQB1*03:01/09/19/21

4:DRB1*04~DQB1*03:02

5:DRB1*07~DQB1*02:02

6:DRB1*07~DQB1*03:03

7:DRB1*08~DQB1*04:02

8:DRB1*11~DQB1*03:01/09/19/21

9:DRB1*13:01~DQB1*06:03

10:DRB1*13:02~DQB1*06:04/34

11:DRB1*14~DQB1*05:03

12:DRB1*15:01~DQB1*06:02

13:DRB1*16~DQB1*0502
