# Supplementary material for: The Heterogeneous HLA Genetic Makeup of the Swiss Population
Source: PLoS One. 2012 Jul 25;7(7):e41400. doi: 10.1371/journal.pone.0041400 (PMC3405111; doi:10.1371/journal.pone.0041400)
Supplement: Supporting Information S3 — Allele frequencies histograms in three main geographic areas of Switzerland. (DOC) [file pone.0041400.s003.doc]

**Supporting Information S3 – Allele frequencies histograms in three main geographic areas of Switzerland**

Graphics of allele frequencies for HLA-A, -B, -C, -DRB1 and -DQB1. The subset of alleles represented here is selected according to a frequency threshold of 3% in at least one recruitment center. For each allele, recruitment centers are subdivided into geographical regions shown by different colors (Western central plateau and Jura in light blue, Eastern central plateau and Jura in dark blue, Alps in red). Recruitment centers where Hardy-Weinberg equilibrium hypothesis is rejected (after Bonferroni’s correction) are not included on the graphics (BS and ZH at HLA-B and ZH at HLA-DRB1, respectively).

AA: Aargau-Solothurn, BE: Bern, BS: Basel, CF: La Chaux-de-Fonds (Neuchâtel and Jura), FR: Fribourg, GE: Genève, GR: Graubünden, LG: Lugano (Svizzera Italiana), LS: Lausanne (Vaud), LU: Luzern (Zentralschweiz), SG: St. Gallen (Nordost-Schweiz), SI: Sion (Valais) and ZH: Zürich.

**HLA-A**

**HLA-B**

**HLA-C**

**HLA-DRB1**

**HLA-DQB1**

Note: the relatively high “blank” frequency in GR is due to the presence of 12 apparent homozygotes among a total of 40 donors genotyped at this locus. Indeed, all apparent homozygotes are either true homozygotes or heterozygotes for both the known allele and a”blank” (i.e. undefined) allele used to represent a still unknown or undetectable allele. The need for such a “blank” allele is due to the impossibility of most typing methods to describe the entire set of possible alleles at HLA loci (See Supplementary information S11). The final “blank allele” frequency may correspond to the frequency of one or several “blank” alleles together.
