# Supplementary material for: The Heterogeneous HLA Genetic Makeup of the Swiss Population
Source: PLoS One. 2012 Jul 25;7(7):e41400. doi: 10.1371/journal.pone.0041400 (PMC3405111; doi:10.1371/journal.pone.0041400)
Supplement: Supporting Information S4 — Haplotype frequencies. (DOC) [file pone.0041400.s004.doc]

**Supporting Information S4 – Haplotype frequencies**

Only haplotypes with a frequency ≥ 3% (for 4 loci) or ≥ 1% (for 3 and 2 loci) in at least one recruitment center are listed.

Frequencies are listed in italic when HWE null hypothesis is rejected at one or several loci (after Bonferroni’s correction) and, therefore, should not be considered as representative of the national or local population (here given for informative purposes).

List of abbreviations used in the below tables:

AA: Aargau-Solothurn, BE: Bern, BS: Basel, GE: Genève, GR: Graubünden, LG: Lugano (Svizzera Italiana), LS: Lausanne (Vaud), LU: Luzern (Zentralschweiz), SG: St. Gallen (Nordost-Schweiz), SI: Sion (Valais), ZH: Zürich and All: all recruitment centers pooled together.

**HLA-A-B-C-DRB1**

| HAPLOTYPES | AA (n=55) | BE (n=561) | BS (n=77) | GE (n=50) | GR (n=96) | LG (n=66) | LS (n=127) | LU (n=72) | SG (n=75) | SI (n=74) | ZH (n=151) | All (n=1404) |
| --- | --- | --- | --- | --- | --- | --- | --- | --- | --- | --- | --- | --- |
| A*01~B*08~C*07:01/06/18/52~DRB1*03 | 0.0273 | 0.0466 | *0.0195* | 0.06 | 0.0208 | 0.0379 | 0.0585 | 0.0208 | 0.0467 | 0.027 | *0.0358* | *0.0393* |
| A*02~B*07~C*07:02/50~DRB1*07 | 0 | 0 | *0.0044* | 0.03 | 0 | 0 | 0 | 0 | 0 | 0 | *0.0015* | *0.0022* |
| A*02~B*07~C*07:02/50~DRB1*15 | 0.0213 | 0.0145 | *0.0195* | 0.01 | 0 | 0.0102 | 0 | 0.0208 | 0.0467 | 0.0135 | *0.0232* | *0.0156* |
| A*02~B*44~C*05:01/03~DRB1*01 | 0.0182 | 0 | *0* | 0.0044 | 0.0312 | 0 | 0 | 0 | 0.0067 | 0 | *0.0033* | *0.0028* |
| A*02~B*44~C*05:01/03~DRB1*11 | 0 | 0.0044 | *0.013* | 0.03 | 0 | 0 | 0.0118 | 0 | 0 | 0.0203 | *0* | *0.0056* |
| A*02~B*44~C*05:01/03~DRB1*15 | 0.0333 | 0 | *0* | 0 | 0 | 0.0009 | 0.0079 | 0 | 0 | 0 | *0* | *0.0025* |
| A*02~B*50~C*06:02~DRB1*07 | 0.0182 | 0.0036 | *0* | 0.03 | 0.0104 | 0 | 0.0037 | 0.0139 | 0 | 0 | *0.0066* | *0.0077* |
| A*03~B*07~C*07:02/50~DRB1*13 | 0 | 0.0043 | *0* | 0 | 0 | 0 | 0.0317 | 0.0208 | 0.0133 | 0.0068 | *0.0066* | *0.0076* |
| A*03~B*07~C*07:02/50~DRB1*15 | 0.0091 | 0.036 | *0.0325* | 0 | 0.0365 | 0.0126 | 0.0215 | 0.0278 | 0.0067 | 0.0203 | *0.0232* | *0.0262* |
| A*03~B*35~C*04:01/09N/28/30~DRB1*11 | 0 | 0 | *0* | 0 | 0 | 0 | 0 | 0.0347 | 0 | 0 | *0.0021* | *0.003* |
| A*11~B*35~C*04:01/09N/28/30~DRB1*01 | 0 | 0.0072 | *0.0325* | 0 | 0.026 | 0.0076 | 0 | 0 | 0 | 0 | *0.0232* | *0.0084* |
| A*24~B*07~C*07:02/50~DRB1*15 | 0 | 0.0112 | *0* | 0.03 | 0 | 0 | 0.0118 | 0 | 0.0038 | 0 | *0.0166* | *0.0085* |
| A*29~B*44~C*16:01~DRB1*07 | 0.0182 | 0.016 | *0.013* | 0.02 | 0.0208 | 0.0152 | 0.0157 | 0.0312 | 0.0133 | 0.0135 | *0.0099* | *0.0167* |
| A*33~B*14~C*08:02~DRB1*01 | 0.0091 | 0.001 | *0.013* | 0 | 0.0052 | 0.0303 | 0 | 0 | 0 | 0 | *0.0132* | *0.0057* |

**HLA-A-B-C**

| HAPLOTYPES | AA (n=55) | BE (n=561) | BS (n=77) | GE (n=50) | GR (n=96) | LG (n=66) | LS (n=127) | LU (n=72) | SG (n=75) | SI (n=74) | ZH (n=151) | All (n=1404) |
| --- | --- | --- | --- | --- | --- | --- | --- | --- | --- | --- | --- | --- |
| A*01~B*08~C*07:01/06/18/52 | 0.0364 | 0.0612 | *0.026* | 0.08 | 0.0357 | 0.0379 | 0.0747 | 0.0278 | 0.0524 | 0.0608 | *0.0525* | *0.0545* |
| A*01~B*15~C*03:03/20N | 0 | 0.0011 | *0.0161* | 0 | 0 | 0 | 0 | 0 | 0.0076 | 0 | *0* | *0.0013* |
| A*01~B*35~C*04:01/09N/28/30 | 0 | 0.0033 | *0.0455* | 0 | 0 | 0 | 0 | 0.0139 | 0 | 0 | *0* | *0.0046* |
| A*01~B*35~C*12:03 | 0 | 0.0009 | *0* | 0.01 | 0 | 0 | 0.002 | 0 | 0 | 0.0023 | *0* | *0.001* |
| A*01~B*37~C*06:02 | 0 | 0.0044 | *0* | 0 | 0.0106 | 0 | 0.0112 | 0 | 0.0133 | 0.0135 | *0.0066* | *0.0069* |
| A*01~B*38~C*12:03 | 0.0182 | 0.0014 | *0* | 0 | 0 | 0 | 0 | 0 | 0 | 0 | *0* | *0.0009* |
| A*01~B*44~C*04:01/09N/28/30 | 0 | 0 | *0* | 0 | 0 | 0 | 0 | 0.0139 | 0 | 0 | *0.0033* | *0* |
| A*01~B*44~C*05:01/03 | 0 | 0 | *0* | 0 | 0 | 0 | 0.0043 | 0.0069 | 0 | 0.0068 | *0.01* | *0.0028* |
| A*01~B*51~C*01:02 | 0 | 0.0019 | *0.013* | 0 | 0 | 0 | 0 | 0 | 0 | 0 | *0* | *0.0003* |
| A*01~B*52~C*12:02 | 0.0182 | 0.0036 | *0.0065* | 0 | 0.0104 | 0 | 0 | 0 | 0.0067 | 0 | *0.0036* | *0.0041* |
| A*01~B*57~C*06:02 | 0.0136 | 0.0081 | *0.0163* | 0 | 0.0037 | 0.0227 | 0.027 | 0.0069 | 0.0333 | 0.027 | *0.0129* | *0.0138* |
| A*01~blank~C*06:02 | 0.0127 | 0 | *0* | 0 | 0 | 0 | 0 | 0 | 0 | 0 | *0* | *0* |
| A*02~B*07~C*07:02/50 | 0.0182 | 0.0256 | *0.0371* | 0.04 | 0.0469 | 0.0087 | 0 | 0.0342 | 0.079 | 0.0338 | *0.0265* | *0.0255* |
| A*02~B*08~C*07:01/06/18/52 | 0 | 0.0109 | *0* | 0 | 0.0041 | 0.0455 | 0.015 | 0.0052 | 0.021 | 0 | *0.0034* | *0.0105* |
| A*02~B*13~C*06:02 | 0.0182 | 0.0071 | *0.013* | 0 | 0 | 0.0071 | 0.0039 | 0 | 0 | 0 | *0* | *0.0074* |
| A*02~B*14~C*08:02 | 0.0182 | 0.0049 | *0* | 0.02 | 0.0052 | 0 | 0.0043 | 0.0046 | 0 | 0.0135 | *0.0099* | *0.0061* |
| A*02~B*15~C*03:03/20N | 0.0545 | 0.0229 | *0.0169* | 0.01 | 0.0203 | 0 | 0.0118 | 0.0139 | 0.018 | 0 | *0.0331* | *0.0196* |
| A*02~B*15~C*03:04 | 0 | 0.0066 | *0* | 0.01 | 0.0043 | 0.0038 | 0 | 0 | 0.021 | 0.0048 | *0* | *0.0071* |
| A*02~B*15~C*04:01/09N/28/30 | 0.022 | 0.0012 | *0* | 0 | 0.0104 | 0 | 0 | 0 | 0 | 0 | *0.0066* | *0.0035* |
| A*02~B*18~C*05:01/03 | 0.0068 | 0.0009 | *0.0035* | 0.01 | 0 | 0.0227 | 0 | 0 | 0 | 0.0068 | *0.0025* | *0.0023* |
| A*02~B*18~C*07:01/06/18/52 | 0 | 0.0092 | *0.026* | 0.01 | 0.0023 | 0.0129 | 0.0039 | 0 | 0.0016 | 0 | *0* | *0.0083* |
| A*02~B*27~C*01:02 | 0 | 0.0067 | *0* | 0 | 0 | 0.0019 | 0.0197 | 0 | 0 | 0.0135 | *0* | *0.0057* |
| A*02~B*27~C*02:02 | 0 | 0.001 | *0* | 0 | 0 | 0 | 0 | 0.0278 | 0.0067 | 0 | *0.0099* | *0.0049* |
| A*02~B*35~C*04:01/09N/28/30 | 0 | 0.0157 | *0.0195* | 0 | 0.0129 | 0.0117 | 0.0197 | 0 | 0 | 0.0518 | *0* | *0.0108* |
| A*02~B*37~C*06:02 | 0 | 0.0017 | *0.0195* | 0 | 0 | 0 | 0.0006 | 0 | 0 | 0 | *0* | *0.0023* |
| A*02~B*39~C*12:03 | 0.0015 | 0.0053 | *0.0011* | 0 | 0 | 0.0152 | 0.0039 | 0 | 0 | 0 | *0.0066* | *0.0038* |
| A*02~B*40~C*03:04 | 0.0182 | 0.0205 | *0.0195* | 0.02 | 0 | 0 | 0.0315 | 0 | 0.0171 | 0 | *0.0166* | *0.0149* |
| A*02~B*44~C*04:01/09N/28/30 | 0 | 0.0041 | *0* | 0 | 0.0104 | 0 | 0.0079 | 0.0139 | 0.0054 | 0 | *0* | *0.0022* |
| A*02~B*44~C*05:01/03 | 0.0547 | 0.035 | *0.0195* | 0.05 | 0.0168 | 0.0152 | 0.0218 | 0.0208 | 0.0372 | 0.0203 | *0.0264* | *0.0303* |
| A*02~B*44~C*16:01 | 0 | 0.0019 | *0* | 0 | 0 | 0 | 0.0039 | 0 | 0.0133 | 0 | *0.0008* | *0.0027* |
| A*02~B*45~C*06:02 | 0 | 0 | *0* | 0 | 0 | 0 | 0 | 0 | 0 | 0.0135 | *0* | *0* |
| A*02~B*50~C*06:02 | 0.0182 | 0.0042 | *0.013* | 0.03 | 0.0156 | 0.0038 | 0.0079 | 0.0278 | 0 | 0 | *0.0066* | *0.0098* |
| HAPLOTYPES | AA (n=55) | BE (n=561) | BS (n=77) | GE (n=50) | GR (n=96) | LG (n=66) | LS (n=127) | LU (n=72) | SG (n=75) | SI (n=74) | ZH (n=151) | All (n=1404) |
| A*02~B*51~C*01:02 | 0 | 0.0061 | *0* | 0 | 0.0208 | 0 | 0 | 0.0117 | 0.0067 | 0 | *0.0099* | *0.0061* |
| A*02~B*51~C*02:02 | 0 | 0 | *0* | 0.02 | 0.0026 | 0 | 0.0118 | 0 | 0 | 0.0024 | *0* | *0.0046* |
| A*02~B*51~C*04:01/09N/28/30 | 0 | 0.0019 | *0* | 0 | 0 | 0.0152 | 0 | 0 | 0 | 0 | *0* | *0.0013* |
| A*02~B*51~C*07:01/06/18/52 | 0.0091 | 0 | *0* | 0 | 0 | 0.0152 | 0 | 0 | 0 | 0 | *0* | *0.0013* |
| A*02~B*51~C*14:02 | 0.0273 | 0.0058 | *0* | 0 | 0.0208 | 0.053 | 0 | 0 | 0 | 0 | *0.0149* | *0.0114* |
| A*02~B*51~C*15:02/13 | 0.0271 | 0.0194 | *0.0028* | 0.01 | 0 | 0.0152 | 0.0059 | 0.0051 | 0 | 0.0045 | *0.0232* | *0.0131* |
| A*02~B*55~C*03:03/20N | 0 | 0.0032 | *0* | 0 | 0 | 0 | 0.0004 | 0 | 0 | 0 | *0.0149* | *0.0037* |
| A*02~B*57~C*06:02 | 0.0072 | 0.0082 | *0.0161* | 0.0025 | 0.0156 | 0 | 0 | 0.0069 | 0 | 0.0068 | *0* | *0.0067* |
| A*02~blank~blank | 0 | 0 | *0* | 0 | 0 | 0 | 0.0062 | 0.0027 | 0.0133 | 0 | *0* | *0* |
| A*03~B*07~C*07:02/50 | 0 | 0.0644 | *0.0514* | 0 | 0.0625 | 0.0276 | 0.0748 | 0.072 | 0.0267 | 0.0322 | *0.0356* | *0.0514* |
| A*03~B*07~C*07:29 | 0 | 0 | *0* | 0 | 0 | 0 | 0 | 0.0105 | 0 | 0 | *0* | *0.0004* |
| A*03~B*15~C*03:03/20N | 0 | 0.001 | *0* | 0 | 0 | 0 | 0.0079 | 0 | 0 | 0.0162 | *0* | *0.0029* |
| A*03~B*18~C*07:01/06/18/52 | 0 | 0.0018 | *0.0006* | 0.0024 | 0.0025 | 0.0174 | 0 | 0.0069 | 0 | 0 | *0* | *0.0035* |
| A*03~B*27~C*01:02 | 0 | 0.0042 | *0* | 0.01 | 0 | 0 | 0 | 0 | 0 | 0 | *0* | *0.0023* |
| A*03~B*35~C*04:01/09N/28/30 | 0.0364 | 0.0262 | *0.02* | 0.02 | 0.0104 | 0 | 0.0236 | 0.0625 | 0.0133 | 0.0131 | *0.0356* | *0.0236* |
| A*03~B*38~C*12:03 | 0 | 0 | *0* | 0.0025 | 0.0104 | 0 | 0 | 0 | 0 | 0.0034 | *0.0028* | *0.0018* |
| A*03~B*39~C*07:02/50 | 0 | 0.001 | *0* | 0.02 | 0 | 0 | 0.0039 | 0 | 0 | 0 | *0* | *0.0009* |
| A*03~B*40~C*03:04 | 0 | 0.0054 | *0.0195* | 0 | 0 | 0.0076 | 0 | 0 | 0 | 0 | *0.0022* | *0.0044* |
| A*03~B*41~C*04:01/09N/28/30 | 0 | 0 | *0* | 0.01 | 0 | 0 | 0 | 0 | 0 | 0 | *0* | *0.0004* |
| A*03~B*44~blank | 0 | 0 | *0* | 0 | 0 | 0.0152 | 0 | 0 | 0 | 0 | *0* | *0.0005* |
| A*03~B*44~C*04:01/09N/28/30 | 0.0091 | 0 | *0* | 0.0025 | 0 | 0 | 0.0039 | 0 | 0 | 0.0135 | *0* | *0.0011* |
| A*03~B*44~C*05:01/03 | 0 | 0.0083 | *0* | 0 | 0 | 0 | 0 | 0.0069 | 0 | 0 | *0.0166* | *0.0073* |
| A*03~B*51~C*01:02 | 0.0182 | 0 | *0* | 0 | 0 | 0 | 0 | 0 | 0 | 0 | *0.0132* | *0.0021* |
| A*03~B*51~C*12:03 | 0 | 0 | *0* | 0.01 | 0 | 0 | 0 | 0 | 0 | 0 | *0* | *0.0004* |
| A*03~B*51~C*15:02/13 | 0.0091 | 0.0017 | *0* | 0 | 0 | 0.0152 | 0 | 0 | 0 | 0.0022 | *0.0066* | *0.0037* |
| A*03~B*52~C*12:02 | 0.0091 | 0 | *0* | 0.01 | 0 | 0 | 0 | 0 | 0.0017 | 0 | *0* | *0.0008* |
| A*11~B*14~C*08:02 | 0 | 0.0018 | *0.0065* | 0.02 | 0 | 0 | 0 | 0 | 0 | 0 | *0* | *0.001* |
| A*11~B*35~C*04:01/09N/28/30 | 0 | 0.0222 | *0.032* | 0.0267 | 0.0465 | 0.0379 | 0.0197 | 0.0069 | 0.0133 | 0.0048 | *0.0118* | *0.0222* |
| A*11~B*44~blank | 0 | 0 | *0* | 0 | 0 | 0 | 0 | 0 | 0.0133 | 0 | *0* | *0* |
| A*11~B*44~C*05:01/03 | 0 | 0.0046 | *0* | 0.0049 | 0 | 0 | 0.0039 | 0 | 0.0067 | 0.0135 | *0* | *0.0033* |
| A*11~B*44~C*16:01 | 0.0091 | 0 | *0* | 0 | 0.0104 | 0 | 0 | 0 | 0 | 0 | *0* | *0.0012* |
| A*11~B*44~C*16:04 | 0 | 0 | *0* | 0 | 0 | 0 | 0 | 0.0139 | 0 | 0 | *0* | *0* |
| A*11~B*51~C*03:03/20N | 0 | 0 | *0* | 0 | 0.0104 | 0 | 0.0118 | 0 | 0 | 0 | *0* | *0.0008* |
| A*11~B*51~C*15:02/13 | 0.0273 | 0.0076 | *0* | 0 | 0 | 0.0056 | 0 | 0 | 0 | 0.0134 | *0.0099* | *0.0068* |
| HAPLOTYPES | AA (n=55) | BE (n=561) | BS (n=77) | GE (n=50) | GR (n=96) | LG (n=66) | LS (n=127) | LU (n=72) | SG (n=75) | SI (n=74) | ZH (n=151) | All (n=1404) |
| A*11~B*55~C*03:03/20N | 0.0091 | 0.0061 | *0* | 0 | 0.0052 | 0 | 0 | 0.0061 | 0.01 | 0.0135 | *0.0033* | *0.0062* |
| A*11~blank~blank | 0 | 0 | *0* | 0.0133 | 0 | 0 | 0.0002 | 0 | 0 | 0 | *0* | *0* |
| A*23~B*44~C*04:01/09N/28/30 | 0.0182 | 0.0107 | *0.0065* | 0.0025 | 0.0052 | 0.0025 | 0.0118 | 0 | 0 | 0.027 | *0.0132* | *0.0117* |
| A*23~B*49~C*07:01/06/18/52 | 0 | 0.0114 | *0* | 0.0002 | 0.0017 | 0.0031 | 0.001 | 0 | 0 | 0 | *0.0066* | *0.0066* |
| A*23~B*50~C*06:02 | 0 | 0.0019 | *0* | 0 | 0.0104 | 0 | 0 | 0.0069 | 0 | 0.0068 | *0.0017* | *0.003* |
| A*24~B*07~C*07:02/50 | 0.0182 | 0.017 | *0* | 0.03 | 0 | 0 | 0.0197 | 0 | 0.0133 | 0 | *0.0207* | *0.0162* |
| A*24~B*07~C*07:24 | 0 | 0 | *0* | 0 | 0 | 0.0152 | 0 | 0 | 0 | 0 | *0* | *0.0004* |
| A*24~B*08~C*07:01/06/18/52 | 0 | 0.0011 | *0.0195* | 0 | 0.0052 | 0 | 0.0018 | 0.0208 | 0 | 0.0025 | *0* | *0.0042* |
| A*24~B*13~C*06:02 | 0 | 0.002 | *0.0032* | 0 | 0 | 0.0019 | 0.0039 | 0.0139 | 0.0067 | 0 | *0.0066* | *0.0026* |
| A*24~B*14~C*08:02 | 0 | 0.0025 | *0.013* | 0.02 | 0.0122 | 0 | 0 | 0 | 0 | 0.0203 | *0* | *0.0034* |
| A*24~B*15~C*03:03/20N | 0 | 0.0088 | *0.0163* | 0 | 0.0052 | 0 | 0 | 0 | 0 | 0.0176 | *0.0118* | *0.0087* |
| A*24~B*18~C*03:04 | 0.0182 | 0 | *0* | 0 | 0 | 0 | 0 | 0 | 0 | 0 | *0* | *0.0004* |
| A*24~B*18~C*07:01/06/18/52 | 0 | 0.0053 | *0* | 0 | 0.0026 | 0.0152 | 0 | 0 | 0 | 0 | *0* | *0.0033* |
| A*24~B*35~C*04:01/09N/28/30 | 0.0182 | 0.0197 | *0.013* | 0 | 0 | 0.053 | 0.0079 | 0.0139 | 0.0533 | 0.0095 | *0.0231* | *0.0188* |
| A*24~B*38~C*12:03 | 0.0015 | 0 | *0.013* | 0 | 0 | 0 | 0.0008 | 0 | 0 | 0 | *0.0074* | *0.0026* |
| A*24~B*44~C*05:01/03 | 0 | 0 | *0* | 0.0025 | 0.0243 | 0 | 0 | 0.0069 | 0 | 0 | *0* | *0.0011* |
| A*24~B*44~C*07:04/11 | 0 | 0.0009 | *0* | 0 | 0.0104 | 0 | 0 | 0 | 0 | 0 | *0* | *0.0007* |
| A*24~B*44~C*16:01 | 0 | 0 | *0* | 0.01 | 0.0052 | 0 | 0.0043 | 0 | 0 | 0 | *0.0066* | *0.003* |
| A*24~B*51~C*01:02 | 0 | 0 | *0* | 0.01 | 0.0104 | 0 | 0 | 0 | 0 | 0 | *0* | *0.0024* |
| A*24~B*52~C*12:02 | 0 | 0 | *0.0016* | 0.01 | 0 | 0 | 0.0039 | 0 | 0 | 0 | *0* | *0.0006* |
| A*24~B*55~C*03:03/20N | 0 | 0 | *0* | 0.02 | 0 | 0 | 0.0079 | 0 | 0.0017 | 0 | *0.0033* | *0.0022* |
| A*24~B*57~C*06:02 | 0 | 0.0038 | *0.0016* | 0 | 0 | 0.0227 | 0.0071 | 0.005 | 0.02 | 0 | *0* | *0.005* |
| A*25~B*18~C*12:03 | 0 | 0.0098 | *0.0195* | 0.0025 | 0.0004 | 0.0076 | 0.001 | 0.0139 | 0 | 0 | *0* | *0.007* |
| A*26~B*13~C*06:02 | 0 | 0 | *0* | 0 | 0.0104 | 0 | 0 | 0 | 0 | 0 | *0* | *0* |
| A*26~B*18~C*01:02 | 0 | 0 | *0* | 0.01 | 0 | 0 | 0 | 0 | 0 | 0 | *0* | *0.0004* |
| A*26~B*18~C*07:01/06/18/52 | 0 | 0.002 | *0* | 0 | 0 | 0.0038 | 0 | 0.0103 | 0.0133 | 0 | *0* | *0.0028* |
| A*26~B*38~C*12:03 | 0 | 0.0097 | *0.0097* | 0.02 | 0.0017 | 0.0019 | 0.0118 | 0.0046 | 0.0133 | 0 | *0.0017* | *0.0088* |
| A*26~B*44~C*05:01/03 | 0.018 | 0.0035 | *0* | 0 | 0 | 0 | 0.002 | 0 | 0 | 0 | *0* | *0.003* |
| A*26~B*51~C*01:02 | 0 | 0 | *0* | 0 | 0 | 0.0152 | 0 | 0 | 0 | 0.0068 | *0* | *0* |
| A*29~B*44~C*16:01 | 0.0273 | 0.0169 | *0.013* | 0.02 | 0.0208 | 0.0303 | 0.0272 | 0.0417 | 0.0067 | 0.0203 | *0.0132* | *0.0199* |
| A*29~B*45~C*06:02 | 0 | 0 | *0* | 0.01 | 0 | 0 | 0 | 0 | 0 | 0 | *0* | *0.0003* |
| A*30~B*13~C*06:02 | 0.0045 | 0.0129 | *0* | 0.01 | 0.0156 | 0 | 0.0079 | 0.0208 | 0.02 | 0.0203 | *0.0132* | *0.0131* |
| A*30~B*13~C*06unspecified | 0.0045 | 0 | *0* | 0.01 | 0 | 0 | 0 | 0 | 0 | 0 | *0* | *0* |
| A*30~B*18~C*05:01/03 | 0 | 0.0071 | *0* | 0.03 | 0 | 0 | 0.002 | 0 | 0 | 0.0068 | *0.0066* | *0.006* |
| HAPLOTYPES | AA (n=55) | BE (n=561) | BS (n=77) | GE (n=50) | GR (n=96) | LG (n=66) | LS (n=127) | LU (n=72) | SG (n=75) | SI (n=74) | ZH (n=151) | All (n=1404) |
| A*30~B*49~C*07:01/06/18/52 | 0.0011 | 0.0039 | *0* | 0 | 0 | 0 | 0 | 0.0139 | 0 | 0 | *0.0003* | *0.0029* |
| A*31~B*35~C*04:01/09N/28/30 | 0.0023 | 0.0018 | *0* | 0.02 | 0 | 0 | 0 | 0 | 0 | 0 | *0.0033* | *0.0034* |
| A*31~B*39~C*12:03 | 0 | 0.0018 | *0* | 0 | 0 | 0 | 0.0118 | 0 | 0 | 0 | *0* | *0.0019* |
| A*31~B*40~C*03:04 | 0 | 0.0058 | *0.013* | 0.0002 | 0.0021 | 0 | 0.001 | 0 | 0 | 0.0051 | *0.0166* | *0.0085* |
| A*31~B*44~C*05:01/03 | 0 | 0 | *0* | 0 | 0.0052 | 0 | 0 | 0 | 0.0044 | 0.0203 | *0.0033* | *0.0019* |
| A*32~B*14~C*08:02 | 0 | 0 | *0* | 0.01 | 0.0138 | 0 | 0.0039 | 0 | 0 | 0.0068 | *0* | *0.0017* |
| A*32~B*15~C*03:03/20N | 0 | 0.0075 | *0* | 0.01 | 0 | 0 | 0 | 0 | 0 | 0 | *0* | *0.0036* |
| A*32~B*15~C*03:04 | 0 | 0 | *0* | 0 | 0 | 0 | 0 | 0.0139 | 0 | 0 | *0* | *0* |
| A*32~B*35~C*04:01/09N/28/30 | 0 | 0.0017 | *0* | 0 | 0.0156 | 0 | 0.0079 | 0 | 0.0049 | 0 | *0* | *0.0032* |
| A*32~B*41~C*17 | 0 | 0 | *0* | 0.01 | 0 | 0 | 0 | 0 | 0 | 0 | *0* | *0* |
| A*32~B*44~C*05:01/03 | 0 | 0.0087 | *0.0195* | 0 | 0.0143 | 0 | 0.0118 | 0 | 0 | 0 | *0* | *0.0075* |
| A*32~B*51~C*15:02/13 | 0 | 0.0031 | *0* | 0 | 0.0019 | 0 | 0 | 0 | 0.02 | 0 | *0* | *0.0034* |
| A*32~B*58~C*07:01/06/18/52 | 0 | 0 | *0* | 0.01 | 0 | 0 | 0 | 0 | 0 | 0 | *0* | *0.0004* |
| A*33~B*14~C*08:02 | 0.0091 | 0.0017 | *0* | 0 | 0 | 0.0379 | 0 | 0.0069 | 0 | 0 | *0.0166* | *0.0069* |
| A*33~B*41~C*17 | 0 | 0 | *0* | 0.01 | 0.0052 | 0 | 0 | 0 | 0 | 0 | *0* | *0.0005* |
| A*33~B*55~C*03:03/20N | 0 | 0 | *0.013* | 0 | 0 | 0 | 0 | 0 | 0.0017 | 0 | *0* | *0.0004* |
| A*66~B*18~C*07:01/06/18/52 | 0 | 0 | *0* | 0 | 0 | 0 | 0 | 0.0139 | 0 | 0 | *0* | *0* |
| A*66~B*41~C*17 | 0 | 0.0027 | *0* | 0.01 | 0 | 0 | 0 | 0 | 0 | 0.0068 | *0* | *0.0018* |
| A*68~B*18~C*05:01/03 | 0 | 0 | *0* | 0 | 0 | 0 | 0 | 0.0139 | 0 | 0 | *0* | *0* |
| A*68~B*35~blank | 0 | 0 | *0.013* | 0 | 0 | 0 | 0 | 0 | 0 | 0 | *0* | *0* |
| A*68~B*35~C*04:01/09N/28/30 | 0.0091 | 0.0032 | *0* | 0 | 0.0083 | 0.0025 | 0 | 0 | 0 | 0 | *0.0166* | *0.006* |
| A*68~B*40~C*03:04 | 0 | 0.0056 | *0* | 0 | 0 | 0 | 0 | 0 | 0.0133 | 0 | *0.0099* | *0.0046* |
| A*68~B*44~C*07:04/11 | 0 | 0.0039 | *0.0065* | 0 | 0.0052 | 0.0008 | 0 | 0 | 0 | 0.0203 | *0* | *0.0041* |
| A*68~B*51~C*07:01/06/18/52 | 0 | 0 | *0* | 0 | 0 | 0.0152 | 0 | 0 | 0 | 0 | *0* | *0* |
| A*68~B*51~C*15:02/13 | 0.0011 | 0.003 | *0.0008* | 0.01 | 0 | 0 | 0.0013 | 0 | 0 | 0.0203 | *0.0017* | *0.003* |
| A*68~B*53~C*04:01/09N/28/30 | 0 | 0.0018 | *0.0065* | 0 | 0.0042 | 0 | 0 | 0.0069 | 0 | 0 | *0.0125* | *0.0034* |
| blank~B*13~C*06:02 | 0 | 0 | *0* | 0 | 0.0104 | 0.0005 | 0 | 0 | 0 | 0 | *0* | *0* |
| blank~B*15~C*03:03/20N | 0 | 0 | *0.0156* | 0 | 0.0065 | 0 | 0 | 0 | 0 | 0 | *0* | *0* |
| blank~B*37~C*06:02 | 0 | 0 | *0* | 0 | 0.0133 | 0.0014 | 0 | 0 | 0 | 0 | *0* | *0* |
| blank~B*44~C*05:01/03 | 0 | 0.0017 | *0* | 0 | 0.0102 | 0 | 0 | 0 | 0 | 0 | *0* | *0* |
| blank~B*55~C*03:03/20N | 0 | 0 | *0* | 0 | 0 | 0 | 0 | 0 | 0.01 | 0 | *0* | *0* |

**HLA-A-B-DRB1**

| HAPLOTYPES | AA (n=84) | BE (n=672) | BS (n=113) | GE (n=84) | GR (n=208) | LG (n=109) | LS (n=357) | LU (n=114) | SG (n=98) | SI (n=131) | ZH (n=233) | All (n=2203) |
| --- | --- | --- | --- | --- | --- | --- | --- | --- | --- | --- | --- | --- |
| A*01~B*08~DRB1*03 | 0.0417 | 0.0461 | *0.0216* | 0.0417 | 0.0288 | 0.0356 | 0.0513 | 0.0259 | 0.0459 | 0.0305 | *0.044* | *0.04* |
| A*01~B*08~DRB1*15 | 0 | 0.0102 | *0* | 0 | 0 | 0 | 0.0032 | 0 | 0 | 0 | *0.0058* | *0.0052* |
| A*01~B*15~DRB1*13 | 0 | 0 | *0.0088* | 0.006 | 0.0024 | 0 | 0 | 0 | 0.0102 | 0 | *0* | *0.0011* |
| A*01~B*35~blank | 0.0119 | 0 | *0* | 0 | 0 | 0 | 0 | 0 | 0 | 0 | *0* | *0* |
| A*01~B*35~DRB1*11 | 0 | 0.0008 | *0.0118* | 0.0179 | 0 | 0.0092 | 0 | 0 | 0 | 0 | *0* | *0.0009* |
| A*01~B*40~DRB1*04 | 0 | 0.0007 | *0* | 0 | 0 | 0 | 0 | 0 | 0 | 0.0153 | *0* | *0.0008* |
| A*01~B*57~DRB1*07 | 0.0119 | 0.0039 | *0.0133* | 0.006 | 0.0024 | 0.0092 | 0.0153 | 0.0088 | 0.0255 | 0.0038 | *0.0105* | *0.0095* |
| A*01~B*57~DRB1*13 | 0 | 0.0039 | *0* | 0 | 0 | 0 | 0.0028 | 0 | 0.0153 | 0 | *0* | *0.0027* |
| A*01~blank~DRB1*14 | 0 | 0 | *0* | 0.0119 | 0 | 0 | 0 | 0 | 0 | 0 | *0* | *0* |
| A*02~B*07~DRB1*03 | 0 | 0 | *0* | 0 | 0 | 0 | 0 | 0 | 0.0153 | 0 | *0* | *0.0018* |
| A*02~B*07~DRB1*15 | 0.0138 | 0.0156 | *0.0133* | 0.006 | 0 | 0 | 0.0196 | 0.0219 | 0.0383 | 0.0053 | *0.0193* | *0.014* |
| A*02~B*08~DRB1*03 | 0 | 0.0105 | *0* | 0 | 0.0062 | 0.0183 | 0.0106 | 0.0048 | 0.0204 | 0.0038 | *0.0022* | *0.0082* |
| A*02~B*13~DRB1*07 | 0.0119 | 0.0056 | *0.0065* | 0 | 0.0025 | 0 | 0.0042 | 0 | 0 | 0 | *0.0107* | *0.0047* |
| A*02~B*14~DRB1*01 | 0 | 0.0015 | *0* | 0.0119 | 0 | 0.0046 | 0 | 0 | 0 | 0 | *0* | *0.0013* |
| A*02~B*14~DRB1*07 | 0 | 0 | *0.0107* | 0 | 0 | 0 | 0.0031 | 0 | 0 | 0 | *0* | *0.0017* |
| A*02~B*15~DRB1*01 | 0 | 0.0027 | *0* | 0 | 0 | 0.0138 | 0 | 0.0169 | 0 | 0 | *0* | *0.0015* |
| A*02~B*15~DRB1*04 | 0 | 0.0117 | *0.0111* | 0.006 | 0.0144 | 0 | 0.0042 | 0 | 0.0102 | 0 | *0.0027* | *0.0077* |
| A*02~B*15~DRB1*08 | 0.0179 | 0.001 | *0* | 0.006 | 0 | 0 | 0 | 0 | 0 | 0.0024 | *0* | *0.0023* |
| A*02~B*15~DRB1*11 | 0 | 0.0014 | *0.0133* | 0.0179 | 0.0126 | 0.0183 | 0.0084 | 0 | 0 | 0.0212 | *0* | *0.0066* |
| A*02~B*15~DRB1*13 | 0.0296 | 0.0132 | *0.0088* | 0 | 0.0095 | 0 | 0.0084 | 0.0175 | 0.0204 | 0 | *0.021* | *0.0112* |
| A*02~B*15~DRB1*15 | 0 | 0.0038 | *0* | 0.006 | 0.0025 | 0 | 0.0042 | 0.0132 | 0 | 0.0095 | *0.0055* | *0.0031* |
| A*02~B*27~DRB1*01 | 0 | 0.0049 | *0* | 0 | 0.0024 | 0 | 0.007 | 0.0132 | 0 | 0 | *0* | *0.004* |
| A*02~B*35~DRB1*01 | 0 | 0.007 | *0* | 0 | 0.0111 | 0 | 0 | 0 | 0 | 0.0038 | *0* | *0.004* |
| A*02~B*35~DRB1*07 | 0 | 0.0022 | *0.0177* | 0.0119 | 0 | 0 | 0.0014 | 0 | 0 | 0 | *0* | *0.0019* |
| A*02~B*35~DRB1*08 | 0.0119 | 0.0036 | *0.0177* | 0 | 0 | 0.0046 | 0 | 0 | 0 | 0.0038 | *0* | *0.0037* |
| A*02~B*35~DRB1*13 | 0.003 | 0 | *0* | 0 | 0 | 0 | 0.0014 | 0 | 0 | 0.0153 | *0* | *0.0018* |
| A*02~B*38~DRB1*01 | 0 | 0 | *0* | 0 | 0 | 0 | 0 | 0 | 0.0102 | 0 | *0* | *0.0002* |
| A*02~B*40~blank | 0.0138 | 0 | *0* | 0 | 0 | 0 | 0 | 0 | 0 | 0 | *0* | *0* |
| A*02~B*40~DRB1*11 | 0 | 0.0013 | *0.0088* | 0 | 0 | 0 | 0 | 0 | 0 | 0.0115 | *0* | *0.0026* |
| A*02~B*40~DRB1*13 | 0.0128 | 0.0119 | *0.0177* | 0.0119 | 0 | 0 | 0.0083 | 0 | 0.0306 | 0.0153 | *0.012* | *0.0112* |
| A*02~B*40~DRB1*16 | 0 | 0.0015 | *0* | 0.0119 | 0 | 0 | 0 | 0 | 0 | 0.0019 | *0* | *0.0009* |
| A*02~B*44~DRB1*01 | 0.0179 | 0.0032 | *0* | 0.0021 | 0.0127 | 0 | 0 | 0 | 0.0102 | 0 | *0* | *0.0045* |
| HAPLOTYPES | AA (n=84) | BE (n=672) | BS (n=113) | GE (n=84) | GR (n=208) | LG (n=109) | LS (n=357) | LU (n=114) | SG (n=98) | SI (n=131) | ZH (n=233) | All (n=2203) |
| A*02~B*44~DRB1*04 | 0 | 0.0154 | *0.0133* | 0 | 0.0216 | 0.0183 | 0.0159 | 0.0041 | 0.0232 | 0.0191 | *0.0172* | *0.0162* |
| A*02~B*44~DRB1*07 | 0 | 0.0079 | *0* | 0 | 0.005 | 0 | 0.0074 | 0.0419 | 0.0153 | 0.0153 | *0.0025* | *0.0052* |
| A*02~B*44~DRB1*11 | 0 | 0.0058 | *0.0044* | 0.0238 | 0 | 0 | 0.0097 | 0 | 0 | 0 | *0.0035* | *0.0074* |
| A*02~B*44~DRB1*15 | 0.0397 | 0 | *0* | 0 | 0.002 | 0 | 0.0044 | 0 | 0 | 0 | *0* | *0.0018* |
| A*02~B*50~DRB1*07 | 0.0179 | 0.0037 | *0* | 0.0119 | 0.0096 | 0 | 0.0084 | 0 | 0 | 0 | *0.0043* | *0.0068* |
| A*02~B*51~DRB1*04 | 0 | 0.0089 | *0.0044* | 0 | 0 | 0 | 0.0028 | 0 | 0 | 0.0153 | *0.0168* | *0.0042* |
| A*02~B*51~DRB1*07 | 0 | 0 | *0* | 0.0119 | 0.0048 | 0 | 0 | 0 | 0 | 0 | *0* | *0* |
| A*02~B*51~DRB1*08 | 0 | 0.0022 | *0* | 0.006 | 0.0051 | 0.0275 | 0 | 0 | 0.0136 | 0 | *0.0078* | *0.0048* |
| A*02~B*51~DRB1*11 | 0.0417 | 0.0095 | *0* | 0.0119 | 0.0409 | 0.0459 | 0.0168 | 0.0044 | 0 | 0.0191 | *0.0201* | *0.0139* |
| A*02~B*51~DRB1*13 | 0.0286 | 0.0127 | *0.0177* | 0 | 0.0107 | 0.0026 | 0.0003 | 0.0102 | 0 | 0.0203 | *0* | *0.0088* |
| A*02~B*51~DRB1*16 | 0 | 0.0008 | *0* | 0.0051 | 0 | 0.0138 | 0.0042 | 0 | 0 | 0.001 | *0* | *0.0024* |
| A*02~B*53~DRB1*13 | 0 | 0 | *0* | 0.0179 | 0 | 0 | 0.001 | 0 | 0.0039 | 0 | *0* | *0* |
| A*02~B*57~DRB1*01 | 0 | 0 | *0.0133* | 0 | 0 | 0 | 0 | 0 | 0 | 0 | *0* | *0* |
| A*02~B*57~DRB1*07 | 0 | 0.0059 | *0* | 0.0015 | 0.012 | 0 | 0.0048 | 0.0011 | 0 | 0 | *0* | *0.0055* |
| A*02~B*58~DRB1*03 | 0 | 0 | *0* | 0.0119 | 0 | 0 | 0.0028 | 0 | 0 | 0 | *0* | *0.0008* |
| A*02~blank~DRB1*07 | 0.0119 | 0 | *0* | 0 | 0 | 0 | 0.0017 | 0 | 0 | 0 | *0* | *0.0007* |
| A*03~B*07~blank | 0 | 0 | *0* | 0 | 0 | 0 | 0 | 0 | 0.0102 | 0 | *0* | *0* |
| A*03~B*07~DRB1*04 | 0 | 0.0109 | *0* | 0 | 0 | 0 | 0 | 0 | 0 | 0 | *0* | *0.0043* |
| A*03~B*07~DRB1*08 | 0 | 0 | *0* | 0 | 0.0072 | 0 | 0 | 0.0203 | 0 | 0.0057 | *0* | *0.0026* |
| A*03~B*07~DRB1*11 | 0 | 0.0096 | *0* | 0 | 0 | 0.0092 | 0.007 | 0 | 0.0102 | 0 | *0.0025* | *0.0046* |
| A*03~B*07~DRB1*13 | 0 | 0.0053 | *0* | 0 | 0.0063 | 0 | 0.0131 | 0 | 0 | 0.0038 | *0.0064* | *0.0053* |
| A*03~B*07~DRB1*15 | 0.0238 | 0.0371 | *0.0265* | 0 | 0.0327 | 0.0356 | 0.0271 | 0.03 | 0.0128 | 0.0228 | *0.0279* | *0.0281* |
| A*03~B*15~DRB1*03 | 0 | 0 | *0* | 0 | 0 | 0 | 0 | 0 | 0 | 0.0153 | *0* | *0* |
| A*03~B*35~DRB1*01 | 0.006 | 0.0131 | *0.0171* | 0.0119 | 0 | 0.0057 | 0.0116 | 0.0163 | 0 | 0 | *0.0149* | *0.0108* |
| A*03~B*35~DRB1*04 | 0 | 0 | *0* | 0 | 0.0096 | 0 | 0.0084 | 0.0132 | 0 | 0 | *0.0032* | *0.0036* |
| A*03~B*35~DRB1*11 | 0.0238 | 0.0024 | *0* | 0 | 0 | 0 | 0 | 0.015 | 0 | 0 | *0.0084* | *0.003* |
| A*03~B*35~DRB1*14 | 0 | 0.0035 | *0* | 0 | 0 | 0 | 0 | 0 | 0 | 0.0115 | *0* | *0.0021* |
| A*03~B*44~DRB1*07 | 0 | 0.0011 | *0* | 0.006 | 0 | 0 | 0.0056 | 0 | 0 | 0.0115 | *0* | *0.0012* |
| A*03~B*44~DRB1*13 | 0 | 0.0011 | *0* | 0 | 0.0048 | 0.0092 | 0 | 0.0175 | 0 | 0 | *0.0107* | *0.0048* |
| A*03~B*51~DRB1*01 | 0.0119 | 0.0013 | *0* | 0.0179 | 0 | 0 | 0 | 0 | 0 | 0 | *0.0043* | *0.0017* |
| A*03~B*51~DRB1*14 | 0 | 0 | *0* | 0 | 0 | 0.0039 | 0 | 0 | 0.0102 | 0 | *0* | *0* |
| A*03~B*57~DRB1*07 | 0 | 0 | *0* | 0.0119 | 0 | 0.0046 | 0 | 0 | 0 | 0 | *0* | *0* |
| A*11~B*08~DRB1*03 | 0 | 0.0015 | *0* | 0.0119 | 0 | 0 | 0.0038 | 0.0044 | 0 | 0 | *0* | *0.0019* |
| A*11~B*35~DRB1*01 | 0.0179 | 0.0076 | *0.0265* | 0 | 0.0117 | 0.0046 | 0 | 0 | 0 | 0 | *0.0091* | *0.0071* |
| HAPLOTYPES | AA (n=84) | BE (n=672) | BS (n=113) | GE (n=84) | GR (n=208) | LG (n=109) | LS (n=357) | LU (n=114) | SG (n=98) | SI (n=131) | ZH (n=233) | All (n=2203) |
| A*11~B*35~DRB1*04 | 0 | 0.0015 | *0* | 0.0119 | 0.0024 | 0.0092 | 0 | 0 | 0.0102 | 0 | *0* | *0.0022* |
| A*11~B*35~DRB1*07 | 0 | 0.0022 | *0* | 0 | 0 | 0.0046 | 0 | 0.0132 | 0 | 0.0057 | *0* | *0.0014* |
| A*11~B*44~DRB1*07 | 0 | 0 | *0* | 0 | 0 | 0 | 0.0028 | 0 | 0.0153 | 0.0038 | *0* | *0.0012* |
| A*11~B*51~DRB1*04 | 0.0179 | 0 | *0* | 0 | 0 | 0.0046 | 0.0054 | 0 | 0 | 0 | *0.0043* | *0.002* |
| A*11~B*51~DRB1*08 | 0.0119 | 0 | *0* | 0.0015 | 0 | 0 | 0 | 0 | 0 | 0 | *0* | *0.0007* |
| A*11~B*55~DRB1*11 | 0 | 0.0007 | *0* | 0 | 0.0016 | 0 | 0 | 0 | 0.0102 | 0 | *0* | *0.0008* |
| A*11~B*55~DRB1*14 | 0 | 0.0037 | *0.0088* | 0 | 0 | 0 | 0 | 0.0044 | 0 | 0.0115 | *0* | *0.003* |
| A*23~B*44~DRB1*07 | 0.0099 | 0.0088 | *0.0044* | 0 | 0 | 0.0046 | 0.0162 | 0.0058 | 0 | 0.0076 | *0.0129* | *0.0096* |
| A*24~B*07~DRB1*04 | 0 | 0 | *0* | 0 | 0 | 0.0112 | 0 | 0 | 0.0102 | 0 | *0.0021* | *0.0022* |
| A*24~B*07~DRB1*11 | 0.01 | 0.0021 | *0* | 0 | 0 | 0 | 0 | 0 | 0 | 0 | *0* | *0.0008* |
| A*24~B*07~DRB1*15 | 0 | 0.0094 | *0* | 0.0298 | 0.0025 | 0 | 0.0079 | 0 | 0 | 0.0028 | *0.0169* | *0.0076* |
| A*24~B*08~DRB1*03 | 0 | 0.0007 | *0.0133* | 0 | 0.012 | 0 | 0 | 0.0044 | 0 | 0.0076 | *0* | *0.0043* |
| A*24~B*14~DRB1*01 | 0 | 0 | *0* | 0.006 | 0 | 0 | 0 | 0 | 0 | 0.0229 | *0* | *0.0018* |
| A*24~B*14~DRB1*04 | 0 | 0.0015 | *0.0133* | 0 | 0 | 0 | 0 | 0 | 0 | 0 | *0* | *0.0004* |
| A*24~B*15~DRB1*01 | 0 | 0 | *0* | 0 | 0 | 0 | 0 | 0.0044 | 0 | 0.0148 | *0.0011* | *0.0024* |
| A*24~B*35~DRB1*01 | 0 | 0.0038 | *0* | 0.0119 | 0.0024 | 0 | 0.0014 | 0 | 0.0013 | 0.0043 | *0* | *0.0023* |
| A*24~B*35~DRB1*04 | 0 | 0 | *0* | 0.006 | 0 | 0 | 0.0028 | 0.0088 | 0.0153 | 0.0038 | *0.0161* | *0.0039* |
| A*24~B*35~DRB1*11 | 0.006 | 0.0072 | *0* | 0 | 0.007 | 0.03 | 0.0084 | 0 | 0.0102 | 0.0186 | *0.0086* | *0.0081* |
| A*24~B*38~DRB1*13 | 0 | 0 | *0.0177* | 0 | 0.0024 | 0 | 0 | 0 | 0 | 0 | *0* | *0.0007* |
| A*24~B*39~DRB1*11 | 0.006 | 0 | *0* | 0.0119 | 0 | 0 | 0.0042 | 0 | 0 | 0 | *0* | *0* |
| A*24~B*44~DRB1*01 | 0 | 0 | *0* | 0.0015 | 0.024 | 0 | 0.0014 | 0 | 0 | 0.0038 | *0* | *0.0022* |
| A*24~B*44~DRB1*03 | 0 | 0 | *0* | 0 | 0 | 0 | 0 | 0.0132 | 0 | 0 | *0* | *0* |
| A*24~B*44~DRB1*07 | 0.006 | 0 | *0.0044* | 0.014 | 0 | 0 | 0 | 0 | 0 | 0 | *0.0041* | *0.0025* |
| A*24~B*44~DRB1*11 | 0.0138 | 0.002 | *0* | 0 | 0.0048 | 0 | 0 | 0 | 0 | 0 | *0* | *0.0004* |
| A*24~B*51~DRB1*01 | 0 | 0 | *0* | 0 | 0 | 0 | 0 | 0 | 0.0153 | 0 | *0.0021* | *0* |
| A*24~B*51~DRB1*11 | 0.006 | 0.0049 | *0.0032* | 0 | 0.0101 | 0.0046 | 0 | 0 | 0.0102 | 0.0038 | *0* | *0.0048* |
| A*24~B*57~DRB1*07 | 0 | 0.0019 | *0.0088* | 0 | 0 | 0.0046 | 0.0028 | 0.0031 | 0.0204 | 0 | *0.0015* | *0.004* |
| A*25~B*18~DRB1*15 | 0 | 0.0045 | *0.0133* | 0 | 0 | 0 | 0 | 0 | 0 | 0 | *0.0064* | *0.0041* |
| A*26~B*07~DRB1*15 | 0 | 0 | *0* | 0.0179 | 0 | 0 | 0 | 0.0025 | 0 | 0 | *0* | *0.0011* |
| A*26~B*35~DRB1*07 | 0 | 0.0008 | *0* | 0.0119 | 0 | 0 | 0 | 0 | 0 | 0 | *0* | *0.0002* |
| A*26~B*38~DRB1*11 | 0 | 0 | *0.0122* | 0 | 0.0024 | 0 | 0.0014 | 0 | 0 | 0.0043 | *0* | *0.0016* |
| A*26~B*51~DRB1*13 | 0 | 0.001 | *0* | 0 | 0.0024 | 0.0011 | 0 | 0 | 0 | 0.0102 | *0.0021* | *0.0014* |
| A*26~B*57~DRB1*07 | 0.0119 | 0 | *0* | 0 | 0 | 0 | 0 | 0.0011 | 0 | 0 | *0* | *0* |
| A*29~B*44~DRB1*07 | 0.0119 | 0.0149 | *0.0177* | 0.0238 | 0.0118 | 0.0092 | 0.0101 | 0.0148 | 0 | 0.0076 | *0.0129* | *0.0145* |
| HAPLOTYPES | AA (n=84) | BE (n=672) | BS (n=113) | GE (n=84) | GR (n=208) | LG (n=109) | LS (n=357) | LU (n=114) | SG (n=98) | SI (n=131) | ZH (n=233) | All (n=2203) |
| A*29~B*44~DRB1*11 | 0 | 0.0022 | *0* | 0 | 0 | 0 | 0 | 0 | 0.0102 | 0 | *0* | *0* |
| A*30~B*13~DRB1*07 | 0.006 | 0.0096 | *0.0044* | 0.006 | 0.0167 | 0 | 0.0084 | 0.0175 | 0.0153 | 0.0115 | *0.0043* | *0.0099* |
| A*30~B*18~DRB1*03 | 0 | 0.0067 | *0* | 0.0179 | 0 | 0.0092 | 0.0014 | 0 | 0 | 0 | *0.0021* | *0.005* |
| A*30~B*18~DRB1*11 | 0 | 0 | *0* | 0.0119 | 0 | 0 | 0 | 0 | 0 | 0 | *0* | *0* |
| A*30~B*44~DRB1*07 | 0.0119 | 0.0015 | *0* | 0 | 0 | 0 | 0 | 0 | 0 | 0 | *0* | *0.001* |
| A*31~B*40~DRB1*04 | 0 | 0.0029 | *0* | 0 | 0.0072 | 0 | 0.0028 | 0 | 0 | 0.0076 | *0.0129* | *0.0047* |
| A*31~B*44~DRB1*08 | 0 | 0 | *0* | 0 | 0 | 0 | 0 | 0 | 0.0102 | 0 | *0* | *0.0008* |
| A*31~B*51~DRB1*11 | 0 | 0.0013 | *0* | 0.0119 | 0 | 0.0046 | 0 | 0 | 0 | 0 | *0* | *0.0013* |
| A*31~B*56~DRB1*15 | 0 | 0 | *0* | 0 | 0 | 0 | 0 | 0 | 0.0102 | 0 | *0* | *0* |
| A*32~B*07~DRB1*15 | 0 | 0 | *0* | 0 | 0 | 0.0046 | 0 | 0 | 0.0102 | 0 | *0* | *0.0004* |
| A*32~B*40~DRB1*11 | 0 | 0.0048 | *0* | 0 | 0.012 | 0 | 0.0036 | 0 | 0 | 0 | *0* | *0.0033* |
| A*33~B*14~DRB1*01 | 0.006 | 0.0009 | *0.0088* | 0 | 0.0048 | 0.0183 | 0.0014 | 0 | 0 | 0 | *0.0127* | *0.0047* |
| A*66~B*15~DRB1*07 | 0.0119 | 0.0007 | *0* | 0 | 0 | 0 | 0 | 0 | 0 | 0 | *0* | *0.0005* |
| A*66~B*41~DRB1*13 | 0.0059 | 0 | *0.0022* | 0.0015 | 0 | 0 | 0 | 0 | 0.0102 | 0 | *0* | *0.002* |
| A*68~B*07~DRB1*13 | 0.0119 | 0.002 | *0* | 0 | 0 | 0 | 0 | 0 | 0 | 0 | *0* | *0* |
| A*68~B*15~DRB1*04 | 0 | 0 | *0* | 0 | 0 | 0 | 0 | 0 | 0.0102 | 0 | *0* | *0.0013* |
| A*68~B*44~DRB1*11 | 0 | 0 | *0* | 0 | 0.0024 | 0 | 0 | 0 | 0 | 0.0102 | *0* | *0.0017* |
| A*68~B*53~DRB1*01 | 0 | 0.0005 | *0* | 0 | 0 | 0.0046 | 0 | 0 | 0.0102 | 0 | *0* | *0.0012* |
| A*68~B*53~DRB1*11 | 0.0119 | 0 | *0* | 0 | 0 | 0 | 0 | 0 | 0 | 0 | *0* | *0.0004* |
| A*80~B*58~DRB1*14 | 0 | 0 | *0.0133* | 0 | 0 | 0 | 0 | 0 | 0 | 0 | *0* | *0.0007* |
| blank~B*15~DRB1*04 | 0 | 0 | *0.0111* | 0 | 0 | 0 | 0 | 0 | 0 | 0 | *0.0027* | *0.0005* |
| blank~B*15~DRB1*13 | 0.0121 | 0 | *0* | 0 | 0 | 0 | 0 | 0 | 0 | 0 | *0.0038* | *0.0001* |
| blank~B*55~DRB1*14 | 0 | 0 | *0* | 0 | 0 | 0 | 0 | 0 | 0.0102 | 0 | *0* | *0* |

**HLA-A-B**

| HAPLOTYPES | AA (n=84) | BE (n=683) | BS (n=113) | GE (n=84) | GR (n=211) | LG (n=109) | LS (n=359) | LU (n=115) | SG (n=98) | SI (n=133) | ZH (n=235) | All (n=2224) |
| --- | --- | --- | --- | --- | --- | --- | --- | --- | --- | --- | --- | --- |
| A*01~B*07 | 0.0089 | 0.0083 | *0* | 0.006 | 0.0084 | 0 | 0.0059 | 0 | 0 | 0.0066 | *0.0107* | *0.0062* |
| A*01~B*08 | 0.0476 | 0.0613 | *0.0337* | 0.0532 | 0.0445 | 0.0459 | 0.0627 | 0.0385 | 0.0505 | 0.0495 | *0.0562* | *0.0539* |
| A*01~B*15 | 0 | 0.0025 | *0.0062* | 0.0078 | 0.0024 | 0 | 0.0066 | 0 | 0.0107 | 0.0079 | *0.0062* | *0.0043* |
| A*01~B*35:01/40N/42/57/94 | 0 | 0.0055 | *0.0144* | 0.0089 | 0 | 0 | 0.0051 | 0.0031 | 0.0051 | 0 | *0* | *0.004* |
| A*01~B*35:02 | 0 | 0.0007 | *0* | 0 | 0 | 0 | 0 | 0.0122 | 0 | 0 | *0* | *0.0009* |
| A*01~B*37:01 | 0 | 0.0045 | *0.0121* | 0 | 0.0042 | 0 | 0.0068 | 0.005 | 0.0013 | 0.0038 | *0.0043* | *0.0054* |
| A*01~B*38:01 | 0.0179 | 0.002 | *0* | 0 | 0 | 0 | 0 | 0.0022 | 0 | 0 | *0* | *0.0015* |
| A*01~B*40:01/55 | 0 | 0.0015 | *0* | 0 | 0.0016 | 0 | 0 | 0 | 0 | 0.0108 | *0.0043* | *0.0022* |
| A*01~B*51 | 0 | 0.0032 | *0.0133* | 0 | 0 | 0 | 0 | 0 | 0 | 0 | *0* | *0.0017* |
| A*01~B*52:01/07 | 0.0119 | 0.0044 | *0.0044* | 0.006 | 0.0047 | 0.0046 | 0 | 0 | 0.0051 | 0 | *0.0022* | *0.0034* |
| A*01~B*57:01 | 0.0089 | 0.0107 | *0.0082* | 0.0103 | 0.0071 | 0.0138 | 0.0198 | 0.0029 | 0.0459 | 0.022 | *0.0148* | *0.0148* |
| A*02~B*07 | 0.0119 | 0.0276 | *0.0297* | 0.0298 | 0.0313 | 0 | 0.0278 | 0.0349 | 0.0606 | 0.0247 | *0.0306* | *0.0266* |
| A*02~B*08 | 0 | 0.0108 | *0* | 0 | 0.0042 | 0.0367 | 0.0138 | 0.005 | 0.0158 | 0.0178 | *0.0026* | *0.0084* |
| A*02~B*13:02 | 0.0119 | 0.0059 | *0.0106* | 0 | 0.0036 | 0.0039 | 0.0067 | 0.0101 | 0 | 0.0075 | *0* | *0.007* |
| A*02~B*14:02 | 0 | 0.001 | *0* | 0.0119 | 0 | 0 | 0 | 0 | 0 | 0.0075 | *0* | *0.0013* |
| A*02~B*15 | 0.0648 | 0.0369 | *0.0298* | 0.0261 | 0.0458 | 0.0287 | 0.0214 | 0.0506 | 0.0301 | 0.0341 | *0.0378* | *0.0351* |
| A*02~B*18 | 0.0155 | 0.0132 | *0.0193* | 0.0179 | 0.0084 | 0.0265 | 0.0116 | 0 | 0.0102 | 0.0113 | *0.0156* | *0.0134* |
| A*02~B*27:05/13 | 0 | 0.0153 | *0.0051* | 0 | 0.0061 | 0.0046 | 0.0186 | 0.0237 | 0.0036 | 0.0014 | *0.0037* | *0.0117* |
| A*02~B*35:01/40N/42/57/94 | 0.0065 | 0.0119 | *0.0265* | 0 | 0.0035 | 0.0095 | 0.0068 | 0 | 0.0052 | 0 | *0.0031* | *0.0095* |
| A*02~B*35:02 | 0 | 0 | *0* | 0 | 0.0044 | 0 | 0 | 0 | 0 | 0.0132 | *0* | *0* |
| A*02~B*35:03/70 | 0.0196 | 0.0051 | *0* | 0 | 0.0054 | 0.0129 | 0.0068 | 0 | 0.0052 | 0.0198 | *0* | *0.0056* |
| A*02~B*40:01/55 | 0.0286 | 0.0206 | *0.0317* | 0.0152 | 0.0091 | 0.0101 | 0.0253 | 0.0056 | 0.023 | 0.0298 | *0.0391* | *0.0222* |
| A*02~B*44:02/19N/27 | 0.0648 | 0.0398 | *0.0244* | 0.0403 | 0.029 | 0.0301 | 0.0296 | 0.0158 | 0.0538 | 0.0084 | *0.0267* | *0.0322* |
| A*02~B*44:03 | 0 | 0.0099 | *0.0052* | 0 | 0 | 0 | 0.0128 | 0.0274 | 0 | 0.0073 | *0.0061* | *0.0067* |
| A*02~B*49:01 | 0 | 0.0014 | *0.0022* | 0.006 | 0.0053 | 0.0046 | 0 | 0.013 | 0.0102 | 0 | *0.0021* | *0.003* |
| A*02~B*50:01 | 0.0179 | 0.0043 | *0.0088* | 0.0179 | 0.0094 | 0.0229 | 0.0093 | 0 | 0 | 0 | *0* | *0.0089* |
| A*02~B*50:02 | 0 | 0 | *0* | 0 | 0 | 0 | 0 | 0.013 | 0 | 0 | *0* | *0.0003* |
| A*02~B*51 | 0.0701 | 0.037 | *0.0177* | 0.0413 | 0.0702 | 0.084 | 0.0326 | 0.0234 | 0.0162 | 0.0489 | *0.041* | *0.042* |
| A*02~B*52:01/07 | 0.006 | 0.0009 | *0* | 0.0119 | 0 | 0.0047 | 0.001 | 0.0011 | 0 | 0 | *0.0018* | *0.0024* |
| A*02~B*53:01 | 0 | 0 | *0* | 0.0179 | 0 | 0 | 0 | 0 | 0.0061 | 0 | *0* | *0.0007* |
| A*02~B*55 | 0 | 0.0021 | *0* | 0.003 | 0 | 0 | 0.0043 | 0.0043 | 0 | 0 | *0.0156* | *0.0026* |
| A*02~B*57:01 | 0.0042 | 0.0084 | *0.0184* | 0 | 0.0118 | 0 | 0.0066 | 0 | 0 | 0.008 | *0* | *0.0072* |
| HAPLOTYPES | AA (n=84) | BE (n=683) | BS (n=113) | GE (n=84) | GR (n=211) | LG (n=109) | LS (n=359) | LU (n=115) | SG (n=98) | SI (n=133) | ZH (n=235) | All (n=2224) |
| A*02~B*58:01/11 | 0 | 0.0025 | *0* | 0 | 0 | 0 | 0.0111 | 0 | 0.0051 | 0 | *0.0027* | *0.0044* |
| A*02~blank | 0 | 0 | *0.0163* | 0.0059 | 0 | 0 | 0 | 0 | 0.0231 | 0 | *0* | *0* |
| A*03~B*07 | 0.0268 | 0.0651 | *0.0392* | 0 | 0.0615 | 0.055 | 0.0532 | 0.06 | 0.0375 | 0.0326 | *0.0375* | *0.0501* |
| A*03~B*15 | 0 | 0.0037 | *0.0136* | 0.016 | 0.0039 | 0.006 | 0.0052 | 0.0189 | 0.0102 | 0.0177 | *0.0086* | *0.0078* |
| A*03~B*18 | 0.011 | 0.0075 | *0.0088* | 0 | 0.0024 | 0.0065 | 0.0084 | 0 | 0 | 0 | *0* | *0.0054* |
| A*03~B*35:01/40N/42/57/94 | 0.0069 | 0.0209 | *0.0164* | 0 | 0.0067 | 0 | 0.0148 | 0.0151 | 0 | 0.0084 | *0.016* | *0.0149* |
| A*03~B*35:03/70 | 0.0089 | 0.0032 | *0* | 0.0108 | 0 | 0.0092 | 0.0115 | 0.0157 | 0 | 0.0063 | *0.0218* | *0.009* |
| A*03~B*35:36 | 0 | 0 | *0* | 0.0108 | 0 | 0.0092 | 0 | 0.0157 | 0 | 0.0063 | *0* | *0* |
| A*03~B*40:01/55 | 0 | 0.0051 | *0.0133* | 0 | 0.0054 | 0.0082 | 0 | 0 | 0 | 0 | *0.0046* | *0.0031* |
| A*03~B*44:02/19N/27 | 0 | 0.0095 | *0* | 0 | 0.0059 | 0.0106 | 0.0025 | 0.011 | 0 | 0.0038 | *0.0106* | *0.0059* |
| A*03~B*44:03 | 0 | 0.0003 | *0* | 0.006 | 0 | 0 | 0.0045 | 0.0045 | 0.0051 | 0.0113 | *0* | *0.0015* |
| A*03~B*47:01 | 0 | 0.0029 | *0* | 0 | 0.0024 | 0 | 0 | 0 | 0 | 0.0113 | *0* | *0.0018* |
| A*03~B*51 | 0.0298 | 0.0065 | *0* | 0.0149 | 0.0109 | 0.0114 | 0.006 | 0.0046 | 0.0186 | 0 | *0.017* | *0.0083* |
| A*03~B*55 | 0 | 0.001 | *0.0044* | 0.0119 | 0 | 0.0033 | 0 | 0.0057 | 0 | 0.0038 | *0* | *0.0023* |
| A*03~blank | 0 | 0.0016 | *0* | 0.0172 | 0 | 0 | 0 | 0 | 0 | 0 | *0* | *0.0008* |
| A*11~B*07 | 0 | 0.0047 | *0.0047* | 0 | 0.0025 | 0 | 0.002 | 0.0144 | 0 | 0 | *0* | *0.0037* |
| A*11~B*35:01/40N/42/57/94 | 0 | 0.0172 | *0.0262* | 0.023 | 0.0247 | 0.0203 | 0.0098 | 0.0217 | 0 | 0.015 | *0.0118* | *0.0169* |
| A*11~B*35:03/70 | 0.0215 | 0.0014 | *0* | 0 | 0.0017 | 0 | 0.0012 | 0 | 0.0102 | 0 | *0* | *0.0019* |
| A*11~B*44:02/19N/27 | 0 | 0.0042 | *0* | 0.0119 | 0 | 0.0005 | 0.009 | 0 | 0.0102 | 0.0084 | *0* | *0.0036* |
| A*11~B*51 | 0.0221 | 0.0096 | *0* | 0 | 0.0094 | 0.0136 | 0.0102 | 0 | 0 | 0.0104 | *0.0086* | *0.0084* |
| A*11~B*55 | 0.006 | 0.0064 | *0.0088* | 0 | 0.0024 | 0 | 0.0053 | 0.003 | 0.0128 | 0.0075 | *0.0034* | *0.006* |
| A*23~B*44:03 | 0.0179 | 0.0094 | *0.0133* | 0 | 0.0095 | 0.0006 | 0.0211 | 0.0143 | 0 | 0.0226 | *0.0139* | *0.0129* |
| A*23~B*49:01 | 0 | 0.01 | *0* | 0 | 0.0024 | 0.0092 | 0.0055 | 0 | 0 | 0 | *0.0043* | *0.0056* |
| A*23~B*51 | 0 | 0.0007 | *0* | 0 | 0 | 0 | 0.0026 | 0 | 0.0102 | 0 | *0.0035* | *0.001* |
| A*24~B*07 | 0.0179 | 0.0154 | *0* | 0.0179 | 0 | 0.0162 | 0.0114 | 0.0124 | 0.0105 | 0 | *0.0196* | *0.0148* |
| A*24~B*08 | 0 | 0.001 | *0.0177* | 0 | 0.016 | 0 | 0.0062 | 0.013 | 0 | 0.0079 | *0* | *0.0056* |
| A*24~B*14:02 | 0 | 0.0027 | *0.005* | 0 | 0 | 0 | 0 | 0 | 0 | 0.0226 | *0* | *0.0023* |
| A*24~B*15 | 0 | 0.0132 | *0.0198* | 0 | 0.0077 | 0.0209 | 0 | 0 | 0 | 0.0235 | *0.0188* | *0.0111* |
| A*24~B*18 | 0.0069 | 0.0093 | *0* | 0.0119 | 0.0115 | 0.0073 | 0.0022 | 0.0043 | 0 | 0 | *0* | *0.0062* |
| A*24~B*27:02 | 0 | 0.0016 | *0* | 0 | 0 | 0 | 0 | 0 | 0.0102 | 0 | *0* | *0.0004* |
| A*24~B*35:01/40N/42/57/94 | 0.0229 | 0.0066 | *0* | 0.0119 | 0 | 0.0224 | 0 | 0 | 0 | 0.0102 | *0.0207* | *0.0064* |
| A*24~B*35:02 | 0 | 0.0065 | *0.0088* | 0 | 0.0029 | 0.0077 | 0 | 0 | 0.0152 | 0 | *0* | *0.005* |
| A*24~B*35:03/70 | 0 | 0.0046 | *0.0044* | 0.0119 | 0 | 0 | 0 | 0.0043 | 0.0152 | 0.0051 | *0* | *0.0046* |
| A*24~B*38:01 | 0.0119 | 0 | *0.0133* | 0.0014 | 0.0012 | 0 | 0.0017 | 0 | 0 | 0 | *0.006* | *0.0031* |
| HAPLOTYPES | AA (n=84) | BE (n=683) | BS (n=113) | GE (n=84) | GR (n=211) | LG (n=109) | LS (n=359) | LU (n=115) | SG (n=98) | SI (n=133) | ZH (n=235) | All (n=2224) |
| A*24~B*39:06 | 0.006 | 0.0021 | *0* | 0.0119 | 0.0024 | 0 | 0.0028 | 0.0043 | 0 | 0.0075 | *0.0043* | *0.0025* |
| A*24~B*44:02/19N/27 | 0 | 0 | *0* | 0 | 0.0188 | 0 | 0 | 0.0021 | 0 | 0.0047 | *0.0078* | *0.0029* |
| A*24~B*44:03 | 0 | 0 | *0.0044* | 0.0159 | 0.0125 | 0.0138 | 0.0031 | 0 | 0 | 0 | *0.0054* | *0.0039* |
| A*24~B*51 | 0 | 0.0128 | *0* | 0 | 0.0161 | 0.0131 | 0.0114 | 0 | 0.0255 | 0 | *0* | *0.0111* |
| A*24~B*57:01 | 0 | 0.0034 | *0.0088* | 0 | 0 | 0.0138 | 0.0063 | 0.0043 | 0.0204 | 0 | *0.0021* | *0.0041* |
| A*25:01~B*18 | 0 | 0.0101 | *0.0177* | 0 | 0.0036 | 0.0092 | 0.0055 | 0.0087 | 0 | 0.0019 | *0.0082* | *0.0081* |
| A*26~B*07 | 0 | 0.0042 | *0* | 0.0238 | 0 | 0 | 0 | 0 | 0 | 0 | *0* | *0.0023* |
| A*26~B*15 | 0 | 0 | *0* | 0 | 0 | 0 | 0.0109 | 0 | 0 | 0 | *0.0043* | *0.0016* |
| A*26~B*18 | 0 | 0.0028 | *0.0044* | 0 | 0.0024 | 0.0031 | 0.0028 | 0.013 | 0.0102 | 0.0038 | *0* | *0.0031* |
| A*26~B*27:05/13 | 0.001 | 0.0036 | *0* | 0.0019 | 0 | 0 | 0.0014 | 0.004 | 0.0102 | 0 | *0* | *0.0032* |
| A*26~B*38:01 | 0 | 0.0087 | *0.0265* | 0.0119 | 0.0036 | 0.0138 | 0.0135 | 0.0017 | 0.0141 | 0.0038 | *0.0021* | *0.0099* |
| A*26~B*44:02/19N/27 | 0.0119 | 0.0041 | *0* | 0 | 0.0024 | 0 | 0 | 0 | 0 | 0 | *0* | *0.0025* |
| A*26~B*44:03 | 0 | 0.002 | *0* | 0.0119 | 0 | 0.0046 | 0 | 0 | 0 | 0 | *0.0043* | *0.0021* |
| A*26~B*51 | 0 | 0 | *0* | 0 | 0.004 | 0.0046 | 0.0019 | 0.0072 | 0 | 0.015 | *0* | *0.0031* |
| A*26~B*55 | 0 | 0.0021 | *0* | 0 | 0.0118 | 0.0046 | 0 | 0 | 0 | 0.0038 | *0* | *0.0023* |
| A*26~B*57:01 | 0.0119 | 0 | *0* | 0 | 0 | 0 | 0 | 0.0043 | 0 | 0 | *0* | *0.0004* |
| A*29~B*44:03 | 0.0238 | 0.0149 | *0.0177* | 0.0238 | 0.0166 | 0.0183 | 0.0172 | 0.0326 | 0.0153 | 0.015 | *0.0191* | *0.0179* |
| A*30:01/24~B*13:02 | 0.0015 | 0.0129 | *0.0133* | 0.003 | 0.0047 | 0.0011 | 0.0077 | 0.0174 | 0.0153 | 0.0113 | *0.0032* | *0.0122* |
| A*30:02~B*18 | 0 | 0.0073 | *0* | 0.0238 | 0 | 0.0033 | 0.0005 | 0 | 0 | 0.0025 | *0.0064* | *0.0051* |
| A*30:02~B*58:01/11 | 0 | 0 | *0* | 0.0119 | 0 | 0 | 0 | 0 | 0 | 0 | *0.0007* | *0.0005* |
| A*31~B*40:01/55 | 0 | 0.0081 | *0.0096* | 0 | 0.0164 | 0 | 0.006 | 0 | 0 | 0.0102 | *0.0082* | *0.0076* |
| A*31~B*44:02/19N/27 | 0 | 0 | *0.0221* | 0 | 0.0095 | 0 | 0.0017 | 0 | 0.0153 | 0.0158 | *0.0002* | *0.0039* |
| A*31~B*51 | 0 | 0.0082 | *0* | 0.006 | 0.0048 | 0.0183 | 0.0021 | 0 | 0.0102 | 0.0116 | *0.0085* | *0.0071* |
| A*31~B*56 | 0 | 0 | *0* | 0 | 0 | 0 | 0.0014 | 0 | 0.0102 | 0 | *0* | *0.0007* |
| A*32:01~B*07 | 0 | 0 | *0* | 0 | 0 | 0.0097 | 0 | 0 | 0.0102 | 0 | *0* | *0* |
| A*32:01~B*15 | 0.0119 | 0.0081 | *0.0058* | 0.006 | 0 | 0.0087 | 0 | 0.013 | 0 | 0 | *0* | *0.0047* |
| A*32:01~B*27:05/13 | 0 | 0.0011 | *0* | 0 | 0.0102 | 0.0005 | 0 | 0 | 0 | 0.01 | *0.0029* | *0.0022* |
| A*32:01~B*35:03/70 | 0 | 0 | *0* | 0.0004 | 0.0036 | 0 | 0 | 0 | 0.0102 | 0 | *0* | *0.0016* |
| A*32:01~B*40:01/55 | 0 | 0.0027 | *0.0133* | 0 | 0.0033 | 0 | 0.0045 | 0 | 0.0026 | 0 | *0* | *0.0026* |
| A*32:01~B*40:02/B*40:56 | 0 | 0.008 | *0* | 0.006 | 0.0095 | 0.0046 | 0.011 | 0.0043 | 0.0051 | 0 | *0.0074* | *0.0073* |
| A*32:01~B*51 | 0 | 0.0036 | *0.0044* | 0 | 0 | 0 | 0.0045 | 0.0043 | 0 | 0.0226 | *0.0085* | *0.0049* |
| A*33~B*14:02 | 0.006 | 0.0022 | *0.0088* | 0.0119 | 0.0047 | 0.0229 | 0.0042 | 0.0043 | 0 | 0 | *0.0149* | *0.006* |
| A*66:01/04~B*41:02 | 0.003 | 0.0022 | *0.0044* | 0.0119 | 0 | 0 | 0 | 0 | 0.0102 | 0.0038 | *0* | *0.0022* |
| A*68~B*08 | 0 | 0 | *0* | 0.0119 | 0 | 0 | 0 | 0 | 0 | 0 | *0* | *0.0005* |
| HAPLOTYPES | AA (n=84) | BE (n=683) | BS (n=113) | GE (n=84) | GR (n=211) | LG (n=109) | LS (n=359) | LU (n=115) | SG (n=98) | SI (n=133) | ZH (n=235) | All (n=2224) |
| A*68~B*18 | 0 | 0.0004 | *0* | 0 | 0 | 0.007 | 0.0021 | 0.013 | 0 | 0 | *0* | *0.0017* |
| A*68~B*40:01/55 | 0.006 | 0.0051 | *0.0044* | 0 | 0 | 0.0046 | 0 | 0 | 0.0102 | 0 | *0.0102* | *0.0036* |
| A*68~B*44:02/19N/27 | 0 | 0.0039 | *0.0058* | 0 | 0.0108 | 0.0011 | 0 | 0 | 0 | 0.0203 | *0.0082* | *0.0064* |
| A*68~B*51 | 0.0119 | 0.0045 | *0.0133* | 0.006 | 0.0118 | 0.0196 | 0.01 | 0.0085 | 0 | 0.008 | *0.0064* | *0.0076* |
| A*68~B*53:01 | 0.0179 | 0.0015 | *0.0088* | 0.0119 | 0.0024 | 0.0138 | 0 | 0.0065 | 0.0143 | 0 | *0.0117* | *0.0059* |
| A*80:01~B*58:01/11 | 0 | 0 | *0.0133* | 0 | 0 | 0 | 0 | 0 | 0 | 0 | *0* | *0.0007* |
| blank~B*07 | 0 | 0 | *0.0061* | 0 | 0.0009 | 0.0103 | 0.0066 | 0 | 0 | 0 | *0* | *0.0011* |
| blank~B*15 | 0.0119 | 0 | *0.0127* | 0.008 | 0.0039 | 0 | 0.0032 | 0 | 0 | 0.007 | *0.0009* | *0* |
| blank~B*51 | 0.009 | 0 | *0* | 0 | 0 | 0 | 0.0053 | 0.0173 | 0 | 0 | *0* | *0* |
| blank~B*55 | 0 | 0 | *0* | 0 | 0 | 0.0012 | 0 | 0 | 0.0128 | 0 | *0* | *0.0004* |

**HLA-A-C**

| HAPLOTYPES | AA (n=65) | BE (n=570) | BS (n=80) | GE (n=61) | GR (n=105) | LG (n=77) | LS (n=128) | LU (n=86) | SG (n=79) | SI (n=76) | ZH (n=181) | All (n=1508) |
| --- | --- | --- | --- | --- | --- | --- | --- | --- | --- | --- | --- | --- |
| A*01~C*03:03/20N | 0 | 0.0037 | 0.0124 | 0 | 0 | 0 | 0.0117 | 0 | 0 | 0 | 0 | *0.0028* |
| A*01~C*04:01/09N/28/30 | 0 | 0.0045 | 0.0271 | 0 | 0 | 0 | 0 | 0.0291 | 0 | 0 | 0.0146 | *0.0067* |
| A*01~C*06:02 | 0.0231 | 0.0201 | 0.0189 | 0.0082 | 0.0246 | 0.0221 | 0.0461 | 0.0058 | 0.0506 | 0.0526 | 0.019 | *0.0239* |
| A*01~C*07:01/06/18/52 | 0.0489 | 0.0708 | 0.0597 | 0.0738 | 0.0311 | 0.0558 | 0.0714 | 0.0349 | 0.0707 | 0.071 | 0.0457 | *0.0619* |
| A*01~C*07:02/50 | 0.0098 | 0.0052 | 0.0155 | 0 | 0 | 0 | 0 | 0 | 0 | 0 | 0 | *0.004* |
| A*01~C*12:02 | 0.0168 | 0.0026 | 0 | 0.0082 | 0.0095 | 0 | 0.0021 | 0 | 0.0063 | 0 | 0.0036 | *0.0047* |
| A*01~C*12:03 | 0.0168 | 0.0013 | 0 | 0 | 0 | 0 | 0.0021 | 0.0039 | 0.0116 | 0.0038 | 0 | *0.0021* |
| A*01~C*15:02/13 | 0 | 0 | 0.0125 | 0 | 0 | 0 | 0.0014 | 0 | 0 | 0 | 0 | *0.0004* |
| A*02~blank | 0 | 0 | 0 | 0 | 0 | 0 | 0.0119 | 0.0204 | 0.0109 | 0 | 0 | *0* |
| A*02~C*01:02 | 0.0231 | 0.0202 | 0.0125 | 0 | 0.0046 | 0.0137 | 0.0134 | 0.0255 | 0.0066 | 0.0186 | 0.0115 | *0.0157* |
| A*02~C*02:02 | 0.0085 | 0.0076 | 0 | 0.0164 | 0.0034 | 0 | 0.0247 | 0.0072 | 0.0127 | 0.0091 | 0.0225 | *0.0118* |
| A*02~C*03:03/20N | 0.02 | 0.0274 | 0.0355 | 0.0142 | 0.0134 | 0 | 0 | 0.0057 | 0.0137 | 0 | 0.0395 | *0.0231* |
| A*02~C*03:04 | 0.04 | 0.0283 | 0.0139 | 0.0425 | 0.0041 | 0.005 | 0.0293 | 0 | 0.0546 | 0 | 0.0218 | *0.025* |
| A*02~C*04:01/09N/28/30 | 0.0607 | 0.0232 | 0.0114 | 0 | 0.0348 | 0.0325 | 0.0295 | 0.0255 | 0.0127 | 0.0626 | 0.0165 | *0.0227* |
| A*02~C*05:01/03 | 0.084 | 0.0381 | 0.0375 | 0.0656 | 0.0138 | 0.0448 | 0.0252 | 0.0275 | 0.0487 | 0.0263 | 0.0328 | *0.0371* |
| A*02~C*06:02 | 0.0385 | 0.0223 | 0.0615 | 0.0089 | 0.04 | 0.0195 | 0.0089 | 0.054 | 0 | 0.0263 | 0 | *0.0237* |
| A*02~C*07:01/06/18/52 | 0.0127 | 0.022 | 0.0381 | 0.0115 | 0.0103 | 0.0615 | 0.0555 | 0.0121 | 0.0313 | 0.0268 | 0.0077 | *0.0261* |
| A*02~C*07:02/50 | 0 | 0.0302 | 0.0207 | 0.0459 | 0.0506 | 0.0175 | 0.015 | 0.0195 | 0.0697 | 0.0348 | 0.0341 | *0.0282* |
| A*02~C*08:02 | 0.0077 | 0.002 | 0.0068 | 0.0164 | 0.0095 | 0 | 0.0043 | 0.0039 | 0 | 0.0132 | 0.0029 | *0.004* |
| A*02~C*12:02 | 0.0126 | 0.0018 | 0 | 0 | 0 | 0 | 0 | 0 | 0 | 0 | 0.0048 | *0.0023* |
| A*02~C*12:03 | 0 | 0.0135 | 0.0094 | 0 | 0 | 0.0325 | 0.0091 | 0 | 0.0305 | 0.0059 | 0.016 | *0.0121* |
| A*02~C*14:02 | 0.0385 | 0.0049 | 0 | 0 | 0.019 | 0.0508 | 0 | 0 | 0 | 0 | 0.0153 | *0.0109* |
| A*02~C*15:02/13 | 0 | 0.0225 | 0 | 0.0082 | 0.0048 | 0.0061 | 0.0053 | 0.0155 | 0.0008 | 0 | 0.0221 | *0.0144* |
| A*02~C*16:01 | 0 | 0.0018 | 0 | 0 | 0 | 0 | 0.0054 | 0 | 0.019 | 0 | 0 | *0.0028* |
| A*02~C*16:02 | 0 | 0 | 0 | 0.0121 | 0.0048 | 0 | 0.0024 | 0 | 0 | 0 | 0.0055 | *0.0027* |
| A*03~blank | 0.0092 | 0 | 0 | 0.0102 | 0 | 0.0076 | 0 | 0 | 0 | 0 | 0 | *0* |
| A*03~C*01:02 | 0.0154 | 0.0046 | 0 | 0.0164 | 0 | 0 | 0 | 0 | 0.0059 | 0 | 0.0128 | *0.0053* |
| A*03~C*03:03/20N | 0 | 0.0016 | 0.0101 | 0 | 0 | 0 | 0 | 0 | 0 | 0.015 | 0.0046 | *0.0027* |
| A*03~C*03:04 | 0.0112 | 0.006 | 0.0218 | 0.0013 | 0 | 0.0087 | 0 | 0.0115 | 0 | 0 | 0.0131 | *0.0079* |
| A*03~C*04:01/09N/28/30 | 0.0384 | 0.0304 | 0.0103 | 0.0307 | 0.0143 | 0.0187 | 0.0274 | 0.0523 | 0.0127 | 0.0244 | 0.0297 | *0.0275* |
| A*03~C*05:01/03 | 0 | 0.01 | 0 | 0 | 0 | 0 | 0.0117 | 0 | 0 | 0 | 0.0128 | *0.0083* |
| A*03~C*06:02 | 0 | 0.009 | 0 | 0.0157 | 0 | 0 | 0 | 0 | 0.0063 | 0 | 0.0137 | *0.0059* |
| HAPLOTYPES | AA (n=65) | BE (n=570) | BS (n=80) | GE (n=61) | GR (n=105) | LG (n=77) | LS (n=128) | LU (n=86) | SG (n=79) | SI (n=76) | ZH (n=181) | All (n=1508) |
| A*03~C*07:01/06/18/52 | 0 | 0.0032 | 0.0169 | 0.0032 | 0.0057 | 0.009 | 0.0047 | 0.0185 | 0.0063 | 0 | 0.0136 | *0.0047* |
| A*03~C*07:02/50 | 0.0258 | 0.0614 | 0.0578 | 0 | 0.0571 | 0.0296 | 0.0768 | 0.072 | 0.0316 | 0.0329 | 0.0274 | *0.0527* |
| A*03~C*15:02/13 | 0.0077 | 0.0036 | 0 | 0 | 0 | 0.013 | 0 | 0 | 0 | 0.0066 | 0.011 | *0.0041* |
| A*03~C*16:01 | 0 | 0.0016 | 0 | 0 | 0.0048 | 0 | 0.0122 | 0 | 0.0016 | 0.0033 | 0 | *0.0016* |
| A*11~C*01:02 | 0 | 0.0023 | 0 | 0 | 0 | 0 | 0.01 | 0 | 0 | 0 | 0.0097 | *0.003* |
| A*11~C*03:03/20N | 0.017 | 0.0064 | 0.0125 | 0 | 0.027 | 0 | 0.0117 | 0.0058 | 0.0095 | 0.0179 | 0.0033 | *0.0087* |
| A*11~C*04:01/09N/28/30 | 0 | 0.0248 | 0.0352 | 0.0246 | 0.0349 | 0.039 | 0.0174 | 0.0116 | 0.0095 | 0.0132 | 0.0131 | *0.0222* |
| A*11~C*05:01/03 | 0 | 0.0049 | 0 | 0 | 0 | 0 | 0.0103 | 0 | 0.0127 | 0.0132 | 0 | *0.004* |
| A*11~C*07:01/06/18/52 | 0.0138 | 0.0058 | 0 | 0.0016 | 0 | 0 | 0 | 0 | 0 | 0 | 0 | *0.0027* |
| A*11~C*07:02/50 | 0 | 0.005 | 0.0086 | 0.0016 | 0 | 0 | 0.002 | 0.0116 | 0 | 0 | 0 | *0.0044* |
| A*11~C*08:02 | 0 | 0.0018 | 0 | 0.0164 | 0 | 0 | 0 | 0 | 0 | 0 | 0 | *0.0013* |
| A*11~C*12:02 | 0 | 0.0035 | 0 | 0 | 0 | 0.013 | 0 | 0 | 0.0127 | 0 | 0.0107 | *0.0038* |
| A*11~C*15:02/13 | 0.0231 | 0.0077 | 0 | 0 | 0 | 0.0065 | 0 | 0 | 0 | 0.015 | 0.0083 | *0.0061* |
| A*11~C*16:04 | 0 | 0 | 0 | 0 | 0 | 0 | 0 | 0.0116 | 0 | 0 | 0 | *0* |
| A*23~C*04:01/09N/28/30 | 0.0077 | 0.0102 | 0.0063 | 0 | 0 | 0.0022 | 0.0088 | 0 | 0.0021 | 0.0329 | 0.0154 | *0.0115* |
| A*23~C*06:02 | 0 | 0 | 0 | 0.0164 | 0.0048 | 0 | 0 | 0.0116 | 0 | 0 | 0 | *0.0014* |
| A*23~C*07:01/06/18/52 | 0 | 0.0173 | 0 | 0 | 0.0032 | 0.013 | 0.0045 | 0 | 0 | 0.0132 | 0.0067 | *0.0111* |
| A*23~C*07:02/50 | 0 | 0.0009 | 0 | 0 | 0 | 0 | 0 | 0 | 0.0127 | 0 | 0 | *0.0008* |
| A*23~C*16:01 | 0 | 0 | 0 | 0 | 0 | 0 | 0.0112 | 0.0015 | 0 | 0 | 0 | *0.0012* |
| A*24~C*01:02 | 0 | 0.0033 | 0.0063 | 0 | 0.0238 | 0 | 0 | 0 | 0 | 0 | 0 | *0.0034* |
| A*24~C*02:02 | 0.0222 | 0.0046 | 0.0187 | 0 | 0 | 0 | 0.0039 | 0 | 0 | 0.0059 | 0.0123 | *0.0061* |
| A*24~C*03:03/20N | 0.0127 | 0.0093 | 0 | 0.0164 | 0 | 0 | 0.0146 | 0.0058 | 0.0098 | 0.0179 | 0.0182 | *0.0101* |
| A*24~C*04:01/09N/28/30 | 0.0197 | 0.0178 | 0.0349 | 0 | 0 | 0.0173 | 0.0169 | 0.0174 | 0.0506 | 0.0116 | 0.0173 | *0.0177* |
| A*24~C*05:01/03 | 0 | 0.0015 | 0 | 0 | 0.0286 | 0 | 0 | 0.0058 | 0 | 0 | 0.0075 | *0.0022* |
| A*24~C*06:02 | 0 | 0.0073 | 0 | 0 | 0.0143 | 0.0299 | 0 | 0.0143 | 0.0316 | 0 | 0.0151 | *0.0091* |
| A*24~C*07:01/06/18/52 | 0 | 0.0068 | 0.0199 | 0 | 0.0167 | 0.0506 | 0.0078 | 0.0172 | 0 | 0.0054 | 0 | *0.0102* |
| A*24~C*07:02/50 | 0.0231 | 0.0236 | 0.0185 | 0.0328 | 0 | 0 | 0.0183 | 0.015 | 0 | 0.0126 | 0.0238 | *0.0203* |
| A*24~C*07:24 | 0 | 0 | 0 | 0 | 0.0167 | 0.0126 | 0.0078 | 0 | 0 | 0 | 0 | *0.0006* |
| A*24~C*08:02 | 0 | 0.0029 | 0.008 | 0.0164 | 0.0095 | 0 | 0 | 0 | 0 | 0.0197 | 0 | *0.003* |
| A*24~C*12:02 | 0 | 0 | 0.0063 | 0 | 0.0016 | 0 | 0.0156 | 0 | 0.0011 | 0.0022 | 0 | *0.0016* |
| A*24~C*12:03 | 0.0154 | 0.0059 | 0 | 0.0164 | 0.0016 | 0 | 0 | 0 | 0.0011 | 0.0022 | 0.0077 | *0.0063* |
| A*24~C*15:02/13 | 0 | 0.009 | 0 | 0.0041 | 0 | 0.0065 | 0.0039 | 0 | 0.019 | 0 | 0 | *0.0045* |
| A*25:01~C*12:03 | 0 | 0.0106 | 0.0187 | 0 | 0 | 0.0065 | 0 | 0.0174 | 0 | 0 | 0 | *0.0079* |
| A*26~C*01:02 | 0 | 0.0018 | 0 | 0.0082 | 0 | 0.0188 | 0 | 0 | 0.0124 | 0.0066 | 0 | *0.0023* |
| HAPLOTYPES | AA (n=65) | BE (n=570) | BS (n=80) | GE (n=61) | GR (n=105) | LG (n=77) | LS (n=128) | LU (n=86) | SG (n=79) | SI (n=76) | ZH (n=181) | All (n=1508) |
| A*26~C*02:02 | 0 | 0.0059 | 0 | 0 | 0 | 0 | 0 | 0.0058 | 0.0127 | 0 | 0.0028 | *0.0036* |
| A*26~C*04:01/09N/28/30 | 0.0038 | 0.0019 | 0 | 0 | 0.019 | 0.013 | 0 | 0 | 0 | 0 | 0 | *0.0002* |
| A*26~C*06:02 | 0.0077 | 0.0036 | 0 | 0 | 0.0127 | 0 | 0.0052 | 0 | 0 | 0.0197 | 0 | *0.004* |
| A*26~C*07:01/06/18/52 | 0 | 0.0053 | 0 | 0.0013 | 0 | 0 | 0 | 0.0168 | 0.0297 | 0 | 0 | *0.0056* |
| A*26~C*12:03 | 0 | 0.0107 | 0.0167 | 0.0246 | 0.0048 | 0 | 0.0104 | 0.0291 | 0.0022 | 0 | 0.0014 | *0.0104* |
| A*29~C*16:01 | 0.0231 | 0.0166 | 0.0125 | 0.0328 | 0.0238 | 0.0325 | 0.0273 | 0.0407 | 0.0063 | 0.0197 | 0.0161 | *0.0208* |
| A*30:01/24~C*02:02 | 0 | 0.0009 | 0 | 0 | 0 | 0 | 0 | 0.016 | 0 | 0 | 0 | *0* |
| A*30:01/24~C*04:01/09N/28/30 | 0.0104 | 0 | 0 | 0.002 | 0 | 0 | 0 | 0 | 0 | 0 | 0 | *0.0021* |
| A*30:01/24~C*06:02 | 0.0038 | 0.0131 | 0.0071 | 0.0328 | 0.006 | 0 | 0.0039 | 0.0166 | 0.0169 | 0.0132 | 0.0122 | *0.0167* |
| A*30:01/24~C*07:02/50 | 0.0126 | 0 | 0 | 0.001 | 0 | 0 | 0 | 0 | 0 | 0 | 0 | *0.0007* |
| A*30:02~C*05:01/03 | 0 | 0.0079 | 0 | 0.041 | 0 | 0 | 0 | 0.0031 | 0 | 0.0022 | 0.0083 | *0.0069* |
| A*30:15~C*06:02 | 0 | 0 | 0 | 0 | 0.006 | 0 | 0.0039 | 0 | 0 | 0 | 0.0122 | *0* |
| A*31~C*03:04 | 0 | 0.0079 | 0.0187 | 0.0013 | 0.0019 | 0 | 0.001 | 0 | 0 | 0.0049 | 0.0157 | *0.0084* |
| A*31~C*04:01/09N/28/30 | 0 | 0.0022 | 0.0063 | 0.0164 | 0 | 0 | 0 | 0 | 0 | 0 | 0.0009 | *0.0024* |
| A*31~C*05:01/03 | 0.0083 | 0.0016 | 0 | 0 | 0.0048 | 0 | 0.003 | 0 | 0.0042 | 0.0197 | 0.0066 | *0.0039* |
| A*31~C*07:01/06/18/52 | 0.0148 | 0.0015 | 0 | 0 | 0.0016 | 0.0065 | 0 | 0 | 0 | 0 | 0.0075 | *0.0032* |
| A*31~C*12:03 | 0 | 0.0017 | 0 | 0 | 0 | 0 | 0.0156 | 0 | 0 | 0.0008 | 0 | *0.0025* |
| A*32:01~blank | 0 | 0 | 0.0007 | 0.001 | 0.0124 | 0.013 | 0 | 0 | 0 | 0 | 0 | *0* |
| A*32:01~C*01:02 | 0 | 0 | 0 | 0 | 0.0143 | 0 | 0 | 0.0116 | 0 | 0.0077 | 0.0102 | *0.0029* |
| A*32:01~C*02:02 | 0 | 0.0085 | 0 | 0.0072 | 0.0084 | 0.0195 | 0.0117 | 0 | 0.0127 | 0.0186 | 0.0014 | *0.0093* |
| A*32:01~C*03:03/20N | 0.0154 | 0.0067 | 0 | 0.0082 | 0 | 0 | 0 | 0.0087 | 0.0127 | 0 | 0 | *0.0045* |
| A*32:01~C*05:01/03 | 0 | 0.0083 | 0.0187 | 0 | 0 | 0 | 0.0039 | 0 | 0 | 0 | 0.0034 | *0.0069* |
| A*32:01~C*06:02 | 0.0154 | 0 | 0 | 0 | 0.0068 | 0 | 0.0156 | 0.0116 | 0 | 0 | 0 | *0.0025* |
| A*32:01~C*07:01/06/18/52 | 0.0026 | 0.0047 | 0 | 0.0164 | 0.0014 | 0 | 0 | 0 | 0.0005 | 0 | 0 | *0.0031* |
| A*32:01~C*08:02 | 0 | 0 | 0 | 0.0082 | 0.0143 | 0 | 0.0039 | 0 | 0 | 0.0066 | 0 | *0.0018* |
| A*33~C*04:01/09N/28/30 | 0 | 0 | 0.0125 | 0 | 0 | 0 | 0 | 0 | 0 | 0 | 0 | *0.0004* |
| A*33~C*08:02 | 0.0077 | 0.0035 | 0.0114 | 0 | 0 | 0.0325 | 0 | 0.0058 | 0.0013 | 0 | 0.0138 | *0.0071* |
| A*33~C*17 | 0 | 0 | 0 | 0.0164 | 0.0048 | 0 | 0 | 0 | 0 | 0 | 0 | *0.0008* |
| A*68~blank | 0 | 0 | 0.0125 | 0 | 0 | 0 | 0 | 0 | 0 | 0 | 0.0053 | *0* |
| A*68~C*03:04 | 0 | 0.0061 | 0 | 0 | 0 | 0 | 0 | 0.0058 | 0.019 | 0 | 0.0054 | *0.0047* |
| A*68~C*04:01/09N/28/30 | 0 | 0.0071 | 0 | 0 | 0.0113 | 0.0074 | 0 | 0 | 0 | 0 | 0.0204 | *0.0097* |
| A*68~C*05:01/03 | 0 | 0 | 0 | 0.0082 | 0.0078 | 0 | 0.0013 | 0.0116 | 0 | 0 | 0.0032 | *0.0027* |
| A*68~C*07:01/06/18/52 | 0.0115 | 0.001 | 0 | 0 | 0.0016 | 0.0155 | 0.0021 | 0 | 0 | 0 | 0 | *0.0026* |
| A*68~C*07:02/50 | 0.0115 | 0.0045 | 0 | 0 | 0 | 0 | 0.002 | 0 | 0 | 0 | 0.0186 | *0.004* |
| HAPLOTYPES | AA (n=65) | BE (n=570) | BS (n=80) | GE (n=61) | GR (n=105) | LG (n=77) | LS (n=128) | LU (n=86) | SG (n=79) | SI (n=76) | ZH (n=181) | All (n=1508) |
| A*68~C*07:04/11 | 0 | 0.005 | 0.0067 | 0 | 0.0048 | 0 | 0 | 0.0233 | 0 | 0.0197 | 0 | *0.0052* |
| A*68~C*15:02/13 | 0.001 | 0.0029 | 0.0121 | 0.0082 | 0 | 0 | 0.0012 | 0 | 0 | 0.0179 | 0 | *0.0033* |
| blank~C*03:03/20N | 0 | 0 | 0.0125 | 0 | 0.0117 | 0 | 0 | 0.0001 | 0.0095 | 0 | 0 | *0* |
| blank~C*03:04 | 0 | 0 | 0 | 0 | 0 | 0.0166 | 0 | 0 | 0 | 0.0103 | 0 | *0.0007* |
| blank~C*05:01/03 | 0 | 0 | 0 | 0 | 0.0117 | 0 | 0 | 0 | 0 | 0 | 0 | *0.0007* |
| blank~C*07:01/06/18/52 | 0 | 0 | 0 | 0 | 0 | 0 | 0.0104 | 0 | 0 | 0 | 0.0049 | *0* |

**HLA-B-C**

| HAPLOTYPES | AA (n=75) | BE (n=594) | BS (n=119) | GE (n=83) | GR (n=122) | LG (n=95) | LS (n=129) | LU (n=120) | SG (n=90) | SI (n=77) | ZH (n=245) | All (n=1749) |
| --- | --- | --- | --- | --- | --- | --- | --- | --- | --- | --- | --- | --- |
| B*07~C*07:02/50 | 0.1032 | 0.1253 | *0.092* | 0.0644 | 0.127 | 0.042 | 0.1047 | 0.1086 | 0.111 | 0.0714 | *0.079* | *0.1031* |
| B*07~C*15:05 | 0 | 0.0025 | *0.004* | 0.012 | 0.0014 | 0 | 0.0078 | 0.0056 | 0 | 0.0022 | *0.006* | *0.0051* |
| B*08~C*07:01/06/18/52 | 0.0436 | 0.0816 | *0.05* | 0.0843 | 0.0488 | 0.0684 | 0.1085 | 0.0417 | 0.0722 | 0.0767 | *0.053* | *0.0698* |
| B*13:02~C*06:02 | 0.0267 | 0.0303 | *0.013* | 0.0009 | 0.0328 | 0.0211 | 0.0194 | 0.0333 | 0.0389 | 0.026 | *0.02* | *0.026* |
| B*14:01~C*08:02 | 0.0067 | 0.0059 | *0.004* | 0.012 | 0.0143 | 0.0053 | 0.0078 | 0.0008 | 0.0069 | 0.013 | *0.016* | *0.0083* |
| B*14:02~C*08:02 | 0.0133 | 0.0152 | *0.038* | 0.0422 | 0.0143 | 0.0316 | 0.0078 | 0.0083 | 0.0208 | 0.0325 | *0.016* | *0.0191* |
| B*15~C*01:02 | 0.0051 | 0.0059 | *0* | 0 | 0 | 0.0105 | 0.0044 | 0.0083 | 0 | 0 | *0.006* | *0.0047* |
| B*15~C*03:03/20N | 0.0444 | 0.0435 | *0.052* | 0.0289 | 0.0318 | 0.0237 | 0.0383 | 0.01 | 0.0296 | 0.0324 | *0.033* | *0.0371* |
| B*15~C*03:04 | 0.0067 | 0.0096 | *0.003* | 0.0193 | 0.0036 | 0.002 | 0 | 0.015 | 0.0198 | 0.0065 | *0.024* | *0.0128* |
| B*15~C*04:01/09N/28/30 | 0.0267 | 0.0024 | *0* | 0 | 0.0082 | 0 | 0 | 0.0042 | 0 | 0.0066 | *0.006* | *0.0045* |
| B*15~C*07:01/06/18/52 | 0 | 0.0023 | *0.021* | 0 | 0 | 0.005 | 0.0101 | 0 | 0.0056 | 0.0043 | *0.001* | *0.0053* |
| B*15~C*07:04/11 | 0 | 0.0033 | *0.008* | 0 | 0.0042 | 0.0079 | 0 | 0.0208 | 0 | 0 | *0.002* | *0.0044* |
| B*15~C*12:03 | 0 | 0.0017 | *0* | 0.0118 | 0 | 0.0105 | 0.0015 | 0 | 0.009 | 0.0022 | *0.001* | *0.0031* |
| B*15~C*12:13 | 0 | 0 | *0* | 0.0118 | 0 | 0 | 0.0015 | 0 | 0.0004 | 0.0022 | *0.003* | *0* |
| B*18~C*01:02 | 0 | 0 | *0* | 0.012 | 0 | 0 | 0 | 0 | 0 | 0 | *0* | *0.0003* |
| B*18~C*05:01/03 | 0.0047 | 0.0135 | *0* | 0.0301 | 0 | 0.0112 | 0.0077 | 0.0167 | 0.006 | 0.013 | *0.008* | *0.0106* |
| B*18~C*07:01/06/18/52 | 0.0267 | 0.0252 | *0.029* | 0.012 | 0.0287 | 0.0467 | 0.0108 | 0.025 | 0.0218 | 0.0065 | *0.011* | *0.0226* |
| B*18~C*12:03 | 0.0011 | 0.0168 | *0.021* | 0.006 | 0.0082 | 0.0158 | 0.0078 | 0.0125 | 0.0056 | 0 | *0.005* | *0.013* |
| B*27:02~C*02:02 | 0 | 0.0062 | *0.003* | 0.0015 | 0.001 | 0.0053 | 0 | 0 | 0.0074 | 0.013 | *0.003* | *0.0053* |
| B*27:05/13~C*01:02 | 0.0067 | 0.0133 | *0.008* | 0.002 | 0.0131 | 0.0053 | 0.0233 | 0 | 0.0056 | 0.013 | *0.011* | *0.0108* |
| B*27:05/13~C*02:02 | 0 | 0.0132 | *0.013* | 0 | 0.0026 | 0.0053 | 0.0155 | 0.0208 | 0.0148 | 0.0104 | *0.008* | *0.0118* |
| B*35:01/40N/42/57/94~C*04:01/09N/28/30 | 0.04 | 0.0732 | *0.084* | 0.0426 | 0.0343 | 0.0858 | 0.0575 | 0.0494 | 0.0202 | 0.0259 | *0.051* | *0.0599* |
| B*35:01/40N/42/57/94~C*07:01/06/18/52 | 0 | 0 | *0.004* | 0 | 0 | 0.0103 | 0 | 0 | 0 | 0 | *0* | *0.0011* |
| B*35:02~C*04:01/09N/28/30 | 0 | 0.0071 | *0.025* | 0.017 | 0 | 0.0079 | 0.0048 | 0.0279 | 0.0202 | 0.0173 | *0.01* | *0.0106* |
| B*35:03/70~C*04:01/09N/28/30 | 0.0133 | 0.0197 | *0.028* | 0.017 | 0.0514 | 0 | 0.0192 | 0.0174 | 0.0472 | 0.0259 | *0.044* | *0.0257* |
| B*35:03/70~C*12:03 | 0 | 0.0042 | *0* | 0.006 | 0 | 0.0158 | 0.0013 | 0 | 0.0056 | 0.0032 | *0.004* | *0.005* |
| B*35:36~C*04:01/09N/28/30 | 0 | 0 | *0* | 0 | 0 | 0 | 0 | 0.0174 | 0 | 0 | *0* | *0* |
| B*37:01~C*06:02 | 0.0153 | 0.0093 | *0.013* | 0.003 | 0.0184 | 0.0037 | 0.0116 | 0.0125 | 0.0167 | 0.0097 | *0.01* | *0.0127* |
| B*37:03N~C*06:02 | 0 | 0 | *0* | 0.003 | 0.0184 | 0 | 0 | 0 | 0 | 0.0097 | *0* | *0* |
| B*38:01~C*12:03 | 0.0267 | 0.0152 | *0.027* | 0.0422 | 0.0205 | 0.0158 | 0.0194 | 0.0031 | 0.0222 | 0.0043 | *0.02* | *0.0199* |
| B*39:01~C*07:02/50 | 0 | 0.0004 | *0* | 0 | 0 | 0 | 0 | 0.0003 | 0.0111 | 0 | *4E-04* | *0.0015* |
| B*39:01~C*12:03 | 0.0133 | 0.0084 | *0.013* | 0.0001 | 0.0003 | 0.0211 | 0.0271 | 0.0004 | 0.0056 | 0.0003 | *0.006* | *0.01* |
| HAPLOTYPES | AA (n=75) | BE (n=594) | BS (n=119) | GE (n=83) | GR (n=122) | LG (n=95) | LS (n=129) | LU (n=120) | SG (n=90) | SI (n=77) | ZH (n=245) | All (n=1749) |
| B*39:06~C*07:02/50 | 0.0133 | 0.0034 | *0.004* | 0.012 | 0.002 | 0.0018 | 0.0013 | 0.0083 | 0.0011 | 0.0065 | *0.008* | *0.0059* |
| B*40:01/55~C*03:04 | 0.0267 | 0.0412 | *0.046* | 0.0314 | 0.0039 | 0.0053 | 0.0305 | 0.025 | 0.0389 | 0.0081 | *0.042* | *0.0358* |
| B*40:02/56~C*02:02 | 0.0067 | 0.0109 | *0.002* | 0 | 0.0061 | 0.0039 | 0.0155 | 0.0083 | 0.0111 | 0.013 | *0.012* | *0.012* |
| B*41:01~C*07:01/06/18/52 | 0.0067 | 0.0008 | *0* | 0 | 0 | 0 | 0 | 0 | 0 | 0 | *0.01* | *0.0017* |
| B*41:01~C*17 | 0.0067 | 0.0008 | *0.002* | 0.012 | 0 | 0 | 0 | 0 | 0 | 0 | *0* | *0.002* |
| B*41:02~C*17 | 0 | 0.0051 | *0* | 0.012 | 0.0041 | 0 | 0 | 0.0042 | 0.0056 | 0.0065 | *0.002* | *0.0037* |
| B*44:02/19N/27~C*05:01/03 | 0.0533 | 0.0639 | *0.05* | 0.0458 | 0.0738 | 0.0263 | 0.0543 | 0.0417 | 0.0607 | 0.0584 | *0.063* | *0.0576* |
| B*44:02/19N/27~C*07:04/11 | 0.0133 | 0.0135 | *0.008* | 0.0012 | 0.0122 | 0 | 0.0078 | 0 | 0.0171 | 0.0195 | *0.004* | *0.0099* |
| B*44:03~C*04:01/09N/28/30 | 0.04 | 0.0167 | *0.013* | 0.012 | 0.0123 | 0.0004 | 0.0233 | 0.0208 | 0.0111 | 0.039 | *0.022* | *0.0189* |
| B*44:03~C*07:01/06/18/52 | 0 | 0.0035 | *0* | 0 | 0 | 0.0158 | 0.0022 | 0 | 0 | 0 | *0* | *0.0018* |
| B*44:03~C*16:01 | 0.0267 | 0.0278 | *0.021* | 0.0301 | 0.0287 | 0.0263 | 0.0504 | 0.05 | 0.0278 | 0.026 | *0.018* | *0.0292* |
| B*44:05~C*02:02 | 0.02 | 0.0059 | *0* | 0 | 0.0015 | 0.0004 | 0 | 0 | 0 | 0.0016 | *0.002* | *0.0047* |
| B*45~C*06:02 | 0 | 0.0008 | *0.004* | 0.006 | 0 | 0 | 0.0039 | 0 | 0 | 0.0195 | *0* | *0.002* |
| B*49:01~C*07:01/06/18/52 | 0.0033 | 0.021 | *0.004* | 0.0169 | 0.0061 | 0.0211 | 0.0155 | 0.0375 | 0.0056 | 0.013 | *0.012* | *0.0176* |
| B*49:01~C*07:24 | 0.0033 | 0 | *0* | 0.0169 | 0.0061 | 0 | 0 | 0 | 0 | 0 | *0* | *0* |
| B*50:01~C*06:02 | 0.02 | 0.0084 | *0.008* | 0.0181 | 0.0246 | 0.0211 | 0.0116 | 0.0194 | 0.0167 | 0 | *0.018* | *0.0136* |
| B*51~C*01:02 | 0.0133 | 0.0093 | *0.004* | 0.0181 | 0.032 | 0.0053 | 0.0034 | 0.0116 | 0.0167 | 0.0195 | *0.024* | *0.0134* |
| B*51~C*02:02 | 0 | 0.0035 | *0.008* | 0.0175 | 0.009 | 0 | 0.0116 | 0.0058 | 0 | 0.0135 | *0.005* | *0.0063* |
| B*51~C*04:01/09N/28/30 | 0 | 0.0043 | *0* | 0 | 0 | 0.0168 | 0.0039 | 0 | 0 | 0 | *0* | *0.0025* |
| B*51~C*05:01/03 | 0 | 0.0032 | *0.004* | 0 | 0 | 0.0151 | 0.0078 | 0 | 0.0037 | 0 | *0.001* | *0.0036* |
| B*51~C*14:02 | 0.0333 | 0.0109 | *0.001* | 0.0012 | 0.0246 | 0.0632 | 0.0155 | 0.0167 | 0.0056 | 0.0022 | *0.02* | *0.0169* |
| B*51~C*15:02/13 | 0.0533 | 0.0483 | *0.028* | 0.0241 | 0.0055 | 0.0474 | 0.0426 | 0.0333 | 0.0277 | 0.0519 | *0.048* | *0.0407* |
| B*51~C*16:02 | 0 | 0.0042 | *0.004* | 0.006 | 0 | 0.0005 | 0.0116 | 0 | 0.0111 | 0.013 | *0.004* | *0.0049* |
| B*52:01/07~C*12:02 | 0.0267 | 0.0084 | *0.013* | 0.0181 | 0.0082 | 0.0053 | 0.0078 | 0 | 0.0222 | 0 | *0.016* | *0.0108* |
| B*53:01~C*04:01/09N/28/30 | 0.0067 | 0.0034 | *0.004* | 0 | 0.0061 | 0.0009 | 0.0019 | 0.0021 | 0.0056 | 0.0011 | *0.011* | *0.0059* |
| B*55~C*01:02 | 0 | 0 | *0* | 0.012 | 0 | 0.0053 | 0 | 0 | 0.0056 | 0 | *0* | *0.0011* |
| B*55~C*03:03/20N | 0.0133 | 0.0134 | *0.013* | 0.0241 | 0.0041 | 0.0105 | 0.0155 | 0.0167 | 0.0278 | 0.0195 | *0.022* | *0.0159* |
| B*56~C*01:02 | 0.0267 | 0.0067 | *0.004* | 0 | 0.002 | 0.0105 | 0 | 0.0083 | 0.0056 | 0.0065 | *0.005* | *0.0069* |
| B*57:01~C*06:02 | 0.0662 | 0.0261 | *0.046* | 0.0361 | 0.0246 | 0.0526 | 0.0388 | 0.0455 | 0.0594 | 0.0325 | *0.047* | *0.0385* |
| B*58:01/11~C*03:02 | 0 | 0.0076 | *0.017* | 0.002 | 0 | 0.0035 | 0.001 | 0.0125 | 0.0111 | 0 | *0.006* | *0.0071* |
| B*58:01/11~C*07:01/06/18/52 | 0 | 0.0025 | *0.006* | 0.012 | 0.0041 | 0.0105 | 0.0116 | 0.0021 | 0 | 0 | *0.012* | *0.0063* |
| blank~blank | 0.0184 | 0 | *0.004* | 0.0149 | 0 | 0 | 0 | 0.0017 | 0 | 0 | *0.009* | *0.0015* |

**HLA-A-DRB1**

| HAPLOTYPES | AA (n=103) | BE (n=709) | BS (n=126) | GE (n=105) | GR (n=228) | LG (n=124) | LS (n=364) | LU (n=151) | SG (n=111) | SI (n=153) | ZH (n=291) | All (n=2465) |
| --- | --- | --- | --- | --- | --- | --- | --- | --- | --- | --- | --- | --- |
| A*01~DRB1*01:01 | 0.0188 | 0.0081 | 0.0119 | 0 | 0.0082 | 0 | 0.0057 | 0.0038 | 0 | 0.0127 | *0* | *0.0051* |
| A*01~DRB1*03 | 0.0388 | 0.0483 | 0.0238 | 0.0365 | 0.0258 | 0.0413 | 0.047 | 0.0209 | 0.0387 | 0.0312 | *0.0328* | *0.0372* |
| A*01~DRB1*04 | 0 | 0.0105 | 0 | 0.0128 | 0.0102 | 0 | 0.0139 | 0.0143 | 0.0048 | 0.0202 | *0.012* | *0.0089* |
| A*01~DRB1*07 | 0.0237 | 0.0119 | 0.0127 | 0.011 | 0.0088 | 0.0247 | 0.0239 | 0.0087 | 0.0235 | 0 | *0.0153* | *0.0146* |
| A*01~DRB1*11 | 0 | 0.01 | 0.0327 | 0.0181 | 0 | 0.0051 | 0.0092 | 0 | 0.0184 | 0.0066 | *0.0128* | *0.0098* |
| A*01~DRB1*13:01 | 0 | 0.0018 | 0 | 0 | 0.013 | 0 | 0.0125 | 0 | 0.015 | 0 | *0* | *0.0036* |
| A*01~DRB1*13:02 | 0.0049 | 0.0057 | 0.0143 | 0 | 0 | 0 | 0 | 0.0096 | 0.0082 | 0.0098 | *0.0033* | *0.0054* |
| A*01~DRB1*14 | 0.0091 | 0.0008 | 0 | 0.0143 | 0.0022 | 0.0056 | 0 | 0 | 0.0058 | 0 | *0* | *0.003* |
| A*01~DRB1*15:01 | 0.0084 | 0.0157 | 0.0135 | 0 | 0.0151 | 0 | 0.0083 | 0.0194 | 0 | 0.0159 | *0.0105* | *0.0122* |
| A*02~blank | 0.0001 | 0 | 0 | 0 | 0 | 0 | 0 | 0 | 0.0159 | 0 | *0.0029* | *0* |
| A*02~DRB1*01:01 | 0.012 | 0.0174 | 0.0164 | 0.0098 | 0.0283 | 0.0194 | 0.0158 | 0 | 0.0447 | 0.0151 | *0.0192* | *0.0182* |
| A*02~DRB1*01:02 | 0 | 0.0013 | 0 | 0 | 0 | 0 | 0 | 0.0147 | 0 | 0 | *0* | *0.0016* |
| A*02~DRB1*03 | 0.0048 | 0.0129 | 0.0103 | 0 | 0.0003 | 0.0156 | 0.0209 | 0.0042 | 0.0306 | 0.0048 | *0* | *0.0127* |
| A*02~DRB1*04 | 0.0466 | 0.0489 | 0.0553 | 0.023 | 0.0637 | 0.0368 | 0.0364 | 0.0216 | 0.0569 | 0.0567 | *0.0546* | *0.0453* |
| A*02~DRB1*07 | 0.0386 | 0.0331 | 0.0552 | 0.0433 | 0.0464 | 0.0356 | 0.0303 | 0.0336 | 0.0198 | 0.0165 | *0.0378* | *0.0357* |
| A*02~DRB1*08 | 0.0429 | 0.0184 | 0.0088 | 0.0143 | 0.0292 | 0.0385 | 0.0099 | 0.0232 | 0.0275 | 0.0182 | *0.0191* | *0.0219* |
| A*02~DRB1*09:01 | 0.0056 | 0.0036 | 0.0119 | 0 | 0.0044 | 0 | 0.0064 | 0.0031 | 0 | 0 | *0* | *0.0033* |
| A*02~DRB1*11 | 0.0566 | 0.0352 | 0.0336 | 0.0401 | 0.0499 | 0.0469 | 0.0487 | 0.0356 | 0.0194 | 0.0521 | *0.0272* | *0.0389* |
| A*02~DRB1*13:01 | 0.023 | 0.0304 | 0.0397 | 0 | 0.0169 | 0.024 | 0.0217 | 0.0158 | 0.0253 | 0.0359 | *0.0399* | *0.0253* |
| A*02~DRB1*13:02 | 0.0327 | 0.0151 | 0.005 | 0.0312 | 0.0174 | 0 | 0.0228 | 0.0289 | 0.0161 | 0.014 | *0.0048* | *0.0176* |
| A*02~DRB1*13:03 | 0.0145 | 0.0017 | 0 | 0 | 0 | 0 | 0 | 0 | 0 | 0 | *0.002* | *0.0009* |
| A*02~DRB1*14 | 0.0101 | 0.0088 | 0.0238 | 0.0035 | 0.0047 | 0.0348 | 0.0077 | 0.0042 | 0 | 0.0049 | *0.0201* | *0.0103* |
| A*02~DRB1*15:01 | 0.0463 | 0.03 | 0.0247 | 0.0339 | 0.0188 | 0.0256 | 0.0227 | 0.0546 | 0.0425 | 0.007 | *0.0294* | *0.0274* |
| A*02~DRB1*16 | 0 | 0.0075 | 0.0058 | 0.0286 | 0.0023 | 0 | 0.0046 | 0.0159 | 0.009 | 0.0075 | *0.0023* | *0.0089* |
| A*03~blank | 0 | 0 | 0.018 | 0.0084 | 0 | 0 | 0.0072 | 0.0046 | 0.0116 | 0 | *0* | *0* |
| A*03~DRB1*01:01 | 0.0058 | 0.019 | 0.0338 | 0.0156 | 0.006 | 0.0153 | 0.0112 | 0.0165 | 0 | 0 | *0.0221* | *0.0162* |
| A*03~DRB1*03 | 0 | 0.0088 | 0 | 0 | 0.0143 | 0.0151 | 0.0058 | 0.0033 | 0 | 0.015 | *0.0157* | *0.0085* |
| A*03~DRB1*04 | 0.0049 | 0.0157 | 0.0173 | 0.0104 | 0.0171 | 0.0045 | 0.0227 | 0.0222 | 0 | 0.0088 | *0.0062* | *0.0136* |
| A*03~DRB1*07 | 0 | 0.0071 | 0.014 | 0.0041 | 0.0037 | 0.0119 | 0.0075 | 0.0051 | 0 | 0.0279 | *0* | *0.0066* |
| A*03~DRB1*08 | 0 | 0 | 0 | 0 | 0.0088 | 0.0081 | 0 | 0.018 | 0.0118 | 0.0034 | *0* | *0.0038* |
| A*03~DRB1*11 | 0.0202 | 0.0234 | 0.0077 | 0.0332 | 0.0108 | 0.0295 | 0.0151 | 0.0367 | 0.0255 | 0 | *0.0219* | *0.0199* |
| A*03~DRB1*13:01 | 0.0276 | 0.0147 | 0.0125 | 0.014 | 0.002 | 0.0056 | 0.0066 | 0 | 0 | 0.0132 | *0.0123* | *0.0117* |
| HAPLOTYPES | AA (n=103) | BE (n=709) | BS (n=126) | GE (n=105) | GR (n=228) | LG (n=124) | LS (n=364) | LU (n=151) | SG (n=111) | SI (n=153) | ZH (n=291) | All (n=2465) |
| A*03~DRB1*13:02 | 0 | 0.0028 | 0.0097 | 0 | 0.0032 | 0 | 0 | 0.007 | 0.0135 | 0 | *0.0116* | *0.0051* |
| A*03~DRB1*14 | 0.0179 | 0.0049 | 0 | 0 | 0 | 0 | 0.0056 | 0 | 0.0109 | 0.0106 | *0.0084* | *0.0062* |
| A*03~DRB1*15:01 | 0.023 | 0.0383 | 0 | 0 | 0.0458 | 0.0309 | 0.0311 | 0.0078 | 0.0097 | 0.0226 | *0.0268* | *0.0278* |
| A*11~DRB1*01:01 | 0.0173 | 0.0094 | 0.0238 | 0 | 0.0091 | 0.0089 | 0.0034 | 0.0066 | 0 | 0.006 | *0.0125* | *0.0093* |
| A*11~DRB1*03 | 0 | 0.0024 | 0 | 0.0143 | 0.0068 | 0.004 | 0.0073 | 0.0066 | 0 | 0 | *0.0057* | *0.0043* |
| A*11~DRB1*04 | 0.0272 | 0.0069 | 0 | 0.0143 | 0.0083 | 0.0113 | 0.0094 | 0.0099 | 0.0208 | 0.0078 | *0.0109* | *0.009* |
| A*11~DRB1*07 | 0 | 0.0068 | 0 | 0 | 0 | 0 | 0.0033 | 0.0132 | 0.0107 | 0.014 | *0.0038* | *0.0048* |
| A*11~DRB1*11 | 0.0059 | 0.0045 | 0 | 0 | 0.0089 | 0.0081 | 0 | 0 | 0.0045 | 0.011 | *0.0054* | *0.0052* |
| A*11~DRB1*14 | 0.0063 | 0.0099 | 0.0119 | 0.0048 | 0.0022 | 0 | 0.0168 | 0.0099 | 0 | 0.0087 | *0.0002* | *0.0081* |
| A*11~DRB1*15:01 | 0.0052 | 0.0078 | 0.0119 | 0.0095 | 0.0088 | 0 | 0.0045 | 0 | 0 | 0 | *0* | *0.0064* |
| A*11~DRB1*16 | 0 | 0 | 0 | 0 | 0.0044 | 0.0121 | 0.0022 | 0 | 0 | 0.0033 | *0.0052* | *0.0015* |
| A*23~DRB1*03 | 0 | 0.0023 | 0.004 | 0 | 0 | 0.0053 | 0.0037 | 0 | 0 | 0.015 | *0.0024* | *0.003* |
| A*23~DRB1*07 | 0.0194 | 0.0093 | 0 | 0.0067 | 0.005 | 0.0068 | 0.0112 | 0.015 | 0.0045 | 0.016 | *0.0129* | *0.0107* |
| A*23~DRB1*11 | 0 | 0.0082 | 0 | 0 | 0 | 0 | 0.0109 | 0.0066 | 0 | 0 | *0* | *0.0052* |
| A*23~DRB1*15:01 | 0 | 0.0061 | 0 | 0.0076 | 0.0082 | 0 | 0.0124 | 0 | 0 | 0.006 | *0* | *0.0051* |
| A*24~DRB1*01:01 | 0 | 0.0102 | 0 | 0.0127 | 0.0248 | 0 | 0 | 0 | 0.0217 | 0.0231 | *0.0083* | *0.0093* |
| A*24~DRB1*01:02 | 0 | 0.0011 | 0 | 0 | 0 | 0 | 0 | 0 | 0 | 0.0107 | *0.0021* | *0.0012* |
| A*24~DRB1*03 | 0 | 0.0029 | 0.0146 | 0 | 0.0128 | 0 | 0.0051 | 0.0228 | 0 | 0.0134 | *0* | *0.0064* |
| A*24~DRB1*04 | 0 | 0.0113 | 0 | 0.0197 | 0.0123 | 0.0167 | 0.0043 | 0.0183 | 0.0228 | 0.0029 | *0.0117* | *0.0123* |
| A*24~DRB1*07 | 0.0161 | 0.0063 | 0.0213 | 0.0204 | 0 | 0.0124 | 0.0207 | 0.015 | 0.0217 | 0.0159 | *0.0229* | *0.0133* |
| A*24~DRB1*08 | 0 | 0.0043 | 0 | 0 | 0.0104 | 0 | 0 | 0 | 0.0071 | 0 | *0* | *0.0029* |
| A*24~DRB1*11 | 0.0513 | 0.0148 | 0.0223 | 0.0138 | 0.0325 | 0.0483 | 0.0148 | 0.0066 | 0.0258 | 0.034 | *0.024* | *0.0214* |
| A*24~DRB1*13:01 | 0 | 0.0074 | 0.0218 | 0 | 0.009 | 0 | 0.0062 | 0.0004 | 0 | 0 | *0.0086* | *0.0067* |
| A*24~DRB1*13:02 | 0 | 0.005 | 0 | 0.0051 | 0 | 0.0081 | 0.012 | 0.0115 | 0 | 0 | *0.0065* | *0.0044* |
| A*24~DRB1*14 | 0 | 0.0046 | 0 | 0 | 0 | 0.0217 | 0.0003 | 0 | 0 | 0 | *0* | *0.0025* |
| A*24~DRB1*15:01 | 0.0054 | 0.0219 | 0.016 | 0.0171 | 0.0044 | 0 | 0.0084 | 0 | 0 | 0.01 | *0.0187* | *0.0124* |
| A*25:01~DRB1*15:01 | 0 | 0.0046 | 0.0159 | 0 | 0 | 0.004 | 0.0041 | 0 | 0 | 0.0033 | *0.0048* | *0.0031* |
| A*26~DRB1*01:01 | 0.0141 | 0.0038 | 0 | 0 | 0.0041 | 0.0048 | 0.0046 | 0 | 0.009 | 0 | *0.0055* | *0.0041* |
| A*26~DRB1*03 | 0 | 0 | 0 | 0.0024 | 0.0145 | 0 | 0.0045 | 0 | 0 | 0 | *0* | *0.0019* |
| A*26~DRB1*07 | 0.0097 | 0.004 | 0 | 0.0155 | 0.0059 | 0 | 0.0099 | 0 | 0.0071 | 0 | *0.008* | *0.0059* |
| A*26~DRB1*08 | 0 | 0.0013 | 0 | 0 | 0.0043 | 0.0115 | 0.0019 | 0.0066 | 0 | 0 | *0* | *0.0008* |
| A*26~DRB1*11 | 0 | 0.0081 | 0.0222 | 0.0102 | 0 | 0.0121 | 0.0099 | 0.0185 | 0 | 0.022 | *0* | *0.0091* |
| A*26~DRB1*13:01 | 0 | 0.0051 | 0.004 | 0.0012 | 0 | 0.0073 | 0.0146 | 0.0058 | 0.0053 | 0.0074 | *0* | *0.0057* |
| A*26~DRB1*13:02 | 0 | 0.0031 | 0.0036 | 0.0012 | 0 | 0.004 | 0 | 0 | 0.0127 | 0 | *0.005* | *0.0019* |
| HAPLOTYPES | AA (n=103) | BE (n=709) | BS (n=126) | GE (n=105) | GR (n=228) | LG (n=124) | LS (n=364) | LU (n=151) | SG (n=111) | SI (n=153) | ZH (n=291) | All (n=2465) |
| A*26~DRB1*15:01 | 0.0053 | 0.0031 | 0 | 0.0046 | 0.0111 | 0 | 0 | 0.0066 | 0.0109 | 0 | *0* | *0.0036* |
| A*29~DRB1*04 | 0 | 0 | 0 | 0.0166 | 0.0025 | 0 | 0.0045 | 0 | 0 | 0 | *0* | *0.0023* |
| A*29~DRB1*07 | 0.0064 | 0.016 | 0.0159 | 0.031 | 0.0126 | 0.0089 | 0.0101 | 0.0208 | 0.006 | 0.0114 | *0.0139* | *0.0149* |
| A*29~DRB1*11 | 0.0097 | 0.003 | 0 | 0 | 0 | 0.0073 | 0 | 0 | 0.012 | 0 | *0.0073* | *0.0025* |
| A*30:01/24~DRB1*07 | 0.0146 | 0.0095 | 0.0027 | 0.0143 | 0.0058 | 0.002 | 0.0076 | 0.0295 | 0.0135 | 0.0117 | *0.0081* | *0.0127* |
| A*30:01/24~DRB1*15:01 | 0 | 0 | 0.0109 | 0.0048 | 0 | 0 | 0 | 0.0034 | 0 | 0 | *0* | *0.0013* |
| A*30:02~DRB1*03 | 0 | 0.0069 | 0 | 0.0381 | 0 | 0.0047 | 0.0006 | 0 | 0 | 0.0026 | *0.0086* | *0.0062* |
| A*31~DRB1*04 | 0 | 0.0105 | 0.0194 | 0 | 0.0085 | 0.0159 | 0.0093 | 0.0066 | 0.0055 | 0.0214 | *0.0251* | *0.012* |
| A*31~DRB1*11 | 0 | 0.007 | 0.0101 | 0.0286 | 0 | 0 | 0.0062 | 0 | 0 | 0.0157 | *0* | *0.0063* |
| A*31~DRB1*12 | 0 | 0 | 0.0119 | 0 | 0 | 0 | 0.0014 | 0 | 0 | 0 | *0* | *0.0005* |
| A*31~DRB1*13:02 | 0 | 0 | 0.0079 | 0 | 0.007 | 0 | 0 | 0 | 0.0121 | 0 | *0.003* | *0.0013* |
| A*31~DRB1*15:01 | 0.0071 | 0.0029 | 0.0102 | 0 | 0 | 0 | 0.0044 | 0 | 0.0135 | 0 | *0.0058* | *0.0051* |
| A*32:01~DRB1*01:01 | 0 | 0.0019 | 0 | 0.0048 | 0.0112 | 0 | 0 | 0.0021 | 0 | 0 | *0* | *0.0019* |
| A*32:01~DRB1*04 | 0.0097 | 0.0039 | 0.0141 | 0.0095 | 0.0071 | 0 | 0 | 0.0099 | 0 | 0 | *0.0045* | *0.004* |
| A*32:01~DRB1*07 | 0 | 0.0019 | 0 | 0 | 0.0034 | 0.0121 | 0.0122 | 0 | 0.0023 | 0.0065 | *0* | *0.004* |
| A*32:01~DRB1*11 | 0.0059 | 0.0171 | 0 | 0.0112 | 0.0177 | 0.0046 | 0.0133 | 0.0084 | 0 | 0.0103 | *0.0084* | *0.0118* |
| A*32:01~DRB1*12 | 0 | 0.0039 | 0 | 0 | 0.0022 | 0 | 0.0014 | 0 | 0.0135 | 0 | *0.0067* | *0.0034* |
| A*32:01~DRB1*13:02 | 0.0049 | 0.0031 | 0.004 | 0 | 0 | 0.0155 | 0 | 0 | 0.0023 | 0 | *0.0034* | *0.0022* |
| A*32:01~DRB1*14 | 0.0038 | 0.002 | 0 | 0 | 0 | 0 | 0.0017 | 0 | 0.0045 | 0.015 | *0* | *0.0022* |
| A*33~DRB1*01:02 | 0.0049 | 0.0014 | 0.0079 | 0.0048 | 0.0044 | 0.0161 | 0.0034 | 0 | 0.0045 | 0 | *0.012* | *0.005* |
| A*68~DRB1*01:01 | 0.0049 | 0.0103 | 0 | 0 | 0.0039 | 0 | 0 | 0.0099 | 0.009 | 0.0028 | *0.0032* | *0.0058* |
| A*68~DRB1*03 | 0.0146 | 0.003 | 0.004 | 0 | 0 | 0 | 0.0008 | 0.0079 | 0 | 0 | *0.0098* | *0.0028* |
| A*68~DRB1*04 | 0 | 0.0049 | 0.0118 | 0 | 0.0076 | 0 | 0 | 0 | 0.0182 | 0.0019 | *0.012* | *0.006* |
| A*68~DRB1*07 | 0 | 0.0039 | 0 | 0 | 0.0054 | 0.0086 | 0.0074 | 0 | 0.0154 | 0 | *0.0046* | *0.004* |
| A*68~DRB1*11 | 0.0194 | 0.0074 | 0 | 0.0095 | 0.0066 | 0.0276 | 0 | 0.0099 | 0 | 0.0324 | *0.0059* | *0.0099* |
| A*68~DRB1*13:01 | 0 | 0.0006 | 0.0091 | 0.0048 | 0 | 0.0151 | 0.0032 | 0 | 0.004 | 0.0098 | *0.0033* | *0.0026* |
| A*68~DRB1*13:02 | 0.0146 | 0.0036 | 0.0101 | 0 | 0 | 0.0162 | 0.0033 | 0.0132 | 0 | 0.0033 | *0.0075* | *0.0055* |
| A*68~DRB1*13:03 | 0 | 0 | 0 | 0 | 0.0108 | 0.0051 | 0 | 0 | 0.0074 | 0.0033 | *0* | *0.0027* |
| A*68~DRB1*15:01 | 0 | 0.0029 | 0.0143 | 0 | 0 | 0 | 0.0034 | 0.012 | 0 | 0.0124 | *0.0096* | *0.0054* |
| A*80:01~DRB1*08 | 0 | 0 | 0.0119 | 0 | 0 | 0 | 0 | 0 | 0 | 0 | *0* | *0* |
| blank~DRB1*03 | 0.0001 | 0 | 0.0068 | 0.0054 | 0.0029 | 0 | 0 | 0 | 0.0162 | 0 | *0.0036* | *0* |
| blank~DRB1*04 | 0.0087 | 0 | 0 | 0.0162 | 0 | 0.0062 | 0.0056 | 0 | 0 | 0.0083 | *0* | *0.0013* |
| blank~DRB1*13:01 | 0.01 | 0 | 0 | 0 | 0.0074 | 0 | 0 | 0 | 0 | 0 | *0* | *0* |
| blank~DRB1*14 | 0 | 0 | 0 | 0.0012 | 0 | 0 | 0.0035 | 0 | 0.0148 | 0 | *0* | *0.0006* |
| HAPLOTYPES | AA (n=103) | BE (n=709) | BS (n=126) | GE (n=105) | GR (n=228) | LG (n=124) | LS (n=364) | LU (n=151) | SG (n=111) | SI (n=153) | ZH (n=291) | All (n=2465) |
| blank~DRB1*15:01 | 0 | 0 | 0 | 0 | 0.0031 | 0 | 0 | 0 | 0 | 0.0134 | *0* | *0* |
| blank~DRB1*16 | 0 | 0 | 0 | 0 | 0.0008 | 0.0121 | 0.0013 | 0.004 | 0 | 0 | *0* | *0* |

**HLA-B-DRB1**

| HAPLOTYPES | AA (n=124) | BE (n=766) | BS (n=176) | GE (n=149) | GR (n=243) | LG (n=167) | LS (n=382) | LU (n=198) | SG (n=127) | SI (n=151) | ZH (n=416) | All (n=2899) |
| --- | --- | --- | --- | --- | --- | --- | --- | --- | --- | --- | --- | --- |
| B*07~DRB1*01:01 | 0 | 0.0054 | *0* | 0.0101 | 0.0106 | 0 | 0 | 0 | 0 | 0.0036 | *0.0044* | *0.0039* |
| B*07~DRB1*03 | 0 | 0.0009 | *0* | 0 | 0.0041 | 0 | 0.0019 | 0.0076 | 0.0118 | 0 | *0.0017* | *0.0026* |
| B*07~DRB1*04 | 0 | 0.0144 | *0.0199* | 0 | 0.0067 | 0.0119 | 0 | 0.0105 | 0.015 | 0.0095 | *0.0124* | *0.0108* |
| B*07~DRB1*07 | 0 | 0.0049 | *0.0142* | 0.0036 | 0.0055 | 0.003 | 0.0033 | 0 | 0.004 | 0 | *0.0057* | *0.004* |
| B*07~DRB1*08 | 0.0081 | 0.0018 | *0* | 0 | 0.0031 | 0 | 0 | 0.0151 | 0.0038 | 0.0053 | *0.0032* | *0.0037* |
| B*07~DRB1*11 | 0.0113 | 0.0153 | *0* | 0.0035 | 0 | 0.0032 | 0.0112 | 0 | 0 | 0.0074 | *0.0076* | *0.0076* |
| B*07~DRB1*14 | 0.004 | 0.0028 | *0.0028* | 0 | 0.0051 | 0.0031 | 0.0047 | 0.0143 | 0.0039 | 0 | *0.0037* | *0.0046* |
| B*07~DRB1*15:01 | 0.0651 | 0.0671 | *0.0455* | 0.0398 | 0.0538 | 0.0387 | 0.0655 | 0.0664 | 0.055 | 0.0488 | *0.0602* | *0.0591* |
| B*08~DRB1*03 | 0.0434 | 0.0604 | *0.0443* | 0.0503 | 0.0521 | 0.0433 | 0.0699 | 0.0421 | 0.0551 | 0.046 | *0.0442* | *0.0532* |
| B*08~DRB1*13:01 | 0 | 0 | *0* | 0.0034 | 0.0117 | 0 | 0.0045 | 0 | 0 | 0 | *0* | *0.0015* |
| B*08~DRB1*15:01 | 0 | 0.0117 | *0* | 0 | 0 | 0.0026 | 0.0067 | 0.0052 | 0 | 0.0099 | *0.0036* | *0.0053* |
| B*13:02~DRB1*07 | 0.0161 | 0.0202 | *0.0157* | 0.005 | 0.0226 | 0.0149 | 0.0152 | 0.0252 | 0.0236 | 0.0225 | *0.0179* | *0.0188* |
| B*14:02~DRB1*01:02 | 0.004 | 0.0046 | *0.0085* | 0.0235 | 0.0082 | 0.018 | 0.0104 | 0 | 0 | 0.0199 | *0.0105* | *0.0091* |
| B*14:02~DRB1*13:03 | 0.004 | 0.0013 | *0.0057* | 0.0017 | 0.0123 | 0.003 | 0 | 0 | 0 | 0.0033 | *0.0012* | *0.0027* |
| B*15~DRB1*01:01 | 0 | 0.0034 | *0.0052* | 0 | 0 | 0.012 | 0 | 0.0038 | 0 | 0.0066 | *0.0047* | *0.004* |
| B*15~DRB1*03 | 0 | 0.0007 | *0* | 0 | 0 | 0 | 0.002 | 0.0033 | 0 | 0.0103 | *0* | *0.001* |
| B*15~DRB1*04 | 0.0136 | 0.0218 | *0.0338* | 0.0115 | 0.0193 | 0.012 | 0.0071 | 0.0104 | 0.0221 | 0.0067 | *0.0184* | *0.0176* |
| B*15~DRB1*07 | 0.0136 | 0.003 | *0.0057* | 0.0076 | 0.0064 | 0.0021 | 0 | 0 | 0.0105 | 0.0033 | *0* | *0.0042* |
| B*15~DRB1*08 | 0.0193 | 0.0047 | *0* | 0.0101 | 0 | 0.004 | 0.0013 | 0.0066 | 0 | 0.0034 | *0.0037* | *0.0045* |
| B*15~DRB1*11 | 0.0025 | 0.011 | *0.0132* | 0.0264 | 0.0212 | 0.009 | 0.0121 | 0.0129 | 0.01 | 0.0381 | *0.0123* | *0.0135* |
| B*15~DRB1*13:01 | 0.0165 | 0.0158 | *0.0124* | 0.013 | 0.0085 | 0.015 | 0.0145 | 0.0034 | 0.0231 | 0 | *0.0234* | *0.0149* |
| B*15~DRB1*13:02 | 0.0121 | 0.0049 | *0.0265* | 0 | 0.0061 | 0.006 | 0.0059 | 0.0056 | 0.017 | 0.0038 | *0* | *0.006* |
| B*15~DRB1*15:01 | 0 | 0.0023 | *0* | 0.0068 | 0.0024 | 0.0058 | 0.0094 | 0.0136 | 0 | 0.0072 | *0.0069* | *0.0047* |
| B*18~DRB1*03 | 0.004 | 0.0151 | *0* | 0.019 | 0.0062 | 0.0057 | 0.0112 | 0.0141 | 0.0039 | 0 | *0.0051* | *0.0103* |
| B*18~DRB1*11 | 0.0161 | 0.0167 | *0.0157* | 0.0112 | 0.0186 | 0.0239 | 0.0155 | 0.0134 | 0.0079 | 0.0132 | *0.0052* | *0.0131* |
| B*18~DRB1*15:01 | 0 | 0.0072 | *0.0112* | 0 | 0.0055 | 0.0108 | 0.0048 | 0.0053 | 0 | 0 | *0.0067* | *0.0054* |
| B*27:05/13~DRB1*01:01 | 0 | 0.0079 | *0.0078* | 0 | 0.0031 | 0 | 0.0102 | 0 | 0.0004 | 0.0029 | *0.0047* | *0.0053* |
| B*27:05/13~DRB1*04 | 0 | 0.0089 | *0* | 0.0022 | 0.0038 | 0 | 0.0073 | 0 | 0.0151 | 0.0008 | *0.0017* | *0.0061* |
| B*27:05/13~DRB1*11 | 0.0032 | 0.0032 | *0* | 0 | 0 | 0.0035 | 0.0091 | 0.0088 | 0.014 | 0 | *0.005* | *0.0045* |
| B*35:01/40N/42/57/94~DRB1*01:01 | 0.0181 | 0.03 | *0.043* | 0.0235 | 0.0226 | 0.015 | 0.0152 | 0.0425 | 0.0197 | 0.0331 | *0.0295* | *0.0271* |
| B*35:01/40N/42/57/94~DRB1*04 | 0.0108 | 0.0051 | *0.0043* | 0 | 0 | 0 | 0.011 | 0.0123 | 0.0039 | 0 | *0.0077* | *0.0058* |
| B*35:01/40N/42/57/94~DRB1*08 | 0 | 0.0059 | *0* | 0.0101 | 0 | 0 | 0 | 0 | 0.0002 | 0.0004 | *0.0036* | *0.0029* |
| HAPLOTYPES | AA (n=124) | BE (n=766) | BS (n=176) | GE (n=149) | GR (n=243) | LG (n=167) | LS (n=382) | LU (n=198) | SG (n=127) | SI (n=151) | ZH (n=416) | All (n=2899) |
| B*35:01/40N/42/57/94~DRB1*11 | 0.0096 | 0.0076 | *0.0095* | 0.0045 | 0 | 0.0253 | 0.0016 | 0 | 0 | 0 | *0* | *0.0056* |
| B*35:02~DRB1*04 | 0 | 0.0007 | *0.0043* | 0 | 0 | 0 | 0 | 0 | 0 | 0.011 | *0* | *0.0012* |
| B*35:02~DRB1*11 | 0 | 0.006 | *0.0057* | 0.027 | 0 | 0.0046 | 0.0065 | 0.0251 | 0.0066 | 0 | *0.0094* | *0.0081* |
| B*35:02~DRB1*13:02 | 0 | 0 | *0.0028* | 0 | 0.0021 | 0.0002 | 0 | 0 | 0.0118 | 0 | *0.0019* | *0.0011* |
| B*35:03/70~DRB1*04 | 0 | 0.0011 | *0* | 0.0093 | 0.0107 | 0.0142 | 0.0024 | 0.0059 | 0.0157 | 0 | *0.0035* | *0.0033* |
| B*35:03/70~DRB1*08 | 0.0067 | 0.0004 | *0.017* | 0 | 0 | 0.0034 | 0.0007 | 0.0013 | 0.0052 | 0.0004 | *0* | *0.0031* |
| B*35:03/70~DRB1*11 | 0.0159 | 0.0027 | *0* | 0.0062 | 0.0038 | 0.0044 | 0.0109 | 0 | 0.0131 | 0 | *0.0193* | *0.0068* |
| B*35:06~DRB1*11 | 0 | 0 | *0* | 0 | 0 | 0.011 | 0 | 0 | 0 | 0 | *0* | *0* |
| B*35:08~DRB1*15:01 | 0 | 0 | *0.0038* | 0 | 0.0135 | 0 | 0 | 0 | 0 | 0 | *0* | *0.0001* |
| B*38:01~DRB1*13:01 | 0 | 0.0041 | *0.0156* | 0.0034 | 0.0018 | 0.006 | 0.0082 | 0 | 0.0118 | 0.0081 | *0.0069* | *0.0068* |
| B*39:01~DRB1*01:01 | 0.004 | 0 | *0.0028* | 0 | 0 | 0 | 0 | 0.001 | 0.0118 | 0 | *0.0017* | *0.0013* |
| B*39:01~DRB1*12 | 0 | 0 | *0.0002* | 0 | 0 | 0 | 0 | 0 | 0.0118 | 0 | *0.0002* | *0.0008* |
| B*39:06~DRB1*11 | 0.004 | 0 | *0* | 0.011 | 0 | 0 | 0 | 0 | 0 | 0.0033 | *0* | *0.0004* |
| B*40:01/55~blank | 0.0086 | 0 | *0* | 0.0039 | 0 | 0 | 0 | 0 | 0 | 0.0121 | *0* | *0* |
| B*40:01/55~DRB1*04 | 0 | 0.0163 | *0.0153* | 0 | 0.0153 | 0 | 0.0132 | 0.0126 | 0 | 0.0201 | *0.019* | *0.0129* |
| B*40:01/55~DRB1*13:02 | 0.0137 | 0.0167 | *0.0094* | 0.0147 | 0.0037 | 0.003 | 0.0161 | 0.0051 | 0.0182 | 0.0066 | *0.0113* | *0.0128* |
| B*41:02~DRB1*13:03 | 0.0121 | 0.0039 | *0.0028* | 0.0017 | 0 | 0 | 0 | 0.0051 | 0.0072 | 0.0033 | *0.0024* | *0.0031* |
| B*44:02/19N/27~DRB1*01:01 | 0.021 | 0.0032 | *0.0044* | 0.0134 | 0.0122 | 0 | 0 | 0 | 0.0118 | 0.003 | *0.0045* | *0.0053* |
| B*44:02/19N/27~DRB1*04 | 0.0053 | 0.0253 | *0.0102* | 0.0101 | 0.0365 | 0.011 | 0.0211 | 0.0129 | 0.0272 | 0.0115 | *0.0137* | *0.0188* |
| B*44:02/19N/27~DRB1*07 | 0 | 0.0016 | *0* | 0 | 0 | 0 | 0.0021 | 0.0032 | 0.0125 | 0.009 | *0.0015* | *0.0026* |
| B*44:02/19N/27~DRB1*11 | 0.0017 | 0.0225 | *0.0199* | 0.0245 | 0.0256 | 0 | 0.0145 | 0.0078 | 0.0066 | 0.0237 | *0.0131* | *0.0165* |
| B*44:02/19N/27~DRB1*12 | 0.0005 | 0.006 | *0.0114* | 0 | 0.0021 | 0.003 | 0.0062 | 0.0025 | 0 | 0.0033 | *0.0057* | *0.0047* |
| B*44:02/19N/27~DRB1*13:01 | 0 | 0.0113 | *0.0057* | 0 | 0 | 0.009 | 0.0025 | 0.0051 | 0 | 0 | *0.0078* | *0.007* |
| B*44:02/19N/27~DRB1*15:01 | 0.0137 | 0.0018 | *0* | 0 | 0 | 0 | 0.002 | 0 | 0.004 | 0.0036 | *0.0104* | *0.0033* |
| B*44:03~DRB1*01:01 | 0 | 0 | *0* | 0 | 0.0114 | 0.006 | 0 | 0.0055 | 0 | 0.0033 | *0* | *0.0024* |
| B*44:03~DRB1*07 | 0.0476 | 0.0375 | *0.0254* | 0.0493 | 0.0251 | 0.0215 | 0.0493 | 0.0564 | 0.011 | 0.029 | *0.0347* | *0.0365* |
| B*44:03~DRB1*11 | 0 | 0.0009 | *0* | 0 | 0 | 0 | 0 | 0.0033 | 0.0121 | 0 | *0.003* | *0.0016* |
| B*45~DRB1*07 | 0 | 0.0006 | *0* | 0.0034 | 0 | 0 | 0.0026 | 0 | 0 | 0.0132 | *0* | *0.0013* |
| B*49:01~DRB1*11 | 0 | 0.0098 | *0* | 0.01 | 0.0052 | 0.012 | 0.0055 | 0.0173 | 0 | 0.0066 | *0.006* | *0.007* |
| B*49:01~DRB1*13:02 | 0 | 0.0043 | *0.0027* | 0.0101 | 0 | 0 | 0.0012 | 0.0046 | 0 | 0 | *0.0024* | *0.003* |
| B*50:01~DRB1*07 | 0.0161 | 0.0054 | *0* | 0.0134 | 0.0082 | 0.003 | 0.0108 | 0.0082 | 0.0079 | 0 | *0.0084* | *0.0086* |
| B*51~DRB1*01:01 | 0.0119 | 0.0068 | *0* | 0.0045 | 0.0129 | 0 | 0.0054 | 0.0018 | 0.0157 | 0 | *0.0074* | *0.0069* |
| B*51~DRB1*04 | 0.004 | 0.0132 | *0.0057* | 0.0101 | 0.0051 | 0.0062 | 0.0225 | 0.0207 | 0.0153 | 0.0283 | *0.0171* | *0.0138* |
| B*51~DRB1*07 | 0 | 0.0052 | *0* | 0.0044 | 0.0155 | 0.0079 | 0.009 | 0 | 0 | 0.0039 | *0.0016* | *0.0056* |
| HAPLOTYPES | AA (n=124) | BE (n=766) | BS (n=176) | GE (n=149) | GR (n=243) | LG (n=167) | LS (n=382) | LU (n=198) | SG (n=127) | SI (n=151) | ZH (n=416) | All (n=2899) |
| B*51~DRB1*08 | 0.0094 | 0.0091 | *0* | 0.0034 | 0.0076 | 0.0285 | 0 | 0.006 | 0.0111 | 0 | *0.0074* | *0.0069* |
| B*51~DRB1*11 | 0.0453 | 0.0208 | *0.0185* | 0.0177 | 0.043 | 0.0749 | 0.02 | 0.0198 | 0.0225 | 0.0507 | *0.0209* | *0.0271* |
| B*51~DRB1*13:01 | 0.0208 | 0.0103 | *0.0092* | 0 | 0.014 | 0.018 | 0.0073 | 0.0067 | 0.0084 | 0.0298 | *0.0119* | *0.0125* |
| B*51~DRB1*14 | 0 | 0.0022 | *0* | 0 | 0.0003 | 0.0202 | 0.0057 | 0.0056 | 0.0157 | 0 | *0.0045* | *0.0055* |
| B*51~DRB1*15:01 | 0.0123 | 0.011 | *0.0114* | 0.0036 | 0.0182 | 0 | 0 | 0 | 0 | 0 | *0.0055* | *0.0054* |
| B*51~DRB1*16 | 0 | 0.0013 | *0* | 0.0034 | 0 | 0.015 | 0.0062 | 0 | 0 | 0.0017 | *0* | *0.0025* |
| B*52:01/07~DRB1*15:02 | 0.0081 | 0.0072 | *0.0085* | 0.0201 | 0.0007 | 0.009 | 0.0039 | 0.0025 | 0.0079 | 0.0017 | *0.0096* | *0.0072* |
| B*53:01~DRB1*11 | 0.0138 | 0 | *0.0085* | 0 | 0 | 0 | 0 | 0 | 0.001 | 0 | *0.0008* | *0.0021* |
| B*53:01~DRB1*13:02 | 0.0103 | 0.0096 | *0.0085* | 0.0268 | 0.004 | 0.012 | 0 | 0.0038 | 0.0079 | 0.0033 | *0.0051* | *0.0093* |
| B*55~DRB1*04 | 0.004 | 0.0013 | *0.0081* | 0.0101 | 0 | 0.003 | 0 | 0.0119 | 0 | 0 | *0.0028* | *0.0029* |
| B*55~DRB1*11 | 0 | 0.0008 | *0.0026* | 0.0034 | 0.0041 | 0.0046 | 0.0013 | 0.0058 | 0.0236 | 0.0046 | *0* | *0.0029* |
| B*55~DRB1*14 | 0 | 0.005 | *0.0085* | 0.0201 | 0 | 0 | 0.009 | 0.0051 | 0.0039 | 0.0099 | *0.0063* | *0.0058* |
| B*55~DRB1*16 | 0.0121 | 0 | *0* | 0 | 0.0041 | 0.0014 | 0.0026 | 0.002 | 0 | 0.0021 | *0.0024* | *0.0019* |
| B*57:01~DRB1*07 | 0.0503 | 0.0223 | *0.0426* | 0.0319 | 0.0164 | 0.0256 | 0.0302 | 0.0426 | 0.0471 | 0.0166 | *0.0328* | *0.0289* |
| B*58:01/11~DRB1*13:02 | 0 | 0.005 | *0.0108* | 0.0137 | 0.0096 | 0.0144 | 0.0065 | 0.0097 | 0.0079 | 0 | *0.01* | *0.0076* |
| B*58:01/11~DRB1*14 | 0.0081 | 0 | *0.0114* | 0 | 0 | 0 | 0 | 0 | 0 | 0 | *0.0012* | *0.0003* |
| blank~DRB1*03 | 0 | 0 | *0.0047* | 0 | 0 | 0.0121 | 0 | 0 | 0 | 0.0066 | *0.0024* | *0.0013* |
| blank~DRB1*04 | 0.0097 | 0 | *0* | 0.0163 | 0.0085 | 0 | 0 | 0 | 0.007 | 0 | *0* | *0.0011* |
| blank~DRB1*11 | 0 | 0.0011 | *0.0116* | 0 | 0.0055 | 0 | 0 | 0 | 0 | 0 | *0.0084* | *0.001* |

**HLA-C-DRB1**

| HAPLOTYPES | AA (n=291) | BE (n=751) | BS (n=341) | GE (n=200) | GR (n=209) | LG (n=218) | LS (n=154) | LU (n=292) | SG (n=316) | SI (n=101) | ZH (n=639) | All (n=3512) |
| --- | --- | --- | --- | --- | --- | --- | --- | --- | --- | --- | --- | --- |
| C*01:02~DRB1*01:01 | 0.0099 | 0.0126 | 0.0034 | 0 | 0 | 0.0071 | 0.0085 | 0 | 0.0091 | 0.0097 | *0.0138* | *0.0086* |
| C*01:02~DRB1*11 | 0.01 | 0.0069 | 0.0085 | 0.0271 | 0.0172 | 0.0152 | 0.0208 | 0.0097 | 0.002 | 0.005 | *0.0112* | *0.0109* |
| C*01:02~DRB1*15:01 | 0.007 | 0.0043 | 0 | 0 | 0.0138 | 0 | 0 | 0 | 0.0071 | 0.0051 | *0* | *0.0025* |
| C*02:02~DRB1*01:01 | 0.0058 | 0.0064 | 0.0107 | 0.0016 | 0.0051 | 0 | 0.013 | 0.0058 | 0.0021 | 0.0099 | *0.0072* | *0.0065* |
| C*02:02~DRB1*04 | 0.0188 | 0.0053 | 0.0044 | 0.0025 | 0.0171 | 0.0089 | 0.0097 | 0.0049 | 0.0094 | 0 | *0.0092* | *0.0083* |
| C*02:02~DRB1*11 | 0.0082 | 0.0165 | 0.0021 | 0.0106 | 0.0062 | 0.0034 | 0.0162 | 0.0154 | 0.0174 | 0 | *0.0099* | *0.0122* |
| C*02:02~DRB1*16 | 0 | 0.0055 | 0.0044 | 0 | 0.0003 | 0.0134 | 0 | 0.0028 | 0.0043 | 0.005 | *0.0008* | *0.0043* |
| C*03:03/20N~DRB1*04 | 0.007 | 0.0086 | 0.0149 | 0.0053 | 0.0012 | 0.0027 | 0.0204 | 0.0081 | 0.0024 | 0 | *0.0069* | *0.008* |
| C*03:03/20N~DRB1*11 | 0 | 0.0124 | 0.0111 | 0.0155 | 0.0214 | 0.0045 | 0 | 0 | 0.019 | 0.0137 | *0.0104* | *0.0107* |
| C*03:03/20N~DRB1*13:01 | 0.0252 | 0.0131 | 0.0187 | 0.0101 | 0.0053 | 0.008 | 0.013 | 0.0048 | 0.0097 | 0.0149 | *0.0163* | *0.0136* |
| C*03:03/20N~DRB1*14 | 0.007 | 0.0097 | 0.0048 | 0.0091 | 0 | 0.002 | 0 | 0.0051 | 0.0057 | 0.0135 | *0.0045* | *0.0067* |
| C*03:04~DRB1*04 | 0.0194 | 0.0216 | 0.028 | 0.0204 | 0.0031 | 0 | 0.0153 | 0.0173 | 0.0199 | 0.004 | *0.02* | *0.0204* |
| C*03:04~DRB1*11 | 0.0063 | 0.0022 | 0.0079 | 0 | 0.0015 | 0.0106 | 0 | 0.0005 | 0.0013 | 0.004 | *0.0027* | *0.005* |
| C*03:04~DRB1*13:02 | 0.023 | 0.0134 | 0.0152 | 0.0091 | 0 | 0 | 0.0009 | 0.0012 | 0.0184 | 0.0004 | *0.0152* | *0.0124* |
| C*04:01/09N/28/30~DRB1*01:01 | 0.0334 | 0.0378 | 0.0412 | 0.0211 | 0.0298 | 0.0261 | 0.0196 | 0.0422 | 0.0269 | 0.0248 | *0.0368* | *0.0331* |
| C*04:01/09N/28/30~DRB1*03 | 0.0076 | 0.0071 | 0.0086 | 0 | 0.0105 | 0.0087 | 0.0041 | 0.0039 | 0.0017 | 0.0052 | *0.0059* | *0.0055* |
| C*04:01/09N/28/30~DRB1*04 | 0.0111 | 0.0099 | 0.0177 | 0.0159 | 0.0166 | 0.0164 | 0.0233 | 0.0108 | 0.024 | 0.0088 | *0.0148* | *0.0146* |
| C*04:01/09N/28/30~DRB1*07 | 0.0254 | 0.02 | 0.0137 | 0.0114 | 0 | 0.0088 | 0.0231 | 0.0176 | 0.0087 | 0.0064 | *0.0134* | *0.0162* |
| C*04:01/09N/28/30~DRB1*08 | 0.0064 | 0.0066 | 0.0087 | 0.0048 | 0 | 0.0069 | 0.0065 | 0.0273 | 0.0028 | 0.0017 | *0.0065* | *0.0079* |
| C*04:01/09N/28/30~DRB1*11 | 0.0211 | 0.0141 | 0.0271 | 0.0275 | 0.0135 | 0.0359 | 0.0209 | 0.0212 | 0.0257 | 0.0779 | *0.0199* | *0.0209* |
| C*04:01/09N/28/30~DRB1*13:01 | 0.008 | 0.0095 | 0 | 0.0189 | 0 | 0.0036 | 0 | 0 | 0 | 0 | *0.0074* | *0.0053* |
| C*04:01/09N/28/30~DRB1*13:02 | 0.0035 | 0.0023 | 0.0085 | 0 | 0 | 0.0161 | 0.0097 | 0.0031 | 0.0061 | 0 | *0.0075* | *0.0048* |
| C*04:01/09N/28/30~DRB1*14 | 0.0033 | 0.0098 | 0.0039 | 0.0009 | 0.0055 | 0.0034 | 0.0132 | 0.0017 | 0.0047 | 0 | *0.0058* | *0.0067* |
| C*04:01/09N/28/30~DRB1*15:01 | 0.0175 | 0.0133 | 0.0066 | 0 | 0.0339 | 0.0099 | 0.0029 | 0.0094 | 0 | 0 | *0.0084* | *0.0096* |
| C*05:01/03~DRB1*01:01 | 0.017 | 0.0035 | 0.0037 | 0.0108 | 0.017 | 0 | 0 | 0 | 0.0108 | 0 | *0.0114* | *0.007* |
| C*05:01/03~DRB1*03 | 0.0098 | 0.0124 | 0.0083 | 0.0253 | 0 | 0.0051 | 0.0114 | 0.0138 | 0.0143 | 0.0146 | *0.0041* | *0.0109* |
| C*05:01/03~DRB1*04 | 0.0171 | 0.0216 | 0.0233 | 0.0213 | 0.0215 | 0.0301 | 0 | 0.0354 | 0.0351 | 0.0245 | *0.0296* | *0.0243* |
| C*05:01/03~DRB1*07 | 0 | 0.0033 | 0 | 0 | 0.006 | 0.0032 | 0.0041 | 0.0071 | 0.0004 | 0.0111 | *0* | *0.0024* |
| C*05:01/03~DRB1*11 | 0.0065 | 0.0187 | 0.0138 | 0.0078 | 0.0153 | 0.0218 | 0.0311 | 0.0026 | 0.0033 | 0.0104 | *0.0071* | *0.0121* |
| C*05:01/03~DRB1*13:01 | 0.014 | 0.0102 | 0.0123 | 0.0116 | 0.0048 | 0.0082 | 0.0139 | 0.0172 | 0.0112 | 0 | *0.0146* | *0.0113* |
| C*05:01/03~DRB1*15:01 | 0.0038 | 0.0047 | 0.0037 | 0.0027 | 0 | 0 | 0.0097 | 0 | 0 | 0 | *0.0105* | *0.005* |
| C*06:02~DRB1*03 | 0 | 0 | 0 | 0 | 0.0072 | 0 | 0 | 0 | 0 | 0.0112 | *0.0042* | *0.001* |
| HAPLOTYPES | AA (n=291) | BE (n=751) | BS (n=341) | GE (n=200) | GR (n=209) | LG (n=218) | LS (n=154) | LU (n=292) | SG (n=316) | SI (n=101) | ZH (n=639) | All (n=3512) |
| C*06:02~DRB1*04 | 0 | 0.0077 | 0.0104 | 0.01 | 0.0037 | 0.0084 | 0.0141 | 0.0069 | 0 | 0.0131 | *0.0064* | *0.0067* |
| C*06:02~DRB1*07 | 0.0657 | 0.0469 | 0.0429 | 0.0398 | 0.0707 | 0.0444 | 0.0473 | 0.0667 | 0.081 | 0.0555 | *0.0551* | *0.0546* |
| C*06:02~DRB1*08 | 0 | 0.0011 | 0.001 | 0 | 0.0114 | 0 | 0 | 0 | 0.0016 | 0 | *0* | *0.0012* |
| C*06:02~DRB1*11 | 0.0062 | 0.0079 | 0.0087 | 0.0035 | 0.0078 | 0.0054 | 0.0068 | 0.0161 | 0.007 | 0 | *0.0071* | *0.0075* |
| C*06:02~DRB1*13:01 | 0.0053 | 0.0038 | 0.0063 | 0.0041 | 0 | 0.0125 | 0 | 0 | 0.0022 | 0.0149 | *0.0017* | *0.0038* |
| C*06:02~DRB1*15:01 | 0 | 0.0053 | 0.0025 | 0.0101 | 0.0099 | 0 | 0 | 0.0022 | 0.0026 | 0 | *0* | *0.0043* |
| C*07:01/06/18/52~DRB1*03 | 0.0535 | 0.0566 | 0.0608 | 0.0598 | 0.0492 | 0.0498 | 0.0882 | 0.0481 | 0.0555 | 0.0515 | *0.0576* | *0.0569* |
| C*07:01/06/18/52~DRB1*04 | 0.0159 | 0.0086 | 0.0086 | 0.0012 | 0.0044 | 0 | 0.0138 | 0 | 0.0063 | 0.0221 | *0.0143* | *0.0093* |
| C*07:01/06/18/52~DRB1*07 | 0.0031 | 0.0054 | 0.0072 | 0.005 | 0 | 0.0103 | 0.0062 | 0 | 0.0092 | 0 | *0.0106* | *0.0065* |
| C*07:01/06/18/52~DRB1*11 | 0.0342 | 0.0294 | 0.017 | 0.0176 | 0.0104 | 0.0288 | 0.0081 | 0.0438 | 0.0205 | 0.0105 | *0.0218* | *0.0249* |
| C*07:01/06/18/52~DRB1*13:01 | 0.0048 | 0.001 | 0.0086 | 0.0017 | 0.0135 | 0.011 | 0.0059 | 0.0022 | 0.0042 | 0.0149 | *0* | *0.0058* |
| C*07:01/06/18/52~DRB1*13:02 | 0 | 0.0102 | 0.0125 | 0.0129 | 0.0055 | 0.0041 | 0.0062 | 0.0048 | 0.0022 | 0.0074 | *0* | *0.0077* |
| C*07:01/06/18/52~DRB1*14 | 0.0114 | 0.0024 | 0.0035 | 0 | 0.0012 | 0.0217 | 0 | 0 | 0.0009 | 0 | *0.0059* | *0.0041* |
| C*07:01/06/18/52~DRB1*15:01 | 0 | 0.0153 | 0 | 0.0167 | 0.0031 | 0.0131 | 0.0203 | 0.0191 | 0.0069 | 0.0169 | *0.0071* | *0.0099* |
| C*07:02/50~DRB1*01:01 | 0 | 0.0086 | 0.0075 | 0.0223 | 0.014 | 0.0034 | 0 | 0.0045 | 0.0188 | 0 | *0.0026* | *0.0082* |
| C*07:02/50~DRB1*03 | 0.0106 | 0.004 | 0.0037 | 0.0093 | 0 | 0 | 0 | 0.0039 | 0.0076 | 0 | *0* | *0.0054* |
| C*07:02/50~DRB1*04 | 0.0045 | 0.0141 | 0.0154 | 0.0099 | 0.0086 | 0.0092 | 0 | 0.0134 | 0.0144 | 0.0114 | *0.0161* | *0.0116* |
| C*07:02/50~DRB1*07 | 0.0037 | 0.0037 | 0.0027 | 0 | 0.0104 | 0.0072 | 0 | 0.0019 | 0 | 0 | *0.0062* | *0.0043* |
| C*07:02/50~DRB1*08 | 0.0143 | 0.0029 | 0.0019 | 0 | 0.007 | 0.0044 | 0 | 0.0143 | 0.0081 | 0.0099 | *0.0094* | *0.0075* |
| C*07:02/50~DRB1*11 | 0.0159 | 0.0106 | 0.0008 | 0.0068 | 0 | 0.0166 | 0.022 | 0 | 0.0035 | 0.0101 | *0.0088* | *0.0082* |
| C*07:02/50~DRB1*13:01 | 0.0027 | 0.01 | 0 | 0 | 0 | 0 | 0.0157 | 0.0023 | 0.006 | 0 | *0.0024* | *0.0041* |
| C*07:02/50~DRB1*13:02 | 0.0087 | 0.007 | 0.0021 | 0 | 0.0148 | 0.0014 | 0 | 0.0039 | 0.005 | 0 | *0.0059* | *0.0045* |
| C*07:02/50~DRB1*14 | 0.0086 | 0.0044 | 0.014 | 0.0026 | 0.0024 | 0 | 0.0054 | 0 | 0 | 0.0093 | *0.0064* | *0.0055* |
| C*07:02/50~DRB1*15:01 | 0.0543 | 0.0661 | 0.0571 | 0.0559 | 0.0816 | 0.028 | 0.0403 | 0.0634 | 0.0499 | 0.0407 | *0.0593* | *0.0575* |
| C*07:04/11~DRB1*11 | 0.0052 | 0.0096 | 0.0085 | 0.0092 | 0.0036 | 0.011 | 0 | 0.0036 | 0.0116 | 0.0185 | *0.006* | *0.008* |
| C*08:02~DRB1*01:02 | 0.0086 | 0.0073 | 0.0044 | 0.0225 | 0.0096 | 0.0183 | 0.0065 | 0.0051 | 0.0111 | 0.0149 | *0.0085* | *0.0093* |
| C*08:02~DRB1*07 | 0.0034 | 0.0016 | 0.0059 | 0.0142 | 0.0118 | 0.0048 | 0.0065 | 0.0074 | 0.0036 | 0 | *0.007* | *0.0058* |
| C*12:02~DRB1*15:02 | 0.0052 | 0.0069 | 0 | 0.01 | 0.0024 | 0.0025 | 0.0065 | 0.0034 | 0.0063 | 0 | *0.0074* | *0.0055* |
| C*12:03~DRB1*01:01 | 0.0039 | 0.0024 | 0.0022 | 0.0132 | 0.008 | 0 | 0.0032 | 0.0036 | 0.01 | 0 | *0.0022* | *0.0042* |
| C*12:03~DRB1*11 | 0.0158 | 0.012 | 0.0217 | 0.0213 | 0.0092 | 0.0154 | 0.0236 | 0.02 | 0.015 | 0.0177 | *0.0167* | *0.0149* |
| C*12:03~DRB1*13:01 | 0 | 0.0056 | 0.0125 | 0.0101 | 0.0022 | 0.0137 | 0.013 | 0.0013 | 0.01 | 0.01 | *0.011* | *0.0082* |
| C*12:03~DRB1*15:01 | 0 | 0.0052 | 0.0097 | 0 | 0 | 0.0141 | 0 | 0.0025 | 0 | 0 | *0.0035* | *0.004* |
| C*14:02~DRB1*08 | 0.0011 | 0.0037 | 0.0009 | 0 | 0.0008 | 0.0112 | 0 | 0 | 0 | 0 | *0.0039* | *0.0029* |
| C*14:02~DRB1*11 | 0.0103 | 0 | 0.0011 | 0.0025 | 0.0072 | 0.0031 | 0.0097 | 0.0073 | 0.0086 | 0 | *0.0089* | *0.006* |
| HAPLOTYPES | AA (n=291) | BE (n=751) | BS (n=341) | GE (n=200) | GR (n=209) | LG (n=218) | LS (n=154) | LU (n=292) | SG (n=316) | SI (n=101) | ZH (n=639) | All (n=3512) |
| C*15:02/13~DRB1*04 | 0.0054 | 0.0098 | 0.0074 | 0.0064 | 0.0027 | 0.0042 | 0.0177 | 0.0067 | 0.0115 | 0.005 | *0.0186* | *0.01* |
| C*15:02/13~DRB1*11 | 0.0163 | 0.0098 | 0.0085 | 0.0211 | 0.0047 | 0.0068 | 0.0001 | 0.0073 | 0.0095 | 0.017 | *0.0082* | *0.0108* |
| C*15:02/13~DRB1*13:01 | 0.0016 | 0.0057 | 0.002 | 0.0059 | 0 | 0.002 | 0 | 0.0024 | 0.0056 | 0.0127 | *0.0051* | *0.0042* |
| C*15:02/13~DRB1*14 | 0.0009 | 0.0032 | 0 | 0 | 0 | 0.0146 | 0.0005 | 0.0009 | 0 | 0 | *0.001* | *0.002* |
| C*15:02/13~DRB1*15:01 | 0.0105 | 0.0057 | 0.004 | 0.0024 | 0.0027 | 0 | 0.007 | 0.0035 | 0.0115 | 0 | *0.0029* | *0.0048* |
| C*16:01~DRB1*01:01 | 0 | 0 | 0 | 0 | 0.0052 | 0.0046 | 0.0038 | 0 | 0.0017 | 0.0146 | *0.0009* | *0.0013* |
| C*16:01~DRB1*07 | 0.016 | 0.029 | 0.0293 | 0.0359 | 0.0217 | 0.0159 | 0.0338 | 0.0373 | 0.0138 | 0.0247 | *0.0256* | *0.0259* |

**HLA-DRB1-DQB1**

| HAPLOTYPES | AA (n=108) | BE (n=661) | BS (n=148) | GE (n=97) | GR (n=40) | LG (n=99) | LS (n=74) | LU (n=92) | SG (n=135) | SI (n=62) | ZH (n=292) | All (n=1808) |
| --- | --- | --- | --- | --- | --- | --- | --- | --- | --- | --- | --- | --- |
| blank~blank | 0 | 0 | 0.0001 | 0 | 0.0297 | 0 | 0 | 0 | 0.0107 | 0 | *0* | *0* |
| DRB1*01:01~DQB1*03:01/09/19/21 | 0 | 0 | 0 | 0 | 0.0125 | 0 | 0 | 0 | 0 | 0 | *0* | *0.0003* |
| DRB1*01:01~DQB1*05:01 | 0.0828 | 0.0771 | 0.1115 | 0.0991 | 0.06 | 0.0556 | 0.0608 | 0.0585 | 0.088 | 0.0726 | *0.0803* | *0.0793* |
| DRB1*01:01~DQB1*05:04 | 0 | 0.0015 | 0 | 0 | 0.015 | 0 | 0.0068 | 0 | 0.0045 | 0 | *0.0054* | *0.0029* |
| DRB1*01:02~DQB1*05:01 | 0.0097 | 0.0061 | 0.0135 | 0.0124 | 0.0088 | 0.0152 | 0.0009 | 0.0176 | 0.0074 | 0.0161 | *0.0086* | *0.0096* |
| DRB1*01:03~DQB1*05:01 | 0 | 0.003 | 0 | 0.0117 | 0 | 0.0051 | 0 | 0 | 0 | 0 | *0.0017* | *0.0022* |
| DRB1*03~DQB1*02:01 | 0.0547 | 0.0862 | 0.0574 | 0.1134 | 0.1081 | 0.0909 | 0.1014 | 0.0761 | 0.077 | 0.0968 | *0.0719* | *0.0817* |
| DRB1*04~DQB1*03:01/09/19/21 | 0.0301 | 0.0345 | 0.039 | 0.0239 | 0.0525 | 0.033 | 0.0303 | 0.045 | 0.0457 | 0.0444 | *0.0493* | *0.0381* |
| DRB1*04~DQB1*03:02 | 0.0903 | 0.0912 | 0.0923 | 0.0895 | 0.035 | 0.0462 | 0.1588 | 0.1069 | 0.0939 | 0.0444 | *0.1128* | *0.0933* |
| DRB1*07~DQB1*02:01 | 0 | 0.001 | 0.0257 | 0 | 0 | 0.0253 | 0.024 | 0.017 | 0.0103 | 0 | *0.0111* | *0.0082* |
| DRB1*07~DQB1*02:02 | 0.0972 | 0.098 | 0.0824 | 0.0928 | 0.1337 | 0.0758 | 0.084 | 0.1189 | 0.0958 | 0.0887 | *0.0946* | *0.0958* |
| DRB1*07~DQB1*03:03 | 0.0417 | 0.0288 | 0.0405 | 0.0309 | 0.025 | 0.0404 | 0.0406 | 0.0272 | 0.0444 | 0.0161 | *0.0276* | *0.032* |
| DRB1*08~DQB1*03:01/09/19/21 | 0 | 0.0038 | 0.0034 | 0 | 0 | 0.0051 | 0.0135 | 0.0054 | 0.0037 | 0 | *0.0029* | *0.0036* |
| DRB1*08~DQB1*04:02 | 0.0463 | 0.0348 | 0.0371 | 0.0206 | 0.0125 | 0.0505 | 0.027 | 0.0489 | 0.047 | 0.0242 | *0.0223* | *0.0342* |
| DRB1*09:01~DQB1*03:03 | 0.0046 | 0.0098 | 0.0101 | 0 | 0 | 0 | 0.0068 | 0 | 0.0111 | 0.0242 | *0.0051* | *0.0075* |
| DRB1*10:01~DQB1*05:01 | 0.0139 | 0.0091 | 0.0034 | 0 | 0 | 0.0152 | 0.0135 | 0.0109 | 0 | 0 | *0.0034* | *0.0069* |
| DRB1*11~blank | 0 | 0.0009 | 0 | 0 | 0 | 0.0126 | 0 | 0 | 0 | 0 | *0* | *0* |
| DRB1*11~DQB1*03:01/09/19/21 | 0.162 | 0.1571 | 0.1047 | 0.1959 | 0.2069 | 0.1793 | 0.1824 | 0.1685 | 0.1444 | 0.1935 | *0.1438* | *0.1576* |
| DRB1*12~DQB1*03:01/09/19/21 | 0.0046 | 0.015 | 0.0169 | 0.0103 | 0 | 0.0051 | 0.0068 | 0.0054 | 0.0222 | 0.0161 | *0.0154* | *0.0133* |
| DRB1*13:01~DQB1*06:01 | 0 | 0 | 0 | 0 | 0.0142 | 0 | 0 | 0 | 0 | 0 | *0* | *0.0003* |
| DRB1*13:01~DQB1*06:03 | 0.0926 | 0.0647 | 0.0878 | 0.0515 | 0.0882 | 0.0657 | 0.0728 | 0.0707 | 0.0799 | 0.0645 | *0.0519* | *0.0682* |
| DRB1*13:02~DQB1*06:04/34 | 0.0509 | 0.035 | 0.0359 | 0.0361 | 0.0333 | 0.0101 | 0.0218 | 0.0326 | 0.0433 | 0.0323 | *0.052* | *0.0371* |
| DRB1*13:02~DQB1*06:09 | 0 | 0.0061 | 0.008 | 0.0052 | 0.0167 | 0.0101 | 0 | 0.0054 | 0.0048 | 0 | *0.002* | *0.0051* |
| DRB1*13:03~DQB1*03:01/09/19/21 | 0.0093 | 0.0076 | 0.0034 | 0.0035 | 0 | 0.0101 | 0.0068 | 0.0109 | 0.0111 | 0.0161 | *0.0103* | *0.0083* |
| DRB1*14~DQB1*05:03 | 0.0463 | 0.0363 | 0.0338 | 0.0361 | 0.025 | 0.0703 | 0.0338 | 0.0272 | 0.0481 | 0.0403 | *0.0377* | *0.039* |
| DRB1*14~DQB1*06:03 | 0 | 0 | 0 | 0 | 0.0125 | 0 | 0 | 0 | 0 | 0 | *0* | *0.0003* |
| DRB1*15:01~DQB1*05:01 | 0 | 0 | 0.0034 | 0.0103 | 0 | 0.0076 | 0 | 0 | 0 | 0 | *0.0017* | *0.0015* |
| DRB1*15:01~DQB1*06:02 | 0.1157 | 0.1389 | 0.138 | 0.0962 | 0.0692 | 0.0808 | 0.0737 | 0.1081 | 0.0726 | 0.1532 | *0.1261* | *0.1199* |
| DRB1*15:02~DQB1*06:01 | 0.0046 | 0.0083 | 0 | 0.0108 | 0 | 0.0051 | 0 | 0.0109 | 0.0037 | 0 | *0.0087* | *0.0064* |
| DRB1*16~DQB1*05:02 | 0.0231 | 0.0212 | 0.0236 | 0.0206 | 0.0375 | 0.0505 | 0.0068 | 0.0054 | 0.0148 | 0.0161 | *0.0188* | *0.021* |
