# Supplementary material for: The Heterogeneous HLA Genetic Makeup of the Swiss Population
Source: PLoS One. 2012 Jul 25;7(7):e41400. doi: 10.1371/journal.pone.0041400 (PMC3405111; doi:10.1371/journal.pone.0041400)
Supplement: Supporting Information S5 — Ewens-Watterson's tests. (DOC) [file pone.0041400.s005.doc]

**Supporting Information S5 – Ewens-Watterson’s tests**

Ewens-Watterson's (EW) test is a test for selective neutrality under the infinite allele model. The method used here is an adaptation of the classical EW test to ambiguous data [1,2]. It consists in estimating the p-values of the usual EW test for 1000 non ambiguous random samples generated from the ambiguous data and in using the distribution of these p-values to assess selective neutrality. The program provides a count of the p-values that are significantly lower (or significantly higher, hereafter not included in the table as none were observed) than expected under the neutral hypothesis at the 5% significance level (corrected for multiple testing). If all significant counts are lower (or higher) than the critical levels, then it is reasonable to assume a deficiency (respectively, an excess) of homozygotes.

List of abbreviations used in the below table:

CTS: regional services for blood transfusion

*: BS and ZH not tested because of HWE rejection

§: ZH not tested because of HWE rejection

| Locus | CTS | Estimated homozygosity | Min p-value obtained by resampling | Number of significant p-values (after correction) |
| --- | --- | --- | --- | --- |
| HLA-A | AA | 0.163 | 2.19E-03 | 0 |
|  | BE | 0.134 | 5.40E-04 | 0 |
|  | BS | 0.140 | 3.18E-03 | 0 |
|  | GE | 0.111 | 2.00E-04 | 0 |
|  | GR | 0.132 | 2.09E-01 | 0 |
|  | LG | 0.136 | 2.71E-03 | 0 |
|  | LS | 0.122 | 3.40E-04 | 0 |
|  | LU | 0.127 | 2.50E-04 | 0 |
|  | SG | 0.157 | 6.00E-04 | 0 |
|  | SI | 0.114 | 2.03E-02 | 0 |
|  | ZH | 0.120 | 6.90E-04 | 0 |
| HLA-B | AA | 0.046 | 1.00E-05 | 13 |
|  | BE | 0.057 | 6.70E-04 | 0 |
|  | BS^*^ | --- | --- | --- |
|  | GE | 0.036 | 1.00E-05 | 1 |
|  | GR | 0.048 | 8.00E-05 | 0 |
|  | LG | 0.050 | 4.00E-05 | 0 |
|  | LS | 0.047 | 1.00E-05 | 32 |
|  | LU | 0.046 | 4.20E-04 | 0 |
|  | SG | 0.045 | 1.00E-05 | 5 |
|  | SI | 0.046 | 2.51E-03 | 0 |
|  | ZH^*^ | --- | --- | --- |
| Locus | CTS | Estimated homozygosity | Min p-value obtained by resampling | Number of significant p-values (after correction) |
| HLA-C | AA | 0.088 | 1.00E-05 | 9 |
|  | BE | 0.088 | 1.60E-04 | 0 |
|  | BS | 0.084 | 3.00E-04 | 0 |
|  | GE | 0.077 | 1.00E-05 | 8 |
|  | GR | 0.085 | 2.90E-04 | 0 |
|  | LG | 0.082 | 5.00E-05 | 0 |
|  | LS | 0.089 | 3.00E-05 | 0 |
|  | LU | 0.086 | 1.10E-04 | 0 |
|  | SG | 0.080 | 2.00E-05 | 3 |
|  | SI | 0.089 | 2.00E-05 | 1 |
|  | ZH | 0.083 | 1.00E-05 | 17 |
| HLA-DRB1 | AA | 0.081 | 1.00E-05 | 5 |
|  | BE | 0.088 | 3.70E-04 | 0 |
|  | BS | 0.084 | 3.00E-05 | 0 |
|  | CF | 0.086 | 1.00E-05 | 2 |
|  | FR | 0.088 | 1.20E-04 | 0 |
|  | GE | 0.077 | 2.00E-05 | 3 |
|  | GR | 0.088 | 9.00E-05 | 0 |
|  | LG | 0.074 | 3.00E-05 | 0 |
|  | LS | 0.084 | 2.00E-05 | 6 |
|  | LU | 0.087 | 7.00E-05 | 0 |
|  | SG | 0.083 | 2.00E-05 | 1 |
|  | SI | 0.080 | 1.00E-05 | 206 |
|  | ZH^§^ | --- | --- | --- |
| HLA-DQB1 | AA | 0.110 | 1.00E-05 | 78 |
|  | BE | 0.116 | 7.00E-05 | 0 |
|  | BS | 0.106 | 1.00E-05 | 8 |
|  | GE | 0.126 | 2.00E-04 | 0 |
|  | GR | 0.128 | 4.90E-04 | 0 |
|  | LG | 0.119 | 5.00E-05 | 0 |
|  | LS | 0.133 | 3.00E-05 | 0 |
|  | LU | 0.118 | 9.00E-05 | 0 |
|  | SG | 0.110 | 2.00E-05 | 2 |
|  | SI | 0.136 | 4.00E-05 | 0 |
|  | ZH | 0.118 | 1.10E-04 | 0 |

**References**

1. Nunes JM, Riccio ME, Buhler S, Di D, Currat M, et al. (2010) Analysis of the HLA population data (AHPD) submitted to the 15th International Histocompatibility/Immunogenetics Workshop by using the Gene[rate] computer tools accommodating ambiguous data (AHPD project report). Tissue Antigens 76: 18-30.

2. Nunes JM, Riccio ME, Tiercy JM, Sanchez-Mazas A (2011) Allele frequency estimation from ambiguous data: using resampling schema in validating frequency estimates and in selective neutrality testing. Hum Biol 83: 437-447.
