# Supplementary material for: The Heterogeneous HLA Genetic Makeup of the Swiss Population
Source: PLoS One. 2012 Jul 25;7(7):e41400. doi: 10.1371/journal.pone.0041400 (PMC3405111; doi:10.1371/journal.pone.0041400)
Supplement: Supporting Information S6 — Standardized residuals for two locus haplotypes. (DOC) [file pone.0041400.s006.doc]

**Supporting Information S6 – Standardized residuals for two locus haplotypes**

Standardized residuals for two locus haplotypes within each recruitment region. Only haplotypes with a frequency ≥ 3% in at least one recruitment region are listed. Significant (or large) contributions (i.e. above +2 or below -2) are in bold italic.

Swiss regions were Hardy-Weinberg equilibrium is significantly rejected (after Bonferroni’s correction) for one or both locus are not represented in the tables.

List of abbreviations used in below tables:

AA: Aargau-Solothurn, BE: Bern, BS: Basel, GE: Genève, GR: Graubünden, LG: Lugano (Svizzera  Italiana ), LS: Lausanne (Vaud), LU: Luzern (Zentralschweiz ), SG: St. Gallen (Nordost-Schweiz ), SI: Sion (Valais) and ZH: Zürich. NA: not available.

| **HLA-A-B** |  | | | | | | | | |
| --- | --- | --- | --- | --- | --- | --- | --- | --- | --- |
|  | **Standardized residuals** | | | | | | | | |
| Haplotypes | AA | BE | GE | GR | LG | LS | LU | SG | SI |
| A*01~B*08 | 1.11 | 1.63 | 1.65 | ***6.33*** | 0.98 | 0.26 | ***2.12*** | 1.24 | 0.68 |
| A*01~B*57:01 | 0.41 | 0.01 | 0.22 | 0.19 | 0.01 | 0.24 | -0.07 | 0.68 | 0.71 |
| A*02~B*07 | -0.03 | -0.05 | 0.06 | 0.11 | -0.15 | -0.02 | 0.00 | 0.12 | 0.09 |
| A*02~B*08 | -0.07 | -0.05 | -0.10 | -0.09 | 0.10 | -0.01 | -0.02 | 0.10 | 0.03 |
| A*02~B*15 | 0.35 | 0.31 | 0.19 | 0.38 | 0.12 | 0.18 | 0.20 | 0.26 | 0.16 |
| A*02~B*40:01/55 | 0.43 | 0.23 | 0.35 | 0.04 | 0.22 | 0.26 | 0.20 | 0.20 | 0.32 |
| A*02~B*44:02/19N/27 | 0.49 | 0.25 | 0.47 | 0.11 | 0.28 | 0.28 | 0.14 | 0.31 | 0.02 |
| A*02~B*51 | 0.09 | 0.13 | 0.31 | 0.15 | 0.16 | 0.13 | 0.11 | -0.01 | 0.11 |
| A*03~B*07 | 0.68 | 0.99 | -0.02 | 0.98 | 0.54 | ***3.19*** | 0.85 | 1.75 | 0.51 |
| A*29~B*44:03 | ***3.05*** | 0.42 | 0.56 | 1.84 | 0.74 | 0.47 | ***3.45*** | 0.50 | 0.44 |

**HLA-A-C**

|  | **Standardized residuals** | | | | | | | | | | |
| --- | --- | --- | --- | --- | --- | --- | --- | --- | --- | --- | --- |
| Haplotypes | AA | BE | BS | GE | GR | LG | LS | LU | SG | SI | ZH |
| A*01~C*06:02 | 0.11 | 0.80 | 0.42 | 0.17 | 1.55 | 0.21 | 0.64 | 0.07 | 0.84 | 0.57 | 1.17 |
| A*01~C*07:01/06/18/52 | 0.50 | 1.32 | 1.14 | 1.48 | 1.96 | 0.25 | 0.79 | 0.96 | 0.96 | 0.86 | ***2.85*** |
| A*02~C*03:03/20N | 0.16 | 0.27 | 0.28 | 0.08 | 0.03 | -0.05 | -0.08 | 0.00 | 0.07 | -0.09 | 0.42 |
| A*02~C*03:04 | 0.46 | 0.29 | 0.05 | 0.42 | 0.21 | -0.02 | 0.51 | -0.08 | 0.46 | -0.06 | 0.16 |
| A*02~C*04:01/09N/28/30 | 0.04 | 0.03 | -0.15 | -0.09 | 0.16 | 0.04 | 0.03 | -0.05 | 0.00 | 0.15 | -0.10 |
| A*02~C*05:01/03 | 0.49 | 0.20 | 0.40 | 0.15 | 0.02 | 0.22 | 0.13 | 0.18 | 0.43 | 0.17 | 0.23 |
| A*02~C*06:02 | 0.18 | 0.13 | 0.29 | -0.04 | 0.03 | 0.00 | -0.07 | 0.34 | -0.15 | 0.03 | -0.11 |
| A*02~C*07:01/06/18/52 | -0.02 | -0.09 | 0.07 | -0.03 | -0.01 | -0.02 | 0.05 | -0.01 | -0.07 | 0.07 | -0.06 |
| A*02~C*07:02/50 | -0.09 | 0.05 | 0.00 | 0.27 | 0.28 | 0.05 | -0.08 | -0.04 | 0.35 | 0.16 | 0.11 |
| A*02~C*12:03 | -0.06 | 0.11 | 0.00 | -0.08 | -0.07 | 0.18 | 0.00 | -0.09 | 0.33 | 0.16 | 0.24 |
| A*02~C*14:02 | 0.51 | 0.10 | -0.09 | -0.18 | 0.25 | 0.38 | -0.03 | -0.04 | -0.08 | -0.17 | 0.32 |
| A*03~C*04:01/09N/28/30 | 0.25 | 0.36 | -0.01 | 0.31 | 0.44 | 0.07 | 0.90 | 0.37 | 0.30 | 0.83 | 0.28 |
| A*03~C*07:02/50 | 0.34 | 0.80 | 0.72 | -0.08 | 1.86 | 0.33 | ***2.09*** | 0.68 | 0.80 | 1.99 | 0.29 |
| A*11~C*04:01/09N/28/30 | -0.07 | 0.48 | 0.54 | 0.41 | 0.91 | 0.36 | 0.20 | 0.27 | 0.35 | 0.65 | 0.19 |
| A*23~C*04:01/09N/28/30 | 0.24 | 1.99 | 0.70 | -0.03 | -0.02 | -0.03 | 0.01 | -0.02 | 0.12 | 0.63 | 1.32 |
| A*24~C*04:01/09N/28/30 | 0.09 | 0.05 | 0.84 | -0.09 | -0.07 | -0.02 | 0.91 | 0.14 | 0.54 | -0.05 | 0.32 |
| A*24~C*06:02 | -0.07 | -0.01 | -0.02 | -0.14 | 0.10 | 0.12 | -0.14 | 0.12 | 0.15 | -0.10 | 0.33 |
| A*24~C*07:01/06/18/52 | -0.06 | 0.78 | 0.58 | -0.02 | 0.19 | 0.00 | 0.41 | 0.18 | -0.13 | -0.03 | -0.04 |
| A*24~C*07:02/50 | 0.35 | 0.01 | 0.54 | 1.70 | -0.07 | -0.10 | 0.99 | 0.13 | -0.10 | 0.06 | 0.49 |
| A*29~C*16:01 | 0.56 | 1.49 | 1.01 | 0.54 | 1.32 | 0.62 | 0.13 | 0.86 | 0.51 | ***2.58*** | 0.62 |
| A*30:01/24~C*0602 | 0.10 | 0.26 | 0.31 | 0.45 | 0.62 | -0.03 | 0.10 | 1.04 | 0.41 | 0.99 | ***3.26*** |
| A*30:02~C*05:01/03 | NA | 0.79 | NA | 1.74 | NA | -0.03 | -0.01 | 0.51 | NA | 0.11 | 0.13 |
| A*33~C*08:02 | 0.70 | 0.46 | 0.79 | -0.01 | -0.01 | 1.08 | -0.02 | 0.16 | 0.05 | -0.01 | 0.30 |

**HLA-A-DRB1**

|  |  | | | | | | | | | |
| --- | --- | --- | --- | --- | --- | --- | --- | --- | --- | --- |
|  | **Standardized residuals** | | | | | | | | | |
| Haplotypes | AA | BE | BS | GE | GR | LG | LS | LU | SG | SI |
| A*01~DRB1*03 | 0.47 | 0.25 | 0.22 | 1.30 | -0.06 | 0.09 | 0.22 | -0.08 | 0.44 | 1.55 |
| A*01~DRB1*11 | -0.22 | 1.00 | -0.03 | 0.33 | -0.01 | 0.15 | 0.62 | -0.03 | 0.14 | 0.05 |
| A*02~DRB1*01:01 | -0.01 | 0.12 | 0.07 | 0.01 | 0.19 | 0.15 | 0.11 | -0.08 | 0.30 | 0.07 |
| A*02~DRB1*03 | -0.02 | 0.04 | 0.04 | -0.14 | -0.09 | 0.00 | 0.15 | -0.05 | 0.23 | -0.07 |
| A*02~DRB1*04 | 0.21 | 0.21 | 0.28 | 0.03 | 0.33 | 0.09 | 0.13 | 0.07 | 0.06 | 0.22 |
| A*02~DRB1*07 | 0.12 | 0.16 | 0.32 | 0.17 | 0.27 | 0.08 | -0.04 | -0.05 | 0.02 | -0.03 |
| A*02~DRB1*08 | 0.55 | 0.21 | 0.09 | 0.08 | 0.26 | 0.31 | 0.05 | 0.18 | 0.28 | 0.13 |
| A*02~DRB1*11 | -0.03 | -0.03 | -0.05 | -0.02 | 0.03 | -0.04 | 0.21 | 0.11 | 0.04 | 0.04 |
| A*02~DRB1*13:01 | 0.10 | 0.31 | 0.31 | -0.09 | 0.10 | 0.17 | 0.15 | 0.13 | 0.25 | 0.24 |
| A*02~DRB1*13:02 | 0.33 | 0.12 | 0.00 | 0.25 | 0.16 | -0.08 | 0.18 | 0.24 | 0.11 | 0.06 |
| A*02~DRB1*14 | 0.08 | 0.04 | 0.30 | -0.03 | 0.03 | 0.25 | 0.02 | -0.03 | -0.05 | -0.04 |
| A*02~DRB1*15:01 | 0.27 | 0.06 | 0.06 | 0.24 | 0.01 | 0.09 | 0.09 | 0.39 | 0.41 | -0.10 |
| A*03~DRB1*01:01 | -0.02 | 0.11 | 0.27 | 0.55 | 0.54 | 0.16 | 0.01 | 0.12 | -0.17 | -0.02 |
| A*03~DRB1*11 | -0.09 | ***2.27*** | -0.16 | 0.44 | 0.57 | 1.28 | 0.74 | 1.52 | 0.82 | -0.02 |
| A*03~DRB1*15:01 | 0.17 | 0.00 | -0.12 | -0.05 | ***3.39*** | 0.18 | ***2.16*** | -0.07 | 0.00 | 1.10 |
| A*24~DRB1*11 | 0.08 | 1.08 | -0.07 | 0.45 | 0.32 | ***2.29*** | 0.38 | 0.20 | 0.53 | 1.44 |
| A*29~DRB1*07 | 0.01 | 0.75 | 0.35 | 0.45 | 0.87 | 0.27 | 0.04 | 0.35 | 0.16 | 0.26 |
| A*30:02~DRB1*03 | NA | 0.40 | NA | 0.46 | -0.02 | 0.21 | 0.06 | -0.01 | NA | 0.02 |
| A*68~DRB1*11 | -0.04 | 0.22 | -0.16 | 0.24 | 0.06 | 0.98 | -0.06 | 0.27 | -0.09 | 0.40 |

**HLA-B-C**

|  |  | | | | | | | | |
| --- | --- | --- | --- | --- | --- | --- | --- | --- | --- |
|  | **Standardized residuals** | | | | | | | | |
| Haplotypes | AA | BE | GE | GR | LG | LS | LU | SG | SI |
| B*07~C*0702/C*0750 | ***2.02*** | 0.97 | 0.96 | 0.82 | 0.53 | 0.62 | 0.90 | 0.93 | ***3.14*** |
| B*08~C*07:01/06/18/52 | 0.76 | 1.16 | 0.62 | ***2.13*** | ***2.62*** | ***2.89*** | 0.73 | ***2.59*** | ***2.27*** |
| B*13:02~C*06:02 | 1.31 | 0.40 | 0.08 | ***2.46*** | 0.70 | 0.61 | ***2.13*** | 0.90 | 0.28 |
| B*14:02~C*08:02 | 0.47 | 0.43 | ***3.58*** | 0.56 | ***2.67*** | 0.45 | 0.56 | 0.38 | 0.38 |
| B*15~C*03:03/20N | 0.68 | 0.48 | 1.72 | 0.25 | 0.70 | 0.35 | 0.27 | 0.68 | 0.63 |
| B*18~C*05:01/03 | 0.09 | 0.78 | 1.89 | -0.01 | 0.17 | 0.23 | 1.12 | 0.20 | 0.60 |
| B*18~C*07:01/06/18/52 | 0.60 | 1.06 | 0.57 | ***2.79*** | 0.50 | 0.29 | 1.68 | 0.73 | 0.26 |
| B*35:01/40N/42/57/94~C*04:01/09N/28/30 | 1.32 | ***2.06*** | 1.33 | 0.65 | ***2.02*** | 1.93 | ***2.08*** | 0.36 | 0.82 |
| B*35:03/70~C*04:01/09N/28/30 | 0.35 | 0.45 | 1.32 | 0.88 | -0.01 | 1.25 | 0.29 | 1.67 | 1.56 |
| B*38:01~C*12:03 | 0.92 | 0.80 | 1.12 | 0.59 | 0.54 | 0.26 | 0.11 | 0.95 | 0.26 |
| B*40:01/55~C*03:04 | 0.41 | ***5.04*** | 1.35 | 0.17 | 0.55 | 0.84 | 1.26 | 0.62 | 0.35 |
| B*44:02/19N/27~C*05:01/03 | 0.59 | ***6.13*** | 1.14 | ***3.11*** | 0.69 | 1.05 | 0.59 | 1.51 | 0.84 |
| B*44:03~C*04:01/09N/28/30 | 0.91 | 0.28 | 0.07 | 0.47 | -0.01 | 1.52 | 0.56 | 0.62 | 1.78 |
| B*44:03~C*16:01 | 0.26 | 0.97 | 0.56 | 1.60 | 1.33 | 0.44 | 1.80 | 0.61 | ***2.10*** |
| B*49:01~C*07:01/06/18/52 | 0.27 | ***2.21*** | 0.16 | 0.71 | 1.95 | 0.81 | 1.83 | 0.28 | 0.21 |
| B*51~C*01:02 | 0.09 | 0.80 | 0.42 | 0.70 | -0.02 | 0.08 | 0.20 | 0.27 | 0.23 |
| B*51~C*14:02 | 0.48 | 1.00 | 0.08 | 0.72 | 0.41 | 0.47 | 0.37 | 0.15 | 0.01 |
| B*51~C*15:02/13 | 0.58 | ***2.51*** | 1.10 | 0.22 | 0.32 | 1.10 | 0.59 | 0.48 | 0.62 |
| B*57:01~C*06:02 | 1.05 | 1.93 | ***3.19*** | 0.93 | 0.73 | 1.33 | 1.21 | 0.56 | 0.76 |

**HLA-B-DRB1**

|  |  | | | | | | | | |
| --- | --- | --- | --- | --- | --- | --- | --- | --- | --- |
|  | **Standardized residuals** | | | | | | | | |
| Haplotypes | AA | BE | GE | GR | LG | LS | LU | SG | SI |
| B*07~DRB1*15:01 | 1.20 | 0.46 | 1.11 | 1.20 | 0.86 | 0.54 | 0.51 | 0.61 | ***2.88*** |
| B*08~DRB1*03 | 1.53 | ***8.96*** | ***2.10*** | 0.66 | 0.65 | 0.50 | ***3.15*** | 1.37 | 0.65 |
| B*15~DRB1*04 | 0.05 | 0.45 | 0.00 | 0.52 | 0.75 | 0.65 | 0.50 | 0.09 | 0.10 |
| B*15~DRB1*11 | -0.09 | 0.20 | 0.10 | 0.56 | 0.34 | 0.61 | 0.63 | 0.00 | 0.76 |
| B*35:01/40N/42/57/94~DRB1*01:01 | 0.20 | 0.52 | 1.20 | 0.75 | 0.39 | 0.88 | 1.50 | 0.95 | 1.61 |
| B*44:02/19N/27~DRB1*04 | 0.08 | 1.53 | 0.55 | ***2.03*** | 0.07 | 0.35 | 0.59 | 0.54 | 0.35 |
| B*44:03~DRB1*07 | 0.31 | 1.21 | 0.46 | 1.87 | 1.33 | 0.84 | 0.68 | 0.13 | 0.45 |
| B*51~DRB1*11 | 0.24 | 0.48 | 0.36 | 0.17 | 0.21 | 0.36 | 0.44 | 0.45 | 0.22 |
| B*57:01~DRB1*07 | 1.00 | 0.69 | 0.40 | 0.13 | 0.95 | 0.38 | 0.76 | 0.39 | 1.11 |

**HLA-C-DRB1**

|  |  | | | | | | | | | |
| --- | --- | --- | --- | --- | --- | --- | --- | --- | --- | --- |
|  | **Standardized residuals** | | | | | | | | | |
| Haplotypes | AA | BE | BS | GE | GR | LG | LS | LU | SG | SI |
| C*04:01/09N/28/30~DRB1*01:01 | 0.19 | 0.85 | ***2.31*** | 0.85 | 0.38 | 0.16 | 0.02 | 0.37 | 1.03 | 0.12 |
| C*04:01/09N/28/30~DRB1*11 | -0.03 | 0.14 | 1.03 | 0.56 | 0.07 | 0.91 | 0.59 | -0.01 | 0.43 | 0.29 |
| C*04:01/09N/28/30~DRB1*15:01 | 0.02 | 0.21 | 0.30 | -0.03 | 0.30 | 0.26 | 0.17 | -0.04 | -0.03 | -0.12 |
| C*05:01/03~DRB1*04 | 0.32 | 0.03 | 1.02 | 0.26 | 0.53 | 0.25 | -0.08 | 0.21 | 0.35 | 0.09 |
| C*05:01/03~DRB1*11 | 0.08 | 0.01 | 0.58 | 0.03 | 0.30 | 0.08 | 0.23 | -0.11 | -0.07 | 0.65 |
| C*06:02~DRB1*07 | 1.48 | ***4.37*** | 0.41 | 0.30 | ***3.34*** | 0.56 | 0.57 | 0.81 | 0.91 | 0.86 |
| C*07:01/06/18/52~DRB1*03 | 0.25 | 0.47 | 0.48 | 0.30 | 1.33 | 0.27 | 0.62 | ***2.29*** | 0.28 | ***2.60*** |
| C*07:01/06/18/52~DRB1*11 | 1.31 | 0.04 | -0.03 | -0.03 | 0.25 | -0.02 | -0.12 | 1.74 | 0.00 | 0.39 |
| C*07:02/50~DRB1*15:01 | ***2.86*** | ***3.80*** | 1.19 | 0.32 | 0.40 | 1.33 | 1.23 | 1.72 | ***2.94*** | 0.51 |
| C*16:01~DRB1*07 | 1.67 | 1.66 | 0.66 | ***3.08*** | 0.72 | 0.92 | 1.26 | 1.95 | 0.57 | 0.63 |

**HLA-DRB1-DQB1**

|  |  | | | | | | | | | |
| --- | --- | --- | --- | --- | --- | --- | --- | --- | --- | --- |
|  | **Standardized residuals** | | | | | | | | | |
| Haplotypes | AA | BE | BS | GE | GR | LG | LS | LU | SG | SI |
| DRB1*01:01~DQB1*05:01 | ***3.90*** | ***2.41*** | ***2.33*** | ***2.92*** | 0.73 | 1.69 | 0.67 | ***2.68*** | ***4.63*** | 0.74 |
| DRB1*03~DQB1*02:01 | ***2.70*** | ***3.54*** | 0.75 | ***4.10*** | 1.35 | ***3.91*** | 1.82 | 0.46 | 0.57 | 1.91 |
| DRB1*04~DQB1*03:01/09/19/21 | 0.33 | 1.02 | 0.86 | 0.60 | 0.58 | 1.16 | -0.09 | 1.79 | ***2.37*** | 0.41 |
| DRB1*04~DQB1*03:02 | ***3.86*** | 0.56 | ***4.23*** | 0.58 | 0.87 | 0.52 | 0.54 | 0.66 | ***4.89*** | 0.95 |
| DRB1*07~DQB1*02:02 | ***3.08*** | ***3.37*** | 0.54 | 0.61 | 1.23 | 0.60 | ***2.55*** | 0.72 | 0.48 | 1.71 |
| DRB1*07~DQB1*03:03 | 0.41 | 0.13 | 0.38 | 0.48 | 0.05 | 0.43 | 0.10 | 0.35 | 0.30 | 0.00 |
| DRB1*08~DQB1*04:02 | 1.57 | 1.68 | 0.36 | 0.22 | 0.99 | ***3.13*** | 0.26 | 0.45 | 0.44 | 1.27 |
| DRB1*11~DQB1*03:01/09/19/21 | 0.69 | 0.65 | 1.32 | 0.67 | 0.63 | 0.60 | ***4.43*** | 0.65 | ***4.95*** | 0.61 |
| DRB1*13:01~DQB1*06:03 | 0.94 | ***2.27*** | 1.13 | 0.35 | ***2.46*** | ***3.48*** | 0.92 | 0.47 | 0.57 | 0.71 |
| DRB1*13:02~DQB1*06:04/34 | 1.76 | 1.55 | 0.55 | 0.32 | 0.61 | 0.10 | 1.23 | 0.32 | 0.46 | 0.30 |
| DRB1*14~DQB1*05:03 | 0.76 | 0.93 | 0.36 | 0.35 | 0.75 | ***3.59*** | 0.26 | 0.33 | 0.51 | 0.56 |
| DRB1*15:01~DQB1*06:02 | 1.35 | ***10.43*** | ***5.64*** | 0.83 | ***2.11*** | 0.73 | 1.34 | 0.69 | 0.58 | ***2.25*** |
| DRB1*16~DQB1*05:02 | 0.29 | 0.74 | ***2.00*** | 0.23 | 1.48 | ***3.21*** | 0.97 | 0.68 | 0.21 | 0.26 |
