# Supplementary material for: The Heterogeneous HLA Genetic Makeup of the Swiss Population
Source: PLoS One. 2012 Jul 25;7(7):e41400. doi: 10.1371/journal.pone.0041400 (PMC3405111; doi:10.1371/journal.pone.0041400)
Supplement: Supporting Information S7 — Pairwise FST's among Swiss regions. (DOC) [file pone.0041400.s007.doc]

**Supporting Information S7 – Pairwise F_ST_’s among Swiss regions**

Number of pairwise significant F_ST_’s (p < 0.05) for each recruitment region relatively to the other centers at a given locus. Mean and standard deviation for the 5 loci are also indicated. DRB1 “all” corresponds to all donors typed at the HLA-DRB1 locus in the registry, while DRB1 “high” corresponds to the subset of donors typed at high resolution from July 2008 (see Material and methods).

|  | A (n=2488) | B (n=2924) | C (n=3512) | DRB1 "all" (n=21607) | DRB1 "high" (n=1366) | DQB1 (n=1808) | mean | sd |
| --- | --- | --- | --- | --- | --- | --- | --- | --- |
| AA | 5 | 3 | 3 | 7 | 1 | 0 | 3.17 | 2.56 |
| BE | 3 | 10 | 2 | 10 | 2 | 3 | 5.00 | 3.90 |
| BS | 0 | not in HWE | 1 | 9 | 2 | 3 | 3.00 | 3.54 |
| CF | --- | --- | --- | 11 | --- | --- | --- | --- |
| FR | --- | --- | --- | 4 | --- | --- | --- | --- |
| GE | 1 | 8 | 3 | 10 | 2 | 0 | 4.00 | 4.05 |
| GR | 3 | 7 | 8 | 8 | 5 | 3 | 5.67 | 2.34 |
| LG | 1 | 10 | 6 | 12 | 3 | 3 | 5.83 | 4.36 |
| LS | 6 | 8 | 0 | 7 | 0 | 2 | 3.83 | 3.60 |
| LU | 1 | 8 | 1 | 10 | 5 | 0 | 4.17 | 4.17 |
| SG | 1 | 3 | 2 | 9 | 2 | 0 | 2.83 | 3.19 |
| SI | 2 | 5 | 0 | 10 | 7 | 0 | 4.00 | 4.05 |
| ZH | 1 | not in HWE | 2 | not in HWE | 5 | 2 | 2.50 | 1.73 |
|  |  |  |  |  |  |  |  |  |
| AA: Aargau-Solothurn, BE: Bern, BS: Basel, GE: Genève, GR: Graubünden, LG: Lugano (Svizzera  Italiana ), LS: Lausanne (Vaud), LU: Luzern (Zentralschweiz ), SG: St. Gallen (Nordost-Schweiz ), SI: Sion (Valais) and ZH: Zürich. ---: not enough data available. | | | | | | | | |
