# Supplementary material for: The Heterogeneous HLA Genetic Makeup of the Swiss Population
Source: PLoS One. 2012 Jul 25;7(7):e41400. doi: 10.1371/journal.pone.0041400 (PMC3405111; doi:10.1371/journal.pone.0041400)
Supplement: Supporting Information S8 — Multidimensional scaling analyses (MDS) for HLA-A, -B, -C, -DRB1 and -DQB1. (DOC) [file pone.0041400.s008.doc]

**Supporting Information S8 – Multidimensional scaling analyses (MDS) for HLA-A, -B, -C, -DRB1 and -DQB1**

Multidimensional scaling (MDS) analyses of Swiss regions based on Reynold’s genetic distances computed for each of 5 HLA loci (HLA-A, -B, -C, -DRB1 and -DQB1). Two graphics are provided for HLA-DRB1, one using all donors typed at this locus in the registry (“all”) and one using the subset of donors typed at high resolution from July 2008 (“high”, see Material and methods). Symbols refer to the language spoken in each region.

Swiss regions where Hardy-Weinberg equilibrium is significantly rejected (after Bonferroni’s correction) are not represented on the graphics (i.e. BS and ZH at HLA-B and ZH at HLA-DRB1 “all”, respectively).

List of abbreviations used in the below graphics:

AA: Aargau-Solothurn, BE: Bern, BS: Basel, GE: Genève, GR: Graubünden, LG: Lugano (Svizzera Italiana), LS: Lausanne (Vaud), LU: Luzern (Zentralschweiz), SG: St. Gallen (Nordost-Schweiz), SI: Sion (Valais), ZH: Zürich.

**HLA-A (stress = 0.124)**

**HLA-B (stress = 0.096)**

**HLA-C (stress = 0.164)**

**HLA-DRB1 “all” (stress = 0.078)**

**HLA-DRB1 “high” (stress = 0.094)**

**HLA-DQB1 (stress = 0.125)**
