# Supplementary material for: The Heterogeneous HLA Genetic Makeup of the Swiss Population
Source: PLoS One. 2012 Jul 25;7(7):e41400. doi: 10.1371/journal.pone.0041400 (PMC3405111; doi:10.1371/journal.pone.0041400)
Supplement: Supporting Information S9 — Map of the 13 regional blood transfusion services of the SBSC registry and the geographic or linguistic structures tested by ANOVA. (DOC) [file pone.0041400.s009.doc]

**Supporting Information S9 – Map of the 13 regional blood transfusion services of the SBSC registry and the geographic or linguistic structures tested by ANOVA**

Map of the 13 regional blood transfusion services of the Swiss Blood Stem Cells registry and the different geographic or linguistic groups tested by Analysis of Variance (ANOVA) on the basis of HLA allelic frequencies (i.e. single locus or combined multiple loci analyses, see Table 3 and Supporting Information S10). A: western *versus* eastern Switzerland; B: far-western *versus* centre *versus* northern *versus* southern *versus* far-eastern Switzerland (FR was grouped either with GE and LS (far-west) or with BE & LU (centre), with no changes in the results); C: the central plateau and Jura *versus* the Alps; D: French-speaking *versus* German-speaking *versus* Italian-speaking areas. Bi- or tri-lingual cantons/regions were grouped according to the language spoken by the majority of their native speakers. The fourth official Swiss language, Romansh, was not taken into account as it is only spoken by a minority of the Graubünden (GR) inhabitants. AA: Aargau-Solothurn, BE: Bern, BS: Basel, CF: La Chaux-de-Fonds (Neuchâtel and Jura), FR: Fribourg, GE: Genève, GR: Graubünden, LG: Lugano (Svizzera Italiana), LS: Lausanne (Vaud), LU: Luzern (Zentralschweiz), SG: St. Gallen (Nordost-Schweiz), SI: Sion (Valais) and ZH: Zürich.
