# Supplementary material for: The Heterogeneous HLA Genetic Makeup of the Swiss Population
Source: PLoS One. 2012 Jul 25;7(7):e41400. doi: 10.1371/journal.pone.0041400 (PMC3405111; doi:10.1371/journal.pone.0041400)
Supplement: Supporting Information S10 — Single locus ANOVA's. (DOC) [file pone.0041400.s010.doc]

**Supporting Information S10 – Single locus ANOVA’s**

Genetic diversity within geographic or linguistic groups (F_SC_) and among geographic or linguistic groups (F_CT_) and their significance, for different partitions defined a priori.

List of abbreviations used in below table:

CTS: regional services for blood transfusion

#: CF and FR not tested because of small sample sizes

*: BS and ZH not tested because of HWE rejection

§: ZH not tested because of HWE rejection

n: number of donors

N: number of geographic or linguistic groups tested

NA: The number of donors available at each of the 5 loci is used for the computations.

| **West *vs* East** | | | | | |
| --- | --- | --- | --- | --- | --- |
|  | CTS | n | N | F_CT_ (%) | F_SC_ (%) |
| 5 loci | 9/11/12 | NA | 2 | 0.0013 ^(P=0.441)^ | 0.141 ^(P=1.47E-12)^ |
| HLA-A | 11^#^ | 2488 | 2 | 0.07 ^(P=0.054)^ | 0.11 ^(P=0.015)^ |
| HLA-B | 9^#*^ | 2328 | 2 | 0.01 ^(P=0.484)^ | 0.25 ^(P<0.0001)^ |
| HLA-C | 11^#^ | 3512 | 2 | 0 ^(P=0.533)^ | 0.09 ^(P=0.0006)^ |
| HLA-DRB1 | 12^§^ | 16732 | 2 | 0 ^(P=0.813)^ | 0.11 ^(P<0.0001)^ |
| HLA-DQB1 | 11^#^ | 1808 | 2 | 0 ^(P=0.595)^ | 0.14 ^(P=0.016)^ |
|  |  |  |  |  |  |
| **Far-West *vs* Centre *vs* South *vs* North *vs* Far-East** | | | | | |
|  | CTS | n | N | F_CT_ (%) | F_SC_ (%) |
| 5 loci | 9/11/12 | NA | 5 | 0.054 ^(P=0.012)^ | 0.094 ^(P=2.99E-10)^ |
| HLA-A | 11^#^ | 2488 | 5 | 0.02 ^(P=0.392)^ | 0.13 ^(P=0.022)^ |
| HLA-B | 9^#*^ | 2328 | 2 | 0.07 ^(P=0.161)^ | 0.19 ^(P<0.0001)^ |
| HLA-C | 11^#^ | 3512 | 5 | 0 ^(P=0.436)^ | 0.08 ^(P=0.006)^ |
| HLA-DRB1 | 12^§^ | 16732 | 2 | 0.06 ^(P=0.007)^ | 0.05 ^(P<0.0001)^ |
| HLA-DQB1 | 11^#^ | 1808 | 5 | 0.13 ^(P=0.058)^ | 0 ^(P=0.422)^ |
|  |  |  |  |  |  |
| **Alps *vs* Plateau & Jura** | | | | | |
|  | CTS | n | N | F_CT_ (%) | F_SC_ (%) |
| 5 loci | 9/11/12 | NA | 2 | 0.138 ^(P=0.0001)^ | 0.1 ^(P=1.87E-10)^ |
| HLA-A | 11^#^ | 2488 | 2 | 0.05 ^(P=0.135)^ | 0.12 ^(P=0.003)^ |
| HLA-B | 9^#*^ | 2328 | 2 | 0.05 ^(P=0.143)^ | 0.23 ^(P<0.0001)^ |
| HLA-C | 11^#^ | 3512 | 2 | 0.13 ^(P=0.005)^ | 0.04 ^(P=0.034)^ |
| HLA-DRB1 | 12^§^ | 16732 | 2 | 0.08 ^(P=0.033)^ | 0.07 ^(P<0.0001)^ |
| HLA-DQB1 | 11^#^ | 1808 | 2 | 0.38 ^(P=0.007)^ | 0.03 ^(P=0.268)^ |
|  |  |  |  |  |  |
| **French *vs* German *vs* Italian** | | | | | |
|  | CTS | n | N | F_CT_ (%) | F_SC_ (%) |
| 5 loci | 9/11/12 | NA | 3 | 0.098 ^(P=1.46E-05)^ | 0.095 ^(P=8.64E-11)^ |
| HLA-A | 11^#^ | 2488 | 3 | 0.02 ^(P=0.279)^ | 0.13 ^(P=0.004)^ |
| HLA-B | 9^#*^ | 2328 | 2 | 0.1 ^(P=0.063)^ | 0.2 ^(P<0.0001)^ |
| HLA-C | 11^#^ | 3512 | 3 | 0.08 ^(P=0.032)^ | 0.05 ^(P=0.018)^ |
| HLA-DRB1 | 12^§^ | 16732 | 2 | 0.1 ^(P=0.0001)^ | 0.05 ^(P<0.0001)^ |
| HLA-DQB1 | 11^#^ | 1808 | 3 | 0.19 ^(P=0.03)^ | 0.05 ^(P=0.191)^ |
