# Supplementary material for: The Heterogeneous HLA Genetic Makeup of the Swiss Population
Source: PLoS One. 2012 Jul 25;7(7):e41400. doi: 10.1371/journal.pone.0041400 (PMC3405111; doi:10.1371/journal.pone.0041400)
Supplement: Supporting Information S11 — HLA nomenclature, typing resolution, ambiguities and SPLIT-TEST. (DOC) [file pone.0041400.s011.doc]

**Supporting Information S11 – HLA nomenclature, typing resolution, ambiguities and Split-test**

**HLA nomenclature**

With an extensive polymorphism of more than 7,000 HLA class I and II alleles compiled to date (http://www.ebi.ac.uk/imgt/hla/stats.html), an official nomenclature for HLA genes was first discussed and put in place progressively since the mid of the 1960’s [1]. Initially, the HLA polymorphism was characterized at the antigen level by serological methods (still used, notably for function related studies or in transplantation daily practice), and then, since the mid of the 1990s for class II genes and a bit later for class I genes with the development of the PCR method, by DNA-based typing [2]. These technology advances spanning almost half a century are important, both for a good appraisal of the current HLA official nomenclature [3,4] and to understand how this complex polymorphism is studied.

The figure below summarizes how an allele is named in the current WHO HLA Nomenclature (adapted from http://hla.alleles.org/nomenclature/naming.html).

**HLA typing resolution and ambiguities**

Due to the ever increasing number of alleles discovered, most HLA DNA-based typing protocols (e.g. PCR-SSOP, PCR-SSP, bi-allelic sequencing) cannot cope with this diversity and generate ambiguous outputs (i.e. several different genotypes can explain the reactivity pattern of probes, primers or sequence readings for an individual at a given HLA locus). The use of mono-allelic sequencing and/or the combination of several complementary protocols may allow obtaining unambiguous outputs, but this is both costly and time consuming. New generation sequencing approaches are promising but are not yet ready to be used in routine typing. Thus, it is very uncommon to work with HLA data void of ambiguities.

Depending on their power to discriminate between HLA alleles, typing methods are generally characterized at low (or generic), intermediate, and high (or allelic) resolution. Common and standard procedures for describing and working with typing resolution and ambiguities are currently discussed by HLA experts at the international level [5,6,7,8], and briefly summarized hereafter based on the guidelines of COST Action BM0803 [7].

***Low resolution*** is a DNA-based typing result at the level of the first field of the DNA-based nomenclature (i.e. a generic typing). ***Intermediate resolution*** is defined as a DNA-based typing result that includes a subset of alleles sharing the digits in the first field of their allele name and that excludes some alleles sharing this field. A ***high resolution*** typing result is defined as a set of alleles that specify and encode the same protein sequence for the peptide binding region of an HLA molecule and that exclude alleles that are not expressed as cell-surface proteins. It at least resolves all ambiguities resulting from polymorphisms located within exons 2 and 3 for class I loci, and within exon 2 for class II loci. ***Allelic resolution*** is a DNA-based typing result consistent with a single allele as defined in the WHO HLA Nomenclature.

In practice, typing ambiguities are reported in different types of format containing more or less information related to the observed reactivity pattern at the DNA level. A **list of possible genotypes (e.g.** B*08:01:01G,B*15:18:01 or B*08:21,B*15:93 or B*08:35,B*15:10:01**)** is the most efficient way of reporting ambiguities, but it is not user friendly due to huge numbers of possible allele pairs in certain cases. Abbreviated reports in the form of allele strings (e.g. B*08:01/21/35,B*15:10/18/93) or NMDP codes (e.g. B*08:MDY,B*15:DZBP), see http://bioinformatics.nmdp.org/HLA/Allele_Codes/Allele_Codes.aspx, are more commonly used by laboratories to report their results, but this unfortunately leads to an important loss of information when re-expanded to lists of possible genotypes.

**Split-test**

As described in the material and methods section of the manuscript, the Swiss registry contains data with heterogeneous levels of resolution at one or several loci. Because it was not possible to resolve ambiguous genotypes by further laboratory typing, we decided to develop a program named Split-test to handle these complex data in a preliminary step to frequency estimation. For a given group of alleles (i.e. a generic specificity), it compares the proportions of either non-ambiguous alleles or ambiguous allele groups with the proportion of generic typings. Based on our experience with several thousands of samples for various genetics systems, a ratio of 1:3 between ambiguous or generic typing and non-ambiguous typing gives the best results. Actually, this value can be set in the program, 3 being the default value. A value above that threshold indicates that the number of either non-ambiguous alleles or ambiguous allele groups (or both) is much higher than the number of generic alleles involving that specificity. The program then provides the decision to take into account the ambiguities to estimate allele frequencies (except for particular cases described hereafter). On the other hand, when a value below that threshold is obtained, the program recommends to the user to recode the ambiguous and non-ambiguous alleles involving that specificity to a lower (usually generic) level of resolution prior to frequency estimation. A complementary GNU/Linux bash script called Add-ins was also written to summarize the data in hand and to refine the decisions of Split-test in particular cases: for each generic specificity, Add-ins provides the list of NMDP codes, of ambiguous alleles included in these codes, of unambiguously-typed alleles, and of alleles identical for PBR-coding exons observed in the input data. In such situations where the decision was to use the ambiguities for frequency estimation, NMDP codes reported in the SBSC registry were automatically checked with the allele code list available at http://bioinformatics.nmdp.org/HLA/Allele_Codes/Allele_Codes.aspx and translated into allele groups (e.g. A*01:AB translated into A*01:01/A*01:02). These allele groups were then expanded to all possible allele pairs explaining the ambiguous genotype (e.g. four possible combinations for the ambiguous genotype A*01:01/02,A*02:01/09) using the uniformate program of the Gene[RATE] tools [9,10]. The only exception was for alleles identical to each other in the PBR-coding exons (i.e. exons 2 and 3 for class I, and exon 2 for class II loci), which were conserved as blocks of undistinguished alleles when they were not explicitly discriminated in the registry data (e.g. HLA-DRB1*14:01/54 were considered as an ambiguous allele block, while DQB1*02:01 02:02 and 02:04 were discriminated). Similarly, generic typings (e.g. B*44:XX) were translated into the alleles observed in the registry for these specificities (e.g. the B*44 alleles detected unambiguously and the B*44 alleles comprised in the NMDP codes, see Add-ins) plus an “unspecified” allele accounting for all other alleles of these specificities (e.g. B*44 alleles possibly missed during typing). These lists of alleles were automatically expended to all possible combinations of allele pairs, as described above.

The decisions taken for the different regional services were compared among each other, and were merged to treat at once the data of the whole national registry. In that case, a clear majority of decisions either toward working with ambiguities or toward recoding to a lower resolution was the determining factor and the most conservative choice.

To analyze HLA haplotypes, due to the complexity of the data increasing with each supplementary locus added in the computations, the criterion applied for single locus analyses was adapted as follows to avoid artifacts in frequency estimation: for 2-locus haplotypes, ambiguous data were used for a given locus only if 10 of the 11 (or 13) regional services exhibited a scale ratio above 3 with Split-test, while data were recoded to a generic level of resolution in all other cases; for 3-locus A-B-DRB1 haplotypes, only generic data were used, while for A-B-C and 4-locus A-B-C-DRB1 haplotypes, generic data were used for loci A, B and DRB1, while ambiguities were taken into account for locus C.

The genotype data generated through Split-test recommendations (i.e. consisting of individuals with either one unambiguous genotype (at allelic or low resolution) or a list of possible genotypes) were in a successive step analyzed with the EM-algorithm implemented in Gene[RATE] tools (http://geneva.unige.ch/generate/), as described in the material and methods section.

**References**

1. Marsh S, Parham P, Barber L (2000) The HLA FactsBook: Academic Press. 398 p.

2. Bunce M, Young N, Welsh K (1997) Molecular HLA typing-the brave new world. Transplantation 64: 1505-1513.

3. Marsh SG, Albert ED, Bodmer WF, Bontrop RE, Dupont B, et al. (2010) Nomenclature for factors of the HLA system, 2010. Tissue Antigens 75: 291-455.

4. Marsh SG, Albert ED, Bodmer WF, Bontrop RE, Dupont B, et al. (2010) An update to HLA nomenclature, 2010. Bone Marrow Transplant 45: 846-848.

5. Nunes E, Heslop H, Fernandez-Vina M, Taves C, Wagenknecht DR, et al. (2011) Definitions of histocompatibility typing terms. Blood 118: e180-183.

6. Nunes E, Heslop H, Fernandez-Vina M, Taves C, Wagenknecht DR, et al. (2011) Definitions of histocompatibility typing terms: Harmonization of Histocompatibility Typing Terms Working Group. Hum Immunol 72: 1214-1216.

7. Sanchez-Mazas A, Vidan-Jeras B, Nunes J, Fischer G, Little A, et al. (2012) Strategies to work with HLA data in human populations for histocompatibility, clinical transplantation, epidemiology and population genetics: HLA-NET methodological recommendations. Int J Immunogenet.

8. Hollenbach JA, Mack SJ, Gourraud PA, Single RM, Maiers M, et al. (2011) A community standard for immunogenomic data reporting and analysis: proposal for a STrengthening the REporting of Immunogenomic Studies statement. Tissue Antigens 78: 333-344.

9. Nunes J (2006) Generate: tools for analysis and handling of data with ambiguities. Laboratory of Anthropology, Genetics and Peopling History, University of Geneva, Switzerland.

10. Nunes J (2006) Tools for efficient HLA data handlings. In: Hansen J, editor. Immunobiology of the Human MHC: Proceedings of the 13th International Histocompatibility Workshop and Conference. Seattle, WA: IHWG Press. pp. 143-147.
